# Supplementary material for: Expert-generated standard practice elements for evidence-based home visiting programs using a Delphi process
Source: PLoS One. 2022 Oct 17;17(10):e0275981. doi: 10.1371/journal.pone.0275981 (PMC9576067; doi:10.1371/journal.pone.0275981)
Supplement: S1 File — (PDF) [file pone.0275981.s001.pdf]

JOHNS HOPKINS
BLOOMBERG SCHOOL
of PUBLIC HEALTH

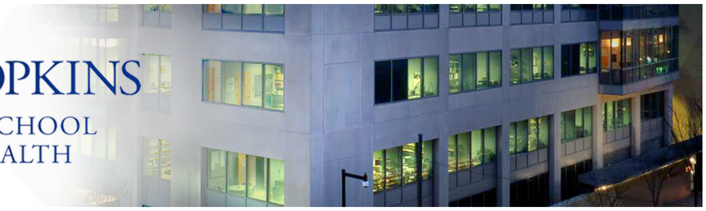

Default Question Block

Thank you for agreeing to participate in this expert elicitation process. The overall goal is to create a taxonomy of standard practice elements across early childhood home visiting to inform Family Spirit programming and provide information to other EBHV models for their own precision home visiting efforts.

We are asking you to participate in an open-ended questionnaire to elicit practice elements that you think are important to early childhood home visiting.

We define **practice elements** as the techniques and strategies used in early childhood home visiting as part of a larger intervention. For example, relaxation could be a technique used to address the domain of reducing child maltreatment.

Panel members' responses to this initial questionnaire will inform the next video conference where we will identify commonalities, clarify meaning, and solicit additional thoughts.

Please list out all practice elements in early childhood home visiting that you can think of. We have left room for up to 20 practice elements. If you think of more after that, you can enter them in the text field we've provided on the next page.

Practice element #1

Practice element #2

Practice element #3

Practice element #4

Practice element #5

Practice element #6

Practice element #7

Practice element #8

Practice element #9

Practice element #10

Practice element #11

Practice element #12

Practice element #13

Practice element #14

Practice element #15

Practice element #16

Practice element #17

Practice element #18

Practice element #19

Practice element #20

If you have additional practice elements to add, please enter them in the space below.

Are any of the practice elements you previously listed **critical for all home visiting programs that serve tribal communities?** Please drag them from the left over to the right.

Items

${q://QID3/ChoiceTextEntryValue}

${q://QID4/ChoiceTextEntryValue}

${q://QID5/ChoiceTextEntryValue}

${q://QID6/ChoiceTextEntryValue}

${q://QID7/ChoiceTextEntryValue}

${q://QID8/ChoiceTextEntryValue}

${q://QID9/ChoiceTextEntryValue}

${q://QID10/ChoiceTextEntryValue}

${q://QID11/ChoiceTextEntryValue}

${q://QID12/ChoiceTextEntryValue}

${q://QID13/ChoiceTextEntryValue}

${q://QID14/ChoiceTextEntryValue}

Critical to tribal home visiting

${q://QID15/ChoiceTextEntryValue}

${q://QID16/ChoiceTextEntryValue}

${q://QID17/ChoiceTextEntryValue}

${q://QID18/ChoiceTextEntryValue}

${q://QID19/ChoiceTextEntryValue}

${q://QID20/ChoiceTextEntryValue}

${q://QID21/ChoiceTextEntryValue}

${q://QID22/ChoiceTextEntryValue}

If you listed more than 20 practice elements on the previous page, they are copied below. In the next item, identify which of these you also think are **critical for all home visiting programs that serve tribal communities?**

${q://QID23/ChoiceTextEntryValue}

Use the space below to enter the additional practice elements listed above that you think are **critical for tribal home visiting.** If there are no additional items to add, leave this field blank.

If you can think of any practice elements that are specific to tribal home visiting but not already mentioned, please list them below. We have left room for up to 5 tribal-specific practice elements. If you think of more after that, you can enter them in the text field we've provided on the next page.

Tribal practice element #1

Tribal practice element #2

Tribal practice element #3

Tribal practice element #4

Tribal practice element #5

If you have additional tribal-specific practice elements to add, please enter them in the space below.

Enter the name of the person filling out this questionnaire only after you are sure you can't think of any more practice elements to add.

We appreciate the time you took to complete this initial questionnaire. Before our next video conference, we will synthesize responses from all panel members.

Powered by Qualtrics
