# Supplementary material for: Expert-generated standard practice elements for evidence-based home visiting programs using a Delphi process
Source: PLoS One. 2022 Oct 17;17(10):e0275981. doi: 10.1371/journal.pone.0275981 (PMC9576067; doi:10.1371/journal.pone.0275981)
Supplement: S2 File — (PDF) [file pone.0275981.s002.pdf]

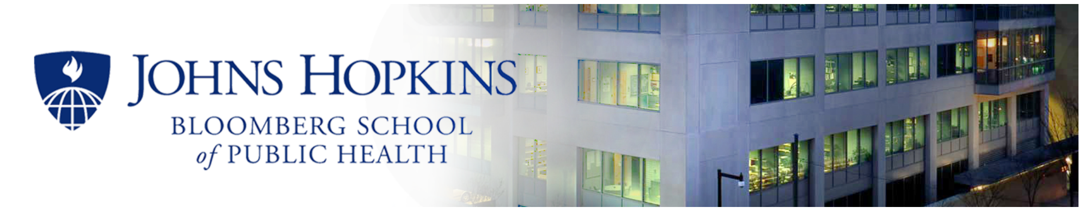

Default Question Block

The purpose of this questionnaire is to to group the practice elements by broad categories. Elements can be grouped into multiple categories.

At the end of the questionnaire, you will have a chance to provide additional feedback and add to the list of practice elements if needed.

Per feedback from the group, we will use the following definitions of elements categories. Please note that these are not listed in terms of priority:

Model philosophy elements
The tenets of an EBHV that drive the other components of home visiting, including a model's theory of change and cultural lifeways.
Example: we believe parents should be a child's first and best teacher; thus, our theory of change involves home visitors teaching parents who teach their children
Program implementation elements
Strategies, techniques, structures, and processes (e.g. program design) at the model/organizational/site level that relate to ensuring successful delivery of the EBHV, including buy-in (community, agency, home visitor), staff training, supervision, fidelity, funding, and payment structures.
Example: Program leadership; Developing content mastery and delivery skills for home visitors, recruitment and retention of home visitors, training, and supervision; Fidelity assessment
Home visiting content elements
The content (i.e. the "what") that is conveyed by home visitors to their clients during service delivery.

Example: Home visitors engaging in information sharing for their clients to cover a broad range of topics (e.g. child development, pregnancy, labor and delivery, breastfeeding)

Home visiting process/ delivery elements

The strategies and techniques home visitors use during service delivery with their clients.

Example: Home visitor and client role play, skills practice, test taking, recitation, videos and discussion

Home visitor personal characteristics

Home visitor characteristics that may contribute to improvement in client outcomes but that aren't typically specified in a model's theoretical/ conceptual framework or their content. These are aspects of the home visitor that are not an explicit part of the model.

Example: Home visitor flexibility and adaptability, cultural humility, maturity, patience

---

Which broad category does **providing clients with linkages to services** fall into? Check all that apply.

- [ ] Model philosophy element
- [ ] Program implementation element
- [ ] Home visiting content element
- [ ] Home visiting process/ delivery element
- [ ] Home visitor personal characteristic

Model philosophy elements

The tenets of an EBHV that drive the other components of home visiting, including a model's theory of change and cultural lifeways.

Example: we believe parents should be a child's first and best teacher; thus, our theory of change involves home visitors teaching parents who teach their children

Program implementation elements

Strategies, techniques, structures, and processes (e.g. program design) at the model/organizational/site level that relate to ensuring successful delivery of the

EBHV, including buy-in (community, agency, home visitor), staff training, supervision, fidelity, funding, and payment structures.
Example: Program leadership; Developing content mastery and delivery skills for home visitors, recruitment and retention of home visitors, training, and supervision; Fidelity assessment
Home visiting content elements
The content (i.e. the “what”) that is conveyed by home visitors to their clients during service delivery.
Example: Home visitors engaging in information sharing for their clients to cover a broad range of topics (e.g. child development, pregnancy, labor and delivery, breastfeeding)
Home visiting process/ delivery elements
The strategies and techniques home visitors use during service delivery with their clients.
Example: Home visitor and client role play, skills practice, test taking, recitation, videos and discussion
Home visitor personal characteristics
Home visitor characteristics that may contribute to improvement in client outcomes but that aren’t typically specified in a model’s theoretical/ conceptual framework or their content. These are aspects of the home visitor that are not an explicit part of the model.
Example: Home visitor flexibility and adaptability, cultural humility, maturity, patience

---

The following elements fall under the macro element called **information sharing (by home visitor to client).** Please select which broad category each falls into. Check all that apply.

Information sharing (broadly)

- ☐ Model philosophy element
- ☐ Program implementation element
- ☐ Home visiting content element
- ☐ Home visiting process/ delivery element
- ☐ Home visitor personal characteristic

Information sharing (child development)

- ☐ Model philosophy element
- ☐ Program implementation element
- ☐ Home visiting content element
- ☐ Home visiting process/ delivery element
- ☐ Home visitor personal characteristic

Information sharing (injury prevention)

- ☐ Model philosophy element
- ☐ Program implementation element
- ☐ Home visiting content element
- ☐ Home visiting process/ delivery element
- ☐ Home visitor personal characteristic

Information sharing (substance abuse/ misuse)

- ☐ Model philosophy element
- ☐ Program implementation element
- ☐ Home visiting content element
- ☐ Home visiting process/ delivery element
- ☐ Home visitor personal characteristic

Information sharing (family planning)

- ☐ Model philosophy element
- ☐ Program implementation element
- ☐ Home visiting content element
- ☐ Home visiting process/ delivery element
- ☐ Home visitor personal characteristic

Information sharing (maternal and child health)

- ☐ Model philosophy element
- ☐ Program implementation element
- ☐ Home visiting content element
- ☐ Home visiting process/ delivery element
- ☐ Home visitor personal characteristic

Information sharing (discipline and behavior management)

- ☐ Model philosophy element
- ☐ Program implementation element
- ☐ Home visiting content element
- ☐ Home visiting process/ delivery element
- ☐ Home visitor personal characteristic

Teaching about family relationships and co-parenting

- ☐ Model philosophy element
- ☐ Program implementation element
- ☐ Home visiting content element
- ☐ Home visiting process/ delivery element
- ☐ Home visitor personal characteristic

Model philosophy elements
The tenets of an EBHV that drive the other components of home visiting, including a model's theory of change and cultural lifeways.
Example: we believe parents should be a child's first and best teacher; thus, our theory of change involves home visitors teaching parents who teach their children
Program implementation elements
Strategies, techniques, structures, and processes (e.g. program design) at the model/organizational/site level that relate to ensuring successful delivery of the

EBHV, including buy-in (community, agency, home visitor), staff training, supervision, fidelity, funding, and payment structures.
Example: Program leadership; Developing content mastery and delivery skills for home visitors, recruitment and retention of home visitors, training, and supervision; Fidelity assessment
Home visiting content elements
The content (i.e. the “what”) that is conveyed by home visitors to their clients during service delivery.
Example: Home visitors engaging in information sharing for their clients to cover a broad range of topics (e.g. child development, pregnancy, labor and delivery, breastfeeding)
Home visiting process/ delivery elements
The strategies and techniques home visitors use during service delivery with their clients.
Example: Home visitor and client role play, skills practice, test taking, recitation, videos and discussion
Home visitor personal characteristics
Home visitor characteristics that may contribute to improvement in client outcomes but that aren’t typically specified in a model’s theoretical/ conceptual framework or their content. These are aspects of the home visitor that are not an explicit part of the model.
Example: Home visitor flexibility and adaptability, cultural humility, maturity, patience

---

Which broad category does **teaching goal setting skills to parents** fall into? Check all that apply.

- [ ] Model philosophy element
- [ ] Program implementation element
- [ ] Home visiting content element
- [ ] Home visiting process/ delivery element
- [ ] Home visitor personal characteristic

Model philosophy elements

The tenets of an EBHV that drive the other components of home visiting, including a model's theory of change and cultural lifeways.
Example: we believe parents should be a child's first and best teacher; thus, our theory of change involves home visitors teaching parents who teach their children

Program implementation elements
Strategies, techniques, structures, and processes (e.g. program design) at the model/organizational/site level that relate to ensuring successful delivery of the EBHV, including buy-in (community, agency, home visitor), staff training, supervision, fidelity, funding, and payment structures.
Example: Program leadership; Developing content mastery and delivery skills for home visitors, recruitment and retention of home visitors, training, and supervision; Fidelity assessment

Home visiting content elements
The content (i.e. the "what") that is conveyed by home visitors to their clients during service delivery.
Example: Home visitors engaging in information sharing for their clients to cover a broad range of topics (e.g. child development, pregnancy, labor and delivery, breastfeeding)

Home visiting process/ delivery elements
The strategies and techniques home visitors use during service delivery with their clients.
Example: Home visitor and client role play, skills practice, test taking, recitation, videos and discussion

Home visitor personal characteristics
Home visitor characteristics that may contribute to improvement in client outcomes but that aren't typically specified in a model's theoretical/ conceptual framework or their content. These are aspects of the home visitor that are not an explicit part of the model.
Example: Home visitor flexibility and adaptability, cultural humility, maturity, patience

---

The following elements fall under the macro element called **assessment.** Please select which broad category each falls into. Check all that apply.

Child assessment and screening

- [ ] Model philosophy element
- [ ] Program implementation element
- [ ] Home visiting content element
- [ ] Home visiting process/ delivery element
- [ ] Home visitor personal characteristic

Maternal risk assessment and screening

- [ ] Model philosophy element
- [ ] Program implementation element
- [ ] Home visiting content element
- [ ] Home visiting process/ delivery element
- [ ] Home visitor personal characteristic

Processing results from caregiver screenings

- [ ] Model philosophy element
- [ ] Program implementation element
- [ ] Home visiting content element
- [ ] Home visiting process/ delivery element
- [ ] Home visitor personal characteristic

Creating an action plan based on child screenings

- [ ] Model philosophy element
- [ ] Program implementation element
- [ ] Home visiting content element
- [ ] Home visiting process/ delivery element
- [ ] Home visitor personal characteristic

Processing results from caregiver screenings

- [ ] Model philosophy element
- [ ] Program implementation element
- [ ] Home visiting content element
- [ ] Home visiting process/ delivery element
- [ ] Home visitor personal characteristic

Reflect on strategies to support results from caregiver screenings

- [ ] Model philosophy element
- [ ] Program implementation element
- [ ] Home visiting content element
- [ ] Home visiting process/ delivery element
- [ ] Home visitor personal characteristic

Model philosophy elements
The tenets of an EBHV that drive the other components of home visiting, including a model's theory of change and cultural lifeways.
Example: we believe parents should be a child's first and best teacher; thus, our theory of change involves home visitors teaching parents who teach their children
Program implementation elements
Strategies, techniques, structures, and processes (e.g. program design) at the model/organizational/site level that relate to ensuring successful delivery of the EBHV, including buy-in (community, agency, home visitor), staff training, supervision, fidelity, funding, and payment structures.
Example: Program leadership; Developing content mastery and delivery skills for home visitors, recruitment and retention of home visitors, training, and supervision; Fidelity assessment
Home visiting content elements
The content (i.e. the "what") that is conveyed by home visitors to their clients during service delivery.

Example: Home visitors engaging in information sharing for their clients to cover a broad range of topics (e.g. child development, pregnancy, labor and delivery, breastfeeding)

Home visiting process/ delivery elements

The strategies and techniques home visitors use during service delivery with their clients.

Example: Home visitor and client role play, skills practice, test taking, recitation, videos and discussion

Home visitor personal characteristics

Home visitor characteristics that may contribute to improvement in client outcomes but that aren't typically specified in a model's theoretical/ conceptual framework or their content. These are aspects of the home visitor that are not an explicit part of the model.

Example: Home visitor flexibility and adaptability, cultural humility, maturity, patience

---

The following elements fall under the macro element called **staffing/ supervision.** Please select which broad category each falls into. Check all that apply.

Reflective supervision

- ☐ Model philosophy element
- ☐ Program implementation element
- ☐ Home visiting content element
- ☐ Home visiting process/ delivery element
- ☐ Home visitor personal characteristic

Professional development

- ☐ Model philosophy element
- ☐ Program implementation element
- ☐ Home visiting content element
- ☐ Home visiting process/ delivery element

- [ ] Home visitor personal characteristic

Proper workloads of staff/supervisors

- [ ] Model philosophy element
- [ ] Program implementation element
- [ ] Home visiting content element
- [ ] Home visiting process/ delivery element
- [ ] Home visitor personal characteristic

Model philosophy elements
The tenets of an EBHV that drive the other components of home visiting, including a model's theory of change and cultural lifeways.
Example: we believe parents should be a child's first and best teacher; thus, our theory of change involves home visitors teaching parents who teach their children
Program implementation elements
Strategies, techniques, structures, and processes (e.g. program design) at the model/organizational/site level that relate to ensuring successful delivery of the EBHV, including buy-in (community, agency, home visitor), staff training, supervision, fidelity, funding, and payment structures.
Example: Program leadership; Developing content mastery and delivery skills for home visitors, recruitment and retention of home visitors, training, and supervision; Fidelity assessment
Home visiting content elements
The content (i.e. the "what") that is conveyed by home visitors to their clients during service delivery.
Example: Home visitors engaging in information sharing for their clients to cover a broad range of topics (e.g. child development, pregnancy, labor and delivery, breastfeeding)
Home visiting process/ delivery elements
The strategies and techniques home visitors use during service delivery with their clients.

Example: Home visitor and client role play, skills practice, test taking, recitation, videos and discussion

Home visitor personal characteristics

Home visitor characteristics that may contribute to improvement in client outcomes but that aren't typically specified in a model's theoretical/ conceptual framework or their content. These are aspects of the home visitor that are not an explicit part of the model.

Example: Home visitor flexibility and adaptability, cultural humility, maturity, patience

---

The following elements fall under the macro element called **staff characteristics and ability.** Please select which broad category each falls into. Check all that apply.

Staff selection

- ☐ Model philosophy element
- ☐ Program implementation element
- ☐ Home visiting content element
- ☐ Home visiting process/ delivery element
- ☐ Home visitor personal characteristic

Assertiveness and confidence of the provider

- ☐ Model philosophy element
- ☐ Program implementation element
- ☐ Home visiting content element
- ☐ Home visiting process/ delivery element
- ☐ Home visitor personal characteristic

Home visitor wisdom of the ages

- ☐ Model philosophy element

- [ ] Program implementation element
- [ ] Home visiting content element
- [ ] Home visiting process/ delivery element
- [ ] Home visitor personal characteristic

Home visitor flexibility/ adaptability

- [ ] Model philosophy element
- [ ] Program implementation element
- [ ] Home visiting content element
- [ ] Home visiting process/ delivery element
- [ ] Home visitor personal characteristic

Home visitor sense of humor

- [ ] Model philosophy element
- [ ] Program implementation element
- [ ] Home visiting content element
- [ ] Home visiting process/ delivery element
- [ ] Home visitor personal characteristic

Reliable home visitor

- [ ] Model philosophy element
- [ ] Program implementation element
- [ ] Home visiting content element
- [ ] Home visiting process/ delivery element
- [ ] Home visitor personal characteristic

Model philosophy elements
The tenets of an EBHV that drive the other components of home visiting, including a model’s theory of change and cultural lifeways.

Example: we believe parents should be a child's first and best teacher; thus, our theory of change involves home visitors teaching parents who teach their children

Program implementation elements

Strategies, techniques, structures, and processes (e.g. program design) at the model/organizational/site level that relate to ensuring successful delivery of the EBHV, including buy-in (community, agency, home visitor), staff training, supervision, fidelity, funding, and payment structures.

Example: Program leadership; Developing content mastery and delivery skills for home visitors, recruitment and retention of home visitors, training, and supervision; Fidelity assessment

Home visiting content elements

The content (i.e. the “what”) that is conveyed by home visitors to their clients during service delivery.

Example: Home visitors engaging in information sharing for their clients to cover a broad range of topics (e.g. child development, pregnancy, labor and delivery, breastfeeding)

Home visiting process/ delivery elements

The strategies and techniques home visitors use during service delivery with their clients.

Example: Home visitor and client role play, skills practice, test taking, recitation, videos and discussion

Home visitor personal characteristics

Home visitor characteristics that may contribute to improvement in client outcomes but that aren’t typically specified in a model’s theoretical/ conceptual framework or their content. These are aspects of the home visitor that are not an explicit part of the model.

Example: Home visitor flexibility and adaptability, cultural humility, maturity, patience

---

The following elements fall under the macro element called **staff skills needed.** Please select which broad category each falls into. Check all that apply.

Motivational interviewing

- [ ] Model philosophy element
- [ ] Program implementation element
- [ ] Home visiting content element
- [ ] Home visiting process/ delivery element
- [ ] Home visitor personal characteristic

Active listening

- [ ] Model philosophy element
- [ ] Program implementation element
- [ ] Home visiting content element
- [ ] Home visiting process/ delivery element
- [ ] Home visitor personal characteristic

Home visitor trauma-informed care

- [ ] Model philosophy element
- [ ] Program implementation element
- [ ] Home visiting content element
- [ ] Home visiting process/ delivery element
- [ ] Home visitor personal characteristic

Home visitor infant mental health practice

- [ ] Model philosophy element
- [ ] Program implementation element
- [ ] Home visiting content element
- [ ] Home visiting process/ delivery element
- [ ] Home visitor personal characteristic

Relationship building

- [ ] Model philosophy element
- [ ] Program implementation element
- [ ] Home visiting content element
- [ ] Home visiting process/ delivery element
- [ ] Home visitor personal characteristic

Responsiveness and sensitivity

- [ ] Model philosophy element
- [ ] Program implementation element
- [ ] Home visiting content element
- [ ] Home visiting process/ delivery element
- [ ] Home visitor personal characteristic

Cultural humility

- [ ] Model philosophy element
- [ ] Program implementation element
- [ ] Home visiting content element
- [ ] Home visiting process/ delivery element
- [ ] Home visitor personal characteristic

Empathetic communication

- [ ] Model philosophy element
- [ ] Program implementation element
- [ ] Home visiting content element
- [ ] Home visiting process/ delivery element
- [ ] Home visitor personal characteristic

Home visitor discipline regarding boundaries and limits of their role

- [ ] Model philosophy element

- [ ] Program implementation element
- [ ] Home visiting content element
- [ ] Home visiting process/ delivery element
- [ ] Home visitor personal characteristic

Reflective practice

- [ ] Model philosophy element
- [ ] Program implementation element
- [ ] Home visiting content element
- [ ] Home visiting process/ delivery element
- [ ] Home visitor personal characteristic

Model philosophy elements
The tenets of an EBHV that drive the other components of home visiting, including a model's theory of change and cultural lifeways.
Example: we believe parents should be a child's first and best teacher; thus, our theory of change involves home visitors teaching parents who teach their children
Program implementation elements
Strategies, techniques, structures, and processes (e.g. program design) at the model/organizational/site level that relate to ensuring successful delivery of the EBHV, including buy-in (community, agency, home visitor), staff training, supervision, fidelity, funding, and payment structures.
Example: Program leadership; Developing content mastery and delivery skills for home visitors, recruitment and retention of home visitors, training, and supervision; Fidelity assessment
Home visiting content elements
The content (i.e. the "what") that is conveyed by home visitors to their clients during service delivery.
Example: Home visitors engaging in information sharing for their clients to cover a broad range of topics (e.g. child development, pregnancy, labor and delivery, breastfeeding)
Home visiting process/ delivery elements

The strategies and techniques home visitors use during service delivery with their clients.
Example: Home visitor and client role play, skills practice, test taking, recitation, videos and discussion

Home visitor personal characteristics

Home visitor characteristics that may contribute to improvement in client outcomes but that aren't typically specified in a model's theoretical/ conceptual framework or their content. These are aspects of the home visitor that are not an explicit part of the model.
Example: Home visitor flexibility and adaptability, cultural humility, maturity, patience

---

Which broad category does **role play/ coaching** fall into? Check all that apply.

- ☐ Model philosophy element
- ☐ Program implementation element
- ☐ Home visiting content element
- ☐ Home visiting process/ delivery element
- ☐ Home visitor personal characteristic

Which broad category does **teaching problem solving skills to parents** fall into? Check all that apply.

- ☐ Model philosophy element
- ☐ Program implementation element
- ☐ Home visiting content element
- ☐ Home visiting process/ delivery element
- ☐ Home visitor personal characteristic

Which broad category does **home visitor content mastery** fall into? Check all that apply.

- ☐ Model philosophy element
- ☐ Program implementation element

- ☐ Home visiting content element
- ☐ Home visiting process/ delivery element
- ☐ Home visitor personal characteristic

Which broad category does **"parent voice movement"/ parent's central role in decision making** fall into? Check all that apply.

- ☐ Model philosophy element
- ☐ Program implementation element
- ☐ Home visiting content element
- ☐ Home visiting process/ delivery element
- ☐ Home visitor personal characteristic

Which broad category does **home visitor providing informal social support for families** fall into? Check all that apply.

- ☐ Model philosophy element
- ☐ Program implementation element
- ☐ Home visiting content element
- ☐ Home visiting process/ delivery element
- ☐ Home visitor personal characteristic

Model philosophy elements
The tenets of an EBHV that drive the other components of home visiting, including a model's theory of change and cultural lifeways.
Example: we believe parents should be a child's first and best teacher; thus, our theory of change involves home visitors teaching parents who teach their children
Program implementation elements
Strategies, techniques, structures, and processes (e.g. program design) at the model/organizational/site level that relate to ensuring successful delivery of the EBHV, including buy-in (community, agency, home visitor), staff training, supervision, fidelity, funding, and payment structures.

Example: Program leadership; Developing content mastery and delivery skills for home visitors, recruitment and retention of home visitors, training, and supervision; Fidelity assessment

Home visiting content elements

The content (i.e. the “what”) that is conveyed by home visitors to their clients during service delivery.

Example: Home visitors engaging in information sharing for their clients to cover a broad range of topics (e.g. child development, pregnancy, labor and delivery, breastfeeding)

Home visiting process/ delivery elements

The strategies and techniques home visitors use during service delivery with their clients.

Example: Home visitor and client role play, skills practice, test taking, recitation, videos and discussion

Home visitor personal characteristics

Home visitor characteristics that may contribute to improvement in client outcomes but that aren’t typically specified in a model’s theoretical/ conceptual framework or their content. These are aspects of the home visitor that are not an explicit part of the model.

Example: Home visitor flexibility and adaptability, cultural humility, maturity, patience

---

Which broad category does **home visitor modeling of desired behaviors** fall into? Check all that apply.

- [ ] Model philosophy element
- [ ] Program implementation element
- [ ] Home visiting content element
- [ ] Home visiting process/ delivery element
- [ ] Home visitor personal characteristic

Which broad category does **teaching relaxation/ self-regulation skills to parents** fall into? Check all that apply.

- [ ] Model philosophy element

- [ ] Program implementation element
- [ ] Home visiting content element
- [ ] Home visiting process/ delivery element
- [ ] Home visitor personal characteristic

Which broad category does **culture of quality for implementing program** fall into? Check all that apply.

- [ ] Model philosophy element
- [ ] Program implementation element
- [ ] Home visiting content element
- [ ] Home visiting process/ delivery element
- [ ] Home visitor personal characteristic

Which broad category does **home visitor observation of parent-child interactions** fall into? Check all that apply.

- [ ] Model philosophy element
- [ ] Program implementation element
- [ ] Home visiting content element
- [ ] Home visiting process/ delivery element
- [ ] Home visitor personal characteristic

Which broad category does **organization/ program collaboration and outreach across the community** fall into? Check all that apply.

- [ ] Model philosophy element
- [ ] Program implementation element
- [ ] Home visiting content element
- [ ] Home visiting process/ delivery element
- [ ] Home visitor personal characteristic

Model philosophy elements
The tenets of an EBHV that drive the other components of home visiting, including a model's theory of change and cultural lifeways.
Example: we believe parents should be a child's first and best teacher; thus, our theory of change involves home visitors teaching parents who teach their children
Program implementation elements
Strategies, techniques, structures, and processes (e.g. program design) at the model/organizational/site level that relate to ensuring successful delivery of the EBHV, including buy-in (community, agency, home visitor), staff training, supervision, fidelity, funding, and payment structures.
Example: Program leadership; Developing content mastery and delivery skills for home visitors, recruitment and retention of home visitors, training, and supervision; Fidelity assessment
Home visiting content elements
The content (i.e. the "what") that is conveyed by home visitors to their clients during service delivery.
Example: Home visitors engaging in information sharing for their clients to cover a broad range of topics (e.g. child development, pregnancy, labor and delivery, breastfeeding)
Home visiting process/ delivery elements
The strategies and techniques home visitors use during service delivery with their clients.
Example: Home visitor and client role play, skills practice, test taking, recitation, videos and discussion
Home visitor personal characteristics
Home visitor characteristics that may contribute to improvement in client outcomes but that aren't typically specified in a model's theoretical/ conceptual framework or their content. These are aspects of the home visitor that are not an explicit part of the model.
Example: Home visitor flexibility and adaptability, cultural humility, maturity, patience

---

Which broad category does **model is based on a parenting framework** fall into? Check all that apply.

- [ ] Model philosophy element
- [ ] Program implementation element
- [ ] Home visiting content element
- [ ] Home visiting process/ delivery element
- [ ] Home visitor personal characteristic

Which broad category does **teaching coping skills to parents** fall into? Check all that apply.

- [ ] Model philosophy element
- [ ] Program implementation element
- [ ] Home visiting content element
- [ ] Home visiting process/ delivery element
- [ ] Home visitor personal characteristic

Which broad category does **program is data driven** fall into? Check all that apply.

- [ ] Model philosophy element
- [ ] Program implementation element
- [ ] Home visiting content element
- [ ] Home visiting process/ delivery element
- [ ] Home visitor personal characteristic

Which broad category does **recruitment of/ outreach to parents** fall into? Check all that apply.

- [ ] Model philosophy element
- [ ] Program implementation element
- [ ] Home visiting content element
- [ ] Home visiting process/ delivery element
- [ ] Home visitor personal characteristic

Which broad category does **appropriate frequency of visits by the home visitor** fall into? Check all that apply.

- [ ] Model philosophy element
- [ ] Program implementation element
- [ ] Home visiting content element
- [ ] Home visiting process/ delivery element
- [ ] Home visitor personal characteristic

Model philosophy elements
The tenets of an EBHV that drive the other components of home visiting, including a model's theory of change and cultural lifeways.
Example: we believe parents should be a child's first and best teacher; thus, our theory of change involves home visitors teaching parents who teach their children
Program implementation elements
Strategies, techniques, structures, and processes (e.g. program design) at the model/organizational/site level that relate to ensuring successful delivery of the EBHV, including buy-in (community, agency, home visitor), staff training, supervision, fidelity, funding, and payment structures.
Example: Program leadership; Developing content mastery and delivery skills for home visitors, recruitment and retention of home visitors, training, and supervision; Fidelity assessment
Home visiting content elements
The content (i.e. the "what") that is conveyed by home visitors to their clients during service delivery.
Example: Home visitors engaging in information sharing for their clients to cover a broad range of topics (e.g. child development, pregnancy, labor and delivery, breastfeeding)
Home visiting process/ delivery elements
The strategies and techniques home visitors use during service delivery with their clients.
Example: Home visitor and client role play, skills practice, test taking, recitation, videos and discussion

Home visitor personal characteristics
Home visitor characteristics that may contribute to improvement in client outcomes but that aren't typically specified in a model's theoretical/ conceptual framework or their content. These are aspects of the home visitor that are not an explicit part of the model.
Example: Home visitor flexibility and adaptability, cultural humility, maturity, patience

---

Which broad category does **home visitor asking client reflective questions** fall into? Check all that apply.

- ☐ Model philosophy element
- ☐ Program implementation element
- ☐ Home visiting content element
- ☐ Home visiting process/ delivery element
- ☐ Home visitor personal characteristic

Which broad category does **both parent and child attend** fall into? Check all that apply.

- ☐ Model philosophy element
- ☐ Program implementation element
- ☐ Home visiting content element
- ☐ Home visiting process/ delivery element
- ☐ Home visitor personal characteristic

Which category does the following element fall into: **home visitor matching content to risk factors inherent in the population (not specific client need) - preventing substance abuse; preventing short birth spacing?** Check all that apply.

- ☐ Model philosophy element
- ☐ Program implementation element
- ☐ Home visiting content element

- [ ] Home visiting process/ delivery element
- [ ] Home visitor personal characteristic

Which broad category does **adequate and stable funding for implementing the EBHV program** fall into? Check all that apply.

- [ ] Model philosophy element
- [ ] Program implementation element
- [ ] Home visiting content element
- [ ] Home visiting process/ delivery element
- [ ] Home visitor personal characteristic

Which broad category does **state coordinated systems** fall into? Check all that apply.

- [ ] Model philosophy element
- [ ] Program implementation element
- [ ] Home visiting content element
- [ ] Home visiting process/ delivery element
- [ ] Home visitor personal characteristic

Model philosophy elements
The tenets of an EBHV that drive the other components of home visiting, including a model’s theory of change and cultural lifeways.
Example: we believe parents should be a child's first and best teacher; thus, our theory of change involves home visitors teaching parents who teach their children
Program implementation elements
Strategies, techniques, structures, and processes (e.g. program design) at the model/organizational/site level that relate to ensuring successful delivery of the EBHV, including buy-in (community, agency, home visitor), staff training, supervision, fidelity, funding, and payment structures.

Example: Program leadership; Developing content mastery and delivery skills for home visitors, recruitment and retention of home visitors, training, and supervision; Fidelity assessment

Home visiting content elements

The content (i.e. the “what”) that is conveyed by home visitors to their clients during service delivery.

Example: Home visitors engaging in information sharing for their clients to cover a broad range of topics (e.g. child development, pregnancy, labor and delivery, breastfeeding)

Home visiting process/ delivery elements

The strategies and techniques home visitors use during service delivery with their clients.

Example: Home visitor and client role play, skills practice, test taking, recitation, videos and discussion

Home visitor personal characteristics

Home visitor characteristics that may contribute to improvement in client outcomes but that aren’t typically specified in a model’s theoretical/ conceptual framework or their content. These are aspects of the home visitor that are not an explicit part of the model.

Example: Home visitor flexibility and adaptability, cultural humility, maturity, patience

---

Which broad category does the following element fall into: **home visitor participating in family team meeting with other professionals?** Check all that apply.

- ☐ Model philosophy element
- ☐ Program implementation element
- ☐ Home visiting content element
- ☐ Home visiting process/ delivery element
- ☐ Home visitor personal characteristic

Which broad category does the following element fall into: **home visitor practice difficult conversations that you need to have with participants**

with co-workers or supervisors? Check all that apply.

- ☐ Model philosophy element
- ☐ Program implementation element
- ☐ Home visiting content element
- ☐ Home visiting process/ delivery element
- ☐ Home visitor personal characteristic

Which broad category does **informed policy making with family voice** fall into? Check all that apply.

- ☐ Model philosophy element
- ☐ Program implementation element
- ☐ Home visiting content element
- ☐ Home visiting process/ delivery element
- ☐ Home visitor personal characteristic

Model philosophy elements
The tenets of an EBHV that drive the other components of home visiting, including a model's theory of change and cultural lifeways.
Example: we believe parents should be a child's first and best teacher; thus, our theory of change involves home visitors teaching parents who teach their children
Program implementation elements
Strategies, techniques, structures, and processes (e.g. program design) at the model/organizational/site level that relate to ensuring successful delivery of the EBHV, including buy-in (community, agency, home visitor), staff training, supervision, fidelity, funding, and payment structures.
Example: Program leadership; Developing content mastery and delivery skills for home visitors, recruitment and retention of home visitors, training, and supervision; Fidelity assessment
Home visiting content elements
The content (i.e. the "what") that is conveyed by home visitors to their clients during service delivery.

Example: Home visitors engaging in information sharing for their clients to cover a broad range of topics (e.g. child development, pregnancy, labor and delivery, breastfeeding)

Home visiting process/ delivery elements

The strategies and techniques home visitors use during service delivery with their clients.

Example: Home visitor and client role play, skills practice, test taking, recitation, videos and discussion

Home visitor personal characteristics

Home visitor characteristics that may contribute to improvement in client outcomes but that aren't typically specified in a model's theoretical/ conceptual framework or their content. These are aspects of the home visitor that are not an explicit part of the model.

Example: Home visitor flexibility and adaptability, cultural humility, maturity, patience

---

The following elements were identified as specific to **tribal home visiting.** Please select which broad category each falls into. Check all that apply.

Culturally informed knowledge of the home visitor

- ☐ Model philosophy element
- ☐ Program implementation element
- ☐ Home visiting content element
- ☐ Home visiting process/ delivery element
- ☐ Home visitor personal characteristic

Home visitor fosters cultural identity of clients

- ☐ Model philosophy element
- ☐ Program implementation element
- ☐ Home visiting content element
- ☐ Home visiting process/ delivery element
- ☐ Home visitor personal characteristic

Home visitor cultural understanding

- ☐ Model philosophy element
- ☐ Program implementation element
- ☐ Home visiting content element
- ☐ Home visiting process/ delivery element
- ☐ Home visitor personal characteristic

Home visitor showing cultural respect

- ☐ Model philosophy element
- ☐ Program implementation element
- ☐ Home visiting content element
- ☐ Home visiting process/ delivery element
- ☐ Home visitor personal characteristic

Home visitor shares resources in their native language

- ☐ Model philosophy element
- ☐ Program implementation element
- ☐ Home visiting content element
- ☐ Home visiting process/ delivery element
- ☐ Home visitor personal characteristic

Multi-generational participation of client's household

- ☐ Model philosophy element
- ☐ Program implementation element
- ☐ Home visiting content element
- ☐ Home visiting process/ delivery element
- ☐ Home visitor personal characteristic

Program establishing relationships with Tribal leadership so that tribal leaders understand the importance of home visiting

- ☐ Model philosophy element
- ☐ Program implementation element
- ☐ Home visiting content element
- ☐ Home visiting process/ delivery element
- ☐ Home visitor personal characteristic

Program connections and partnerships with Tribal services

- ☐ Model philosophy element
- ☐ Program implementation element
- ☐ Home visiting content element
- ☐ Home visiting process/ delivery element
- ☐ Home visitor personal characteristic

Meetings with clients in or outside the home

- ☐ Model philosophy element
- ☐ Program implementation element
- ☐ Home visiting content element
- ☐ Home visiting process/ delivery element
- ☐ Home visitor personal characteristic

Implementing program allows for multi-family/ group visits

- ☐ Model philosophy element
- ☐ Program implementation element
- ☐ Home visiting content element
- ☐ Home visiting process/ delivery element
- ☐ Home visitor personal characteristic

Culturally responsive approach with all staff training, strategies, materials

- [ ] Model philosophy element
- [ ] Program implementation element
- [ ] Home visiting content element
- [ ] Home visiting process/ delivery element
- [ ] Home visitor personal characteristic

Please make any additional comments here. You can share feedback about the broad categories generally or specific elements that were previously generated by panel members.

Are there any additional elements you would like to add?

Name of the person who filled out this questionnaire

Thank you for taking the time to complete Questionnaire #2 for the EBHV standard practice elements project. We will combine data and follow-up to schedule the next video conference.

If you have any questions or concerns about this process, please don't hesitate to reach out to the Johns Hopkins team: Kayla, Allie, and/or Emily.

Powered by Qualtrics
