# Supplementary material for: Expert-generated standard practice elements for evidence-based home visiting programs using a Delphi process
Source: PLoS One. 2022 Oct 17;17(10):e0275981. doi: 10.1371/journal.pone.0275981 (PMC9576067; doi:10.1371/journal.pone.0275981)
Supplement: S3 File — (PDF) [file pone.0275981.s003.pdf]

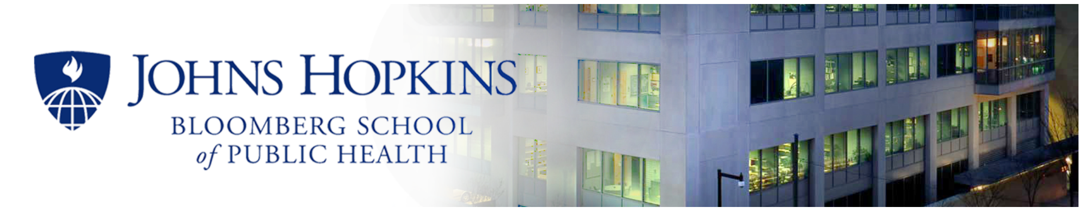


## Default Question Block

---

The purpose of this questionnaire is to finalize action items from our last video conference, held on Thursday, October 1. It should take no more than 5 minutes to complete.

Thank you for your continued participation as an expert panel member.

---

In this first section, we would like to determine whether the strategies and techniques used in a **trauma-informed care approach** are also EBHV standard practice elements and which category of elements they belong to.

Please note that we pulled these strategies and techniques from the following resource specific to home visiting: [https://www.jbassoc.com/wp-content/uploads/2020/05/Implementing-Trauma-Informed-Approaches-Home-Visiting.pdf](https://www.jbassoc.com/wp-content/uploads/2020/05/Implementing-Trauma-Informed-Approaches-Home-Visiting.pdf)

---

**Program trains staff on the prevalence, causes, and consequences of trauma:** Is this an EBHV standard practice element?

- ○ Yes
- ○ No

---

**Program trains staff on the prevalence, causes, and consequences of trauma:** Which practice element category does this belong in? [Check all that apply.]

- [ ] Home visiting content element
- [ ] Home visiting process/ delivery element
- [ ] Home visitor personal characteristic
- [ ] Program implementation element
- [ ] Model philosophy element

---

**Home visitor administration of client trauma screening:** Is this an EBHV standard practice element?

- ○ Yes
- ○ No

---

**Home visitor administration of client trauma screening:** Which practice element category does this belong in? [Check all that apply.]

- [ ] Home visiting content element
- [ ] Home visiting process/ delivery element
- [ ] Home visitor personal characteristic
- [ ] Program implementation element
- [ ] Model philosophy element

---

**Program strengthening of service coordination**: Is this an EBHV standard practice element?

- ○ Yes
- ○ No

---

**Program strengthening service coordination**: Which practice element category does this belong in? [Check all that apply.]

- [ ] Home visiting content element
- [ ] Home visiting process/ delivery element
- [ ] Home visitor personal characteristic

☐ Program implementation element

☐ Model philosophy element

---

During the video conference, we had also discussed wanting to determine whether the strategies and techniques used in a **infant mental health practice** are also EBHV standard practice elements. We reviewed the following resource on infant mental health practice specific to home visiting, and compared it with our list of existing standard practice elements: https://www.zerotothree.org/resources/142-infant-mental-health-home-visiting-strategies

We felt that these strategies and techniques were already fully represented in our existing list. Thus, our question for you on this survey is, **Can you identify any strategies and techniques in infant mental health practice that we have missed and are important to include as EBHV standard practice elements?** [Refer to this file, listing all current elements: Coding categorized updated 10 07 2020 ]

○ Yes

○ No

---

Please list any additional **infant mental health practice** standard practice elements that you feel are important to include in our taxonomy.

---

Each of the elements listed on this page was identified by only one panel member on questionnaire #1 (free listing of standard practice elements). We would like further input from each of you on whether to include it in the final list of EBHV standard practice elements.

**As a reminder, we define standard practice elements as the techniques and strategies used in early childhood home visiting as part of a larger intervention.**

**Informed policy making with family voice** (program implementation, model philosophy): Is this definitely a standard practice element in home visiting?

- Yes
- No

**Staff selection** (program implementation): Is this definitely a standard practice element in home visiting

- Yes
- No

**Assertiveness and confidence of the provider** (home visitor personal characteristic): Is this definitely a standard practice element in home visiting?

- Yes
- No

**Home visitor wisdom of the ages** (home visitor personal characteristic): Is this definitely a standard practice element in home visiting?

- Yes
- No

**Home visitor discipline regarding boundaries and limits of their role** (home visiting process/delivery, home visitor personal characteristic): Is this definitely a standard practice element in home visiting?

- Yes
- No

**Reflective practice** (home visiting process/delivery): Is this a standard practice element in home visiting?

- Yes
- No

**Program is data driven** (program implementation, model philosophy): Is this definitely a standard practice element in home visiting?

- Yes
- No

**Home visitor asking client reflective questions** (home visiting process/delivery): Is this definitely a standard practice element in home visiting?

- Yes
- No

**Home visitor matching content to risk factors inherent in the population (not specific client need)-preventing substance abuse, preventing short birth spacing** (home visiting content): Is this definitely a standard practice element in home visiting?

- Yes
- No

**Adequate and stable funding for implementing the EBHV program** (program implementation): Is this definitely a standard practice element in home visiting?

- Yes
- No

**State coordinated systems** (program implementation): Is this definitely a standard practice element in home visiting?

- Yes
- No

---

**Home visitor participating in family team meeting with other professionals** (home visiting process/ delivery, program implementation): Is this definitely a standard practice element in home visiting?

- Yes
- No

---

**Home visitor practice difficult conversations that you need to have with participants with co-workers or supervisors** (program implementation): Is this definitely a standard practice element in home visiting?

- Yes
- No

---

**Program establishing relationships with tribal leadership so that tribal leaders understand the importance of home visiting** (program implementation): Is this definitely a standard practice element in home visiting?

- Yes
- No

---

**Culturally responsive approach with all staff training, strategies, and materials** (program implementation): Is this definitely a standard practice element in home visiting?

- Yes
- No

Name of person who filled out this questionnaire

Thank you for taking the time to complete this questionnaire. We will be back in touch shortly.

Powered by Qualtrics
