# Supplementary material for: Expert-generated standard practice elements for evidence-based home visiting programs using a Delphi process
Source: PLoS One. 2022 Oct 17;17(10):e0275981. doi: 10.1371/journal.pone.0275981 (PMC9576067; doi:10.1371/journal.pone.0275981)
Supplement: S4 File — (PDF) [file pone.0275981.s004.pdf]

JOHNS HOPKINS
BLOOMBERG SCHOOL
of PUBLIC HEALTH

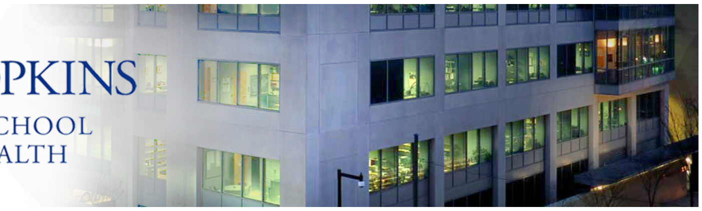

Goals and Planning

Please drag the element to the behavior change technique if it does the same thing

Items	
Providing clients with linkage to services	**Goal setting (behavior)**
Information sharing (by home visitor to client)	
Teaching goal setting skills to parents	
child assessment and screening	
maternal risk assessment and screening	**Problem Solving**
processing results from caregiver screenings	
creating an action plan based on child screenings	
processing results from child screenings	**Goal setting (outcome)**
reflect on strategies to support results from caregivers screenings	
reflective supervision	
professional development	
proper workloads of staff/supervisors	

Criteria for staff selection are appropriate for population served

home visitor flexibility/ adaptability

home visitor sense of humor

reliable home visitor

motivational interviewing

active listening

relationship building

responsivness and sensitivity

home visitor demonstrates cultural humility

empathetic communication

home visitor discipline regarding boundaries and limits of their role

Program trains staff on the prevalence, causes, and consequences of trauma

Program strengthening of service coordination

Role play/ coaching

Teaching problem solving skills to parents

Home visitor content mastery

Home visitor providing informal social support for families

Home visitor modeling of desired behaviors

Action planning

Review behavior goal(s)

Discrepancy between current behavior and goal

Review outcome goal(s)

Behavioral contract

Teaching relaxation/ self-regulation skills to parents

Culture of quality for implementing program

Home visitor observation of parent-child interactions

Organization/ program collaboration and outreach across the community

Model is based on a parenting framework

Teaching coping skills to parents

Program is data driven

recruitment of/outreach to parents

appropriate frequency of visits by the home visitor

Home visitor adaptability with respect to setting and participation

Culturally informed knowledge of the home visitor

Home visitor understands, affirms, and respects cultural identity of clients

Home visitor facilitates client connection to cultural and/or spiritual resources

Home visitor shares resources in client's Native language

Culturally attuned and responsive approach with all staff training, strategies, materials

Commitment

Feedback and monitoring

Please drag the element to the behavior change technique if it does the same thing

Items

- Providing clients with linkage to services
- Information sharing (by home visitor to client)
- Teaching goal setting skills to parents
- child assessment and screening
- maternal risk assessment and screening
- processing results from caregiver screenings
- creating an action plan based on child screenings
- processing results from child screenings
- reflect on strategies to support results from caregivers screenings
- reflective supervision
- professional development
- proper workloads of staff/supervisors
- criteria for staff selection are appropriate for population served
- home visitor flexibility/ adaptability
- home visitor sense of humor
- reliable home visitor

Monitoring of behavior by others without feedback

Feedback on behavior

Self-monitoring of behavior

Self-monitoring of outcome(s) of behavior

motivational interviewing

active listening

relationship building

responsivness and sensitivity

Home visitor demonstrates cultural humility

empathetic communication

home visitor discipline regarding boundaries and limits of their role

Program trains staff on the prevalence, causes, and consequences of trauma

Program strengthening of service coordination

Role play/ coaching

Teaching problem solving skills to parents

Home visitor content mastery

Home visitor providing informal social support for families

Home visitor modeling of desired behaviors

Teaching relaxation/ self-regulation skills to parents

Culture of quality for implementing program

Home visitor observation of parent-child interactions

Organization/ program collaboration and

Monitoring of outcomes of behavior without feedback

Biofeedback

Feedback on outcome(s) of behavior

outreach across the community

Model is based on a parenting framework

Teaching coping skills to parents

Program is data driven

recruitment of/outreach to parents

appropriate frequency of visits by the home visitor

Home visitor adaptability with respect to setting and participation

Culturally informed knowledge of the home visitor

Home visitor understands, affirms, and respects cultural identity of clients

Home visitor facilitates client connection to cultural and/or spiritual resources

Home visitor shares resources in client's Native language

Culturally attuned and responsive approach with all staff training, strategies, materials

---

Social Support

Please drag the element to the behavior change technique if it does the same thing

Items

Social support (unspecified)

Providing clients with linkage to services

Information sharing (by home visitor to client)

Teaching goal setting skills to parents

child assessment and screening

maternal risk assessment and screening

processing results from caregiver screenings

creating an action plan based on child screenings

processing results from child screenings

reflect on strategies to support results from caregivers screenings

reflective supervision

professional development

proper workloads of staff/supervisors

criteria for staff selection are appropriate for population served

home visitor flexibility/ adaptability

home visitor sense of humor

reliable home visitor

motivational interviewing

active listening

relationship building

responsivness and sensitivity

Social support (practical)

Social support (emotional)

Home visitor demonstrates cultural humility

empathetic communication

home visitor discipline regarding boundaries and limits of their role

Program trains staff on the prevalence, causes, and consequences of trauma

Program strengthening of service coordination

Role play/ coaching

Teaching problem solving skills to parents

Home visitor content mastery

Home visitor providing informal social support for families

Home visitor modeling of desired behaviors

Teaching relaxation/ self-regulation skills to parents

Culture of quality for implementing program

Home visitor observation of parent-child interactions

Organization/ program collaboration and outreach across the community

Model is based on a parenting framework

Teaching coping skills to parents

Program is data driven

recruitment of/outreach to parents

appropriate frequency of visits by the home visitor

Home visitor adaptability with respect to setting and participation

Culturally informed knowledge of the home visitor

Home visitor understands, affirms, and respects cultural identity of clients

Home visitor facilitates client connection to cultural and/or spiritual resources

Home visitor shares resources in client's Native language

Culturally attuned and responsive approach with all staff training, strategies, materials

Shaping Knowledge

Please drag the element to the behavior change technique if it does the same thing

Items	Instruction on how to perform the behavior
Providing clients with linkage to services	
Information sharing (by home visitor to client)	
Teaching goal setting skills to parents	
child assessment and screening	

maternal risk assessment and screening

processing results from caregiver screenings

creating an action plan based on child screenings

processing results from child screenings

reflect on strategies to support results from caregivers screenings

reflective supervision

professional development

proper workloads of staff/supervisors

criteria for staff selection are appropriate for population served

home visitor flexibility/ adaptability

home visitor sense of humor

reliable home visitor

motivational interviewing

active listening

relationship building

responsivness and sensitivity

Home visitor demonstrates cultural humility

empathetic communication

home visitor discipline regarding boundaries and limits of their role

Information about antecedents

Re-attribution

Behavioral experiments

Program trains staff on the prevalence, causes, and consequences of trauma

Program strengthening of service coordination

Role play/ coaching

Teaching problem solving skills to parents

Home visitor content mastery

Home visitor providing informal social support for families

Home visitor modeling of desired behaviors

Teaching relaxation/ self-regulation skills to parents

Culture of quality for implementing program

Home visitor observation of parent-child interactions

Organization/ program collaboration and outreach across the community

Model is based on a parenting framework

Teaching coping skills to parents

Program is data driven

recruitment of/outreach to parents

appropriate frequency of visits by the home visitor

Home visitor adaptability with respect to setting and participation

Culturally informed knowledge of the home visitor

Home visitor understands, affirms, and respects cultural identity of clients

Home visitor facilitates client connection to cultural and/or spiritual resources

Home visitor shares resources in client's Native language

Culturally attuned and responsive approach with all staff training, strategies, materials

---

Natural consequences

Please drag the element to the behavior change technique if it does the same thing

Items

Providing clients with linkage to services

Information sharing (by home visitor to client)

Teaching goal setting skills to parents

child assessment and screening

maternal risk assessment and screening

processing results from caregiver screenings

creating an action plan based on child screenings

Information about health consequences

Salience of consequences

processing results from child screenings

reflect on strategies to support results from caregivers screenings

reflective supervision

professional development

proper workloads of staff/supervisors

criteria for staff selection are appropriate for population served

home visitor flexibility/ adaptability

home visitor sense of humor

reliable home visitor

motivational interviewing

active listening

relationship building

responsivness and sensitivity

Home visitor demonstrates cultural humility

empathetic communication

home visitor discipline regarding boundaries and limits of their role

Program trains staff on the prevalence, causes, and consequences of trauma

Program strengthening of service coordination

Role play/ coaching

Information about social and environmental consequences

Monitoring of emotional consequences

Anticipated regret

Information about emotional consequences

Teaching problem solving skills to parents

Home visitor content mastery

Home visitor providing informal social support for families

Home visitor modeling of desired behaviors

Teaching relaxation/ self-regulation skills to parents

Culture of quality for implementing program

Home visitor observation of parent-child interactions

Organization/ program collaboration and outreach across the community

Model is based on a parenting framework

Teaching coping skills to parents

Program is data driven

recruitment of/outreach to parents

appropriate frequency of visits by the home visitor

Home visitor adaptability with respect to setting and participation

Culturally informed knowledge of the home visitor

Home visitor understands, affirms, and respects cultural identity of clients

Home visitor facilitates client connection to

cultural and/or spiritual resources

Home visitor shares resources in client's Native language

Culturally attuned and responsive approach with all staff training, strategies, materials

Comparison of behavior

Please drag the element to the behavior change technique if it does the same thing

Items

- Providing clients with linkage to services
- Information sharing (by home visitor to client)
- Teaching goal setting skills to parents
- child assessment and screening
- maternal risk assessment and screening
- processing results from caregiver screenings
- creating an action plan based on child screenings
- processing results from child screenings
- reflect on strategies to support results from caregivers screenings
- reflective supervision
- professional development

Demonstration of the behavior

Social comparison

Information about others' approval

proper workloads of staff/supervisors

criteria for staff selection are appropriate for population served

home visitor flexibility/ adaptability

home visitor sense of humor

reliable home visitor

motivational interviewing

active listening

relationship building

responsivness and sensitivity

Home visitor demonstrates cultural humility

empathetic communication

home visitor discipline regarding boundaries and limits of their role

Program trains staff on the prevalence, causes, and consequences of trauma

Program strengthening of service coordination

Role play/ coaching

Teaching problem solving skills to parents

Home visitor content mastery

Home visitor providing informal social support for families

Home visitor modeling of desired behaviors

Teaching relaxation/ self-regulation skills to parents

Culture of quality for implementing program

Home visitor observation of parent-child interactions

Organization/ program collaboration and outreach across the community

Model is based on a parenting framework

Teaching coping skills to parents

Program is data driven

recruitment of/outreach to parents

appropriate frequency of visits by the home visitor

Home visitor adaptability with respect to setting and participation

Culturally informed knowledge of the home visitor

Home visitor understands, affirms, and respects cultural identity of clients

Home visitor facilitates client connection to cultural and/or spiritual resources

Home visitor shares resources in client's Native language

Culturally attuned and responsive approach with

all staff training, strategies, materials

Associations

Please drag the element to the behavior change technique if it does the same thing

Items

Providing clients with linkage to services

Information sharing (by home visitor to client)

Teaching goal setting skills to parents

child assessment and screening

maternal risk assessment and screening

processing results from caregiver screenings

creating an action plan based on child screenings

processing results from child screenings

reflect on strategies to support results from caregivers screenings

reflective supervision

professional development

proper workloads of staff/supervisors

criteria for staff selection are appropriate for population served

home visitor flexibility/ adaptability

Prompts/cues

Cue signaling reward

Reduce prompts/cues

Remove access to the reward

home visitor sense of humor

reliable home visitor

motivational interviewing

active listening

relationship building

responsivness and sensitivity

Home visitor demonstrates cultural humility

empathetic communication

home visitor discipline regarding boundaries and limits of their role

Program trains staff on the prevalence, causes, and consequences of trauma

Program strengthening of service coordination

Role play/ coaching

Teaching problem solving skills to parents

Home visitor content mastery

Home visitor providing informal social support for families

Home visitor modeling of desired behaviors

Teaching relaxation/ self-regulation skills to parents

Culture of quality for implementing program

Home visitor observation of parent-child

Remove aversive stimulus

Satiation

Exposure

Associative learning

interactions

Organization/ program collaboration and outreach across the community

Model is based on a parenting framework

Teaching coping skills to parents

Program is data driven

recruitment of/outreach to parents

appropriate frequency of visits by the home visitor

Home visitor adaptability with respect to setting and participation

Culturally informed knowledge of the home visitor

Home visitor understands, affirms, and respects cultural identity of clients

Home visitor facilitates client connection to cultural and/or spiritual resources

Home visitor shares resources in client's Native language

Culturally attuned and responsive approach with all staff training, strategies, materials

---

Repetition and Substitution

Please drag the element to the behavior change technique if it does the same thing

Items	
Providing clients with linkage to services	**Behavioral practice/rehearsal**
Information sharing (by home visitor to client)	
Teaching goal setting skills to parents	
child assessment and screening	**Behavior substitution**
maternal risk assessment and screening	
processing results from caregiver screenings	
creating an action plan based on child screenings	
processing results from child screenings	**Habit formation**
reflect on strategies to support results from caregivers screenings	
reflective supervision	
professional development	
proper workloads of staff/supervisors	
criteria for staff selection are appropriate for population served	**Habit reversal**
home visitor flexibility/ adaptability	
home visitor sense of humor	
reliable home visitor	
motivational interviewing	**Overcorrection**
active listening	
relationship building	

responsivness and sensitivity

Home visitor demonstrates cultural humility

empathetic communication

home visitor discipline regarding boundaries and limits of their role

Program trains staff on the prevalence, causes, and consequences of trauma

Program strengthening of service coordination

Role play/ coaching

Teaching problem solving skills to parents

Home visitor content mastery

Home visitor providing informal social support for families

Home visitor modeling of desired behaviors

Teaching relaxation/ self-regulation skills to parents

Culture of quality for implementing program

Home visitor observation of parent-child interactions

Organization/ program collaboration and outreach across the community

Model is based on a parenting framework

Generalization of target behavior

Graded tasks

Teaching coping skills to parents

Program is data driven

recruitment of/outreach to parents

appropriate frequency of visits by the home visitor

Home visitor adaptability with respect to setting and participation

Culturally informed knowledge of the home visitor

Home visitor understands, affirms, and respects cultural identity of clients

Home visitor facilitates client connection to cultural and/or spiritual resources

Home visitor shares resources in client's Native language

Culturally attuned and responsive approach with all staff training, strategies, materials

---

Comparison of outcomes

Please drag the element to the behavior change technique if it does the same thing

Items

Providing clients with linkage to services

Information sharing (by home visitor to client)

Credible source

Teaching goal setting skills to parents

child assessment and screening

maternal risk assessment and screening

processing results from caregiver screenings

creating an action plan based on child screenings

processing results from child screenings

reflect on strategies to support results from caregivers screenings

reflective supervision

professional development

proper workloads of staff/supervisors

criteria for staff selection are appropriate for population served

home visitor flexibility/ adaptability

home visitor sense of humor

reliable home visitor

motivational interviewing

active listening

relationship building

responsivness and sensitivity

Home visitor demonstrates cultural humility

empathetic communication

Pros and cons

Comparative imagining of future outcomes

home visitor discipline regarding boundaries and limits of their role

Program trains staff on the prevalence, causes, and consequences of trauma

Program strengthening of service coordination

Role play/ coaching

Teaching problem solving skills to parents

Home visitor content mastery

Home visitor providing informal social support for families

Home visitor modeling of desired behaviors

Teaching relaxation/ self-regulation skills to parents

Culture of quality for implementing program

Home visitor observation of parent-child interactions

Organization/ program collaboration and outreach across the community

Model is based on a parenting framework

Teaching coping skills to parents

Program is data driven

recruitment of/outreach to parents

appropriate frequency of visits by the home visitor

Home visitor adaptability with respect to setting and participation

Culturally informed knowledge of the home visitor

Home visitor understands, affirms, and respects cultural identity of clients

Home visitor facilitates client connection to cultural and/or spiritual resources

Home visitor shares resources in client's Native language

Culturally attuned and responsive approach with all staff training, strategies, materials

---

Reward and threat

Please drag the element to the behavior change technique if it does the same thing

Items

Providing clients with linkage to services

Information sharing (by home visitor to client)

Teaching goal setting skills to parents

child assessment and screening

maternal risk assessment and screening

processing results from caregiver screenings

Material incentive (behavior)

Material reward (behavior)

creating an action plan based on child screenings

processing results from child screenings

reflect on strategies to support results from caregivers screenings

reflective supervision

professional development

proper workloads of staff/supervisors

criteria for staff selection are appropriate for population served

home visitor flexibility/ adaptability

home visitor sense of humor

reliable home visitor

motivational interviewing

active listening

relationship building

responsivness and sensitivity

Home visitor demonstrates cultural humility

empathetic communication

home visitor discipline regarding boundaries and limits of their role

Program trains staff on the prevalence, causes, and consequences of trauma

Program strengthening of service coordination

Non-specific reward

Social reward

Social incentive

Non-specific incentive

Self-incentive

Role play/ coaching

Teaching problem solving skills to parents

Home visitor content mastery

Home visitor providing informal social support for families

Home visitor modeling of desired behaviors

Teaching relaxation/ self-regulation skills to parents

Culture of quality for implementing program

Home visitor observation of parent-child interactions

Organization/ program collaboration and outreach across the community

Model is based on a parenting framework

Teaching coping skills to parents

Program is data driven

recruitment of/outreach to parents

appropriate frequency of visits by the home visitor

Home visitor adaptability with respect to setting and participation

Culturally informed knowledge of the home visitor

Home visitor understands, affirms, and respects cultural identity of clients

Incentive (outcome)

Self-reward

Reward (outcome)

Future punishment

Home visitor facilitates client connection to cultural and/or spiritual resources

Home visitor shares resources in client's Native language

Culturally attuned and responsive approach with all staff training, strategies, materials

---

Regulation

Please drag the element to the behavior change technique if it does the same thing

Items

Providing clients with linkage to services

Information sharing (by home visitor to client)

Teaching goal setting skills to parents

child assessment and screening

maternal risk assessment and screening

processing results from caregiver screenings

creating an action plan based on child screenings

processing results from child screenings

reflect on strategies to support results from caregivers screenings

reflective supervision

Pharmacological support

Reduce negative emotions

Conserving mental resources

professional development

proper workloads of staff/supervisors

criteria for staff selection are appropriate for population served

home visitor flexibility/ adaptability

home visitor sense of humor

reliable home visitor

motivational interviewing

active listening

relationship building

responsivness and sensitivity

Home visitor demonstrates cultural humility

empathetic communication

home visitor discipline regarding boundaries and limits of their role

Program trains staff on the prevalence, causes, and consequences of trauma

Program strengthening of service coordination

Role play/ coaching

Teaching problem solving skills to parents

Home visitor content mastery

Home visitor providing informal social support for families

Paradoxical instructions

Home visitor modeling of desired behaviors

Teaching relaxation/ self-regulation skills to parents

Culture of quality for implementing program

Home visitor observation of parent-child interactions

Organization/ program collaboration and outreach across the community

Model is based on a parenting framework

Teaching coping skills to parents

Program is data driven

recruitment of/outreach to parents

appropriate frequency of visits by the home visitor

Home visitor adaptability with respect to setting and participation

Culturally informed knowledge of the home visitor

Home visitor understands, affirms, and respects cultural identity of clients

Home visitor facilitates client connection to cultural and/or spiritual resources

Home visitor shares resources in client's Native language

Culturally attuned and responsive approach with

all staff training, strategies, materials

Antecedents

Please drag the element to the behavior change technique if it does the same thing

Items

Providing clients with linkage to services

Information sharing (by home visitor to client)

Teaching goal setting skills to parents

child assessment and screening

maternal risk assessment and screening

processing results from caregiver screenings

creating an action plan based on child screenings

processing results from child screenings

reflect on strategies to support results from caregivers screenings

reflective supervision

professional development

proper workloads of staff/supervisors

criteria for staff selection are appropriate for population served

home visitor flexibility/ adaptability

Restructuring the physical environment

Restructuring the social environment

Avoidance/reducing exposure to cues for the behavior

Distraction

home visitor sense of humor

reliable home visitor

motivational interviewing

active listening

relationship building

responsivness and sensitivity

Home visitor demonstrates cultural humility

empathetic communication

home visitor discipline regarding boundaries and limits of their role

Program trains staff on the prevalence, causes, and consequences of trauma

Program strengthening of service coordination

Role play/ coaching

Teaching problem solving skills to parents

Home visitor content mastery

Home visitor providing informal social support for families

Home visitor modeling of desired behaviors

Teaching relaxation/ self-regulation skills to parents

Culture of quality for implementing program

Home visitor observation of parent-child

Adding objects to the environment

Body changes

interactions

Organization/ program collaboration and outreach across the community

Model is based on a parenting framework

Teaching coping skills to parents

Program is data driven

recruitment of/outreach to parents

appropriate frequency of visits by the home visitor

Home visitor adaptability with respect to setting and participation

Culturally informed knowledge of the home visitor

Home visitor understands, affirms, and respects cultural identity of clients

Home visitor facilitates client connection to cultural and/or spiritual resources

Home visitor shares resources in client's Native language

Culturally attuned and responsive approach with all staff training, strategies, materials

---

Identity

Please drag the element to the behavior change technique if it does the same thing

Items	
Providing clients with linkage to services	**Identification of self as role model**
Information sharing (by home visitor to client)	
Teaching goal setting skills to parents	
child assessment and screening	**Framing/reframing**
maternal risk assessment and screening	
processing results from caregiver screenings	
creating an action plan based on child screenings	
processing results from child screenings	**Incompatible beliefs**
reflect on strategies to support results from caregivers screenings	
reflective supervision	
professional development	
proper workloads of staff/supervisors	
criteria for staff selection are appropriate for population served	**Valued self-identity**
home visitor flexibility/ adaptability	
home visitor sense of humor	
reliable home visitor	
motivational interviewing	**Identity associated with changed behavior**
active listening	
relationship building	

responsivness and sensitivity

Home visitor demonstrates cultural humility

empathetic communication

home visitor discipline regarding boundaries and limits of their role

Program trains staff on the prevalence, causes, and consequences of trauma

Program strengthening of service coordination

Role play/ coaching

Teaching problem solving skills to parents

Home visitor content mastery

Home visitor providing informal social support for families

Home visitor modeling of desired behaviors

Teaching relaxation/ self-regulation skills to parents

Culture of quality for implementing program

Home visitor observation of parent-child interactions

Organization/ program collaboration and outreach across the community

Model is based on a parenting framework

Teaching coping skills to parents

Program is data driven

recruitment of/outreach to parents

appropriate frequency of visits by the home visitor

Home visitor adaptability with respect to setting and participation

Culturally informed knowledge of the home visitor

Home visitor understands, affirms, and respects cultural identity of clients

Home visitor facilitates client connection to cultural and/or spiritual resources

Home visitor shares resources in client's Native language

Culturally attuned and responsive approach with all staff training, strategies, materials

Scheduled consequences

Please drag the element to the behavior change technique if it does the same thing

Items	Behavior cost
Providing clients with linkage to services	
Information sharing (by home visitor to client)	

Teaching goal setting skills to parents

child assessment and screening

maternal risk assessment and screening

processing results from caregiver screenings

creating an action plan based on child screenings

processing results from child screenings

reflect on strategies to support results from caregivers screenings

reflective supervision

professional development

proper workloads of staff/supervisors

criteria for staff selection are appropriate for population served

home visitor flexibility/ adaptability

home visitor sense of humor

reliable home visitor

motivational interviewing

active listening

relationship building

responsivness and sensitivity

Home visitor demonstrates cultural humility

empathetic communication

Punishment

Remove reward

Reward approximation

Rewarding completion

Situation-specific reward

home visitor discipline regarding boundaries and limits of their role

Program trains staff on the prevalence, causes, and consequences of trauma

Program strengthening of service coordination

Role play/ coaching

Teaching problem solving skills to parents

Home visitor content mastery

Home visitor providing informal social support for families

Home visitor modeling of desired behaviors

Teaching relaxation/ self-regulation skills to parents

Culture of quality for implementing program

Home visitor observation of parent-child interactions

Organization/ program collaboration and outreach across the community

Model is based on a parenting framework

Teaching coping skills to parents

Program is data driven

recruitment of/outreach to parents

appropriate frequency of visits by the home visitor

Reward incompatible behavior

Reward alternative behavior

Reduce reward frequency

Remove punishment

Home visitor adaptability with respect to setting and participation

Culturally informed knowledge of the home visitor

Home visitor understands, affirms, and respects cultural identity of clients

Home visitor facilitates client connection to cultural and/or spiritual resources

Home visitor shares resources in client's Native language

Culturally attuned and responsive approach with all staff training, strategies, materials

---

Self-belief

Please drag the element to the behavior change technique if it does the same thing

Items

Providing clients with linkage to services

Information sharing (by home visitor to client)

Teaching goal setting skills to parents

child assessment and screening

maternal risk assessment and screening

processing results from caregiver screenings

Verbal persuasion about capability

Mental rehearsal of successful performance

creating an action plan based on child screenings

processing results from child screenings

reflect on strategies to support results from caregivers screenings

reflective supervision

professional development

proper workloads of staff/supervisors

criteria for staff selection are appropriate for population served

home visitor flexibility/ adaptability

home visitor sense of humor

reliable home visitor

motivational interviewing

active listening

relationship building

responsivness and sensitivity

Home visitor demonstrates cultural humility

empathetic communication

home visitor discipline regarding boundaries and limits of their role

Program trains staff on the prevalence, causes, and consequences of trauma

Program strengthening of service coordination

Focus on past success

Self-talk

Role play/ coaching

Teaching problem solving skills to parents

Home visitor content mastery

Home visitor providing informal social support for families

Home visitor modeling of desired behaviors

Teaching relaxation/ self-regulation skills to parents

Culture of quality for implementing program

Home visitor observation of parent-child interactions

Organization/ program collaboration and outreach across the community

Model is based on a parenting framework

Teaching coping skills to parents

Program is data driven

recruitment of/outreach to parents

appropriate frequency of visits by the home visitor

Home visitor adaptability with respect to setting and participation

Culturally informed knowledge of the home visitor

Home visitor understands, affirms, and respects cultural identity of clients

Home visitor facilitates client connection to cultural and/or spiritual resources

Home visitor shares resources in client's Native language

Culturally attuned and responsive approach with all staff training, strategies, materials

---

Covert learning

Please drag the element to the behavior change technique if it does the same thing

Items

Providing clients with linkage to services

Information sharing (by home visitor to client)

Teaching goal setting skills to parents

child assessment and screening

maternal risk assessment and screening

processing results from caregiver screenings

creating an action plan based on child screenings

processing results from child screenings

reflect on strategies to support results from caregivers screenings

reflective supervision

Imaginary punishment

Imaginary reward

Vicarious consequences

professional development

proper workloads of staff/supervisors

criteria for staff selection are appropriate for population served

home visitor flexibility/ adaptability

home visitor sense of humor

reliable home visitor

motivational interviewing

active listening

relationship building

responsivness and sensitivity

Home visitor demonstrates cultural humility

empathetic communication

home visitor discipline regarding boundaries and limits of their role

Program trains staff on the prevalence, causes, and consequences of trauma

Program strengthening of service coordination

Role play/ coaching

Teaching problem solving skills to parents

Home visitor content mastery

Home visitor providing informal social support for families

Home visitor modeling of desired behaviors

Teaching relaxation/ self-regulation skills to parents

Culture of quality for implementing program

Home visitor observation of parent-child interactions

Organization/ program collaboration and outreach across the community

Model is based on a parenting framework

Teaching coping skills to parents

Program is data driven

recruitment of/outreach to parents

appropriate frequency of visits by the home visitor

Home visitor adaptability with respect to setting and participation

Culturally informed knowledge of the home visitor

Home visitor understands, affirms, and respects cultural identity of clients

Home visitor facilitates client connection to cultural and/or spiritual resources

Home visitor shares resources in client's Native language

Culturally attuned and responsive approach with

all staff training,
strategies, materials

Powered by Qualtrics
