# Supplementary material for: Expert-generated standard practice elements for evidence-based home visiting programs using a Delphi process
Source: PLoS One. 2022 Oct 17;17(10):e0275981. doi: 10.1371/journal.pone.0275981 (PMC9576067; doi:10.1371/journal.pone.0275981)
Supplement: S5 File — (PDF) [file pone.0275981.s005.pdf]

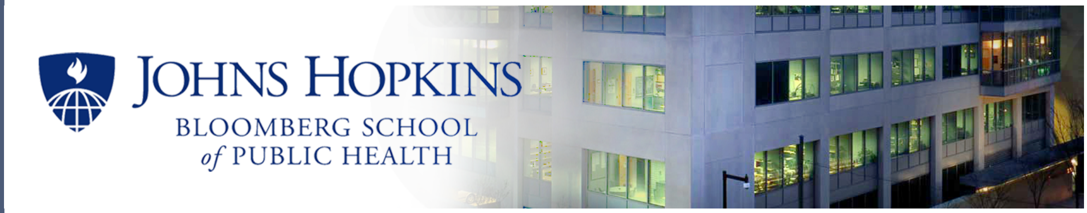

Introduction

In this survey, we will ask you to prioritize our panel-generated list of standard practice elements and behavior change techniques in terms of their utility to achieving specific home visiting outcome domains. The list of BCTs are taken from an existing taxonomy (developed as part of the Behaviour Change Project).

The outcome domains we have selected for this project are adapted from the Home Visiting Evidence of Effectiveness (HomVEE) review and work done by the Pew Home Visiting Data for Performance Initiative.

Because the list of elements/techniques is so long, you will be randomly assigned to rate elements/techniques for four outcome domains, one of them being related to tribal home visiting outcomes of interest.

Ratings (not necessary, useful, and essential) were chosen based on methods used in a previous study by McLeod et al.

To aid you in the completion of this survey, we have attached a list of the elements/techniques and their definitions: Elements techniques definitions 11 23 2020. You can also access this file in the email invitation we sent to you.

This survey is estimated to take 20-30 minutes to complete. Please feel free to save your work and continue as you have time.

Promotion of healthy physical child development (e.g. healthy eating, BF)

This section asks about **promotion of healthy physical child development (e.g. healthy eating, breastfeeding)**.

Please rate the following standard practice elements in terms of:

- **Not necessary** to use in early childhood evidence-based home visiting to achieve promotion of healthy physical child development
- **Useful but not essential** to use in early childhood evidence-based home visiting to achieve promotion of healthy physical child development OR
- **Essential** to use in early childhood evidence-based home visiting to achieve promotion of healthy physical child development

Promotion of healthy physical child development:

Please rate the following standard practice elements in terms of:

- **Not necessary** to use in early childhood evidence-based home visiting to achieve promotion of healthy physical child development
- **Useful but not essential** to use in early childhood evidence-based home visiting to achieve promotion of healthy physical child development OR
- **Essential** to use in early childhood evidence-based home visiting to achieve promotion of healthy physical child development

	Not necessary	Useful but not essential	Essential
Creating an action plan based on child screenings	○	○	○
Reflect on strategies to support results from caregivers screenings	○	○	○
Home visitor facilitates client connection to cultural and/or spiritual resources	○	○	○
Motivational interviewing	○	○	○
Teaching coping skills to parents	○	○	○
Teaching relaxation/ self-regulation skills to parents	○	○	○

Promotion of healthy physical child development:

Please rate the following standard practice elements in terms of:

- **Not necessary** to use in early childhood evidence-based home visiting to achieve promotion of healthy physical child development
- **Useful but not essential** to use in early childhood evidence-based home visiting to achieve promotion of healthy physical child development OR
- **Essential** to use in early childhood evidence-based home visiting to achieve promotion of healthy physical child development

	Not necessary	Useful but not essential	Essential
Home visitor shares resources in client's Native language	○	○	○
Providing clients with linkage to services	○	○	○
Child assessment and screening	○	○	○
Maternal risk assessment and screening	○	○	○
Reflective supervision	○	○	○
Professional development	○	○	○
Proper workloads of staff/ supervisors	○	○	○
Criteria for staff selection are appropriate for population served	○	○	○
Home visitor flexibility/ adaptability	○	○	○
Home visitor sense of humor	○	○	○
Reliable home visitor	○	○	○
Active listening	○	○	○

	Not necessary	Useful but not essential	Essential
Relationship building	◯	◯	◯
Responsiveness and sensitivity	◯	◯	◯
Home visitor demonstrates cultural humility	◯	◯	◯

Promotion of healthy physical child development

Please rate the following standard practice elements in terms of:

- **Not necessary** to use in early childhood evidence-based home visiting to achieve promotion of healthy physical child development
- **Useful but not essential** to use in early childhood evidence-based home visiting to achieve promotion of healthy physical child development OR
- **Essential** to use in early childhood evidence-based home visiting to achieve promotion of healthy physical child development

	Not necessary	Useful but not essential	Essential
Empathetic communication	◯	◯	◯
Home visitor discipline regarding boundaries and limits of their role	◯	◯	◯
Program trains staff on the prevalence, causes, and consequences of trauma	◯	◯	◯
Program strengthening of service coordination	◯	◯	◯
Home visitor content mastery	◯	◯	◯
Culture of quality for implementing program	◯	◯	◯

	Not necessary	Useful but not essential	Essential
Organization/ program collaboration and outreach across the community	○	○	○
Model is based on a parenting framework	○	○	○
Program is data driven	○	○	○
Recruitment of/outreach to parents	○	○	○
Appropriate frequency of visits by the home visitor	○	○	○
Home visitor adaptability with respect to setting and participation	○	○	○
Culturally informed knowledge of the home visitor	○	○	○
Home visitor understands, affirms, and respects cultural identity of clients	○	○	○
Culturally attuned and responsive approach with all staff training, strategies, materials	○	○	○

Promotion of healthy physical child development

The following items list a standard practice element *and* a behavior change technique that share similar definitions. Please rate the following pairs of elements in terms of:

- **Not necessary** to use in early childhood evidence-based home visiting to achieve promotion of healthy physical child development
- **Useful but not essential** to use in early childhood evidence-based home visiting to achieve promotion of healthy physical child development OR

- **Essential** to use in early childhood evidence-based home visiting to achieve promotion of healthy physical child development

	Not necessary	Useful but not essential	Essential
Teaching goal setting skills to parents / Goal setting (behavior)	○	○	○
Teaching problem solving skills to parents / Problem solving	○	○	○
Teaching goal setting skills to parents / Goal setting (outcome)	○	○	○
Home visitor observation of parent-child interactions / Monitoring of behavior by others without feedback	○	○	○
Processing results from child screenings; Processing results from caregiver screenings / Feedback on behavior	○	○	○
Home visitor providing informal social support for families / Social support (unspecified)	○	○	○
Information sharing (by home visitor to client) / Information about health consequences	○	○	○
Home visitor modeling of desired behaviors / Demonstration of the behavior	○	○	○

	Not necessary	Useful but not essential	Essential
Role play/coaching / Behavioral practice/rehearsal	○	○	○
Information sharing (by home visitor to client) / Credible source	○	○	○

Promotion of healthy physical child development:

Please rate the following behavior change techniques in terms of:

- **Not necessary** to use in early childhood evidence-based home visiting to achieve promotion of healthy physical child development
- **Useful but not essential** to use in early childhood evidence-based home visiting to achieve promotion of healthy physical child development OR
- **Essential** to use in early childhood evidence-based home visiting to achieve promotion of healthy physical child development

	Not necessary	Useful but not essential	Essential
1.4 Action planning	○	○	○
1.5 Review behavior goal(s)	○	○	○
1.6 Discrepancy between current behavior and goal	○	○	○
1.7 Review outcome goal(s)	○	○	○
1.8 Behavioral contract	○	○	○
1.9 Commitment	○	○	○
1.3 Goal setting (outcome)	○	○	○
2.3 Self-monitoring of behavior	○	○	○
2.4 Self-monitoring of outcome(s) of behavior	○	○	○

	Not necessary	Useful but not essential	Essential
2.5 Monitoring outcome(s) of behavior by others without feedback	○	○	○
2.6 Biofeedback	○	○	○
2.7 Feedback on outcome(s) of behavior	○	○	○
3.2 Social support (practical)	○	○	○
3.3 Social support (emotional)	○	○	○
4.1 Instruction on how to perform a behavior	○	○	○

Promotion of healthy physical child development:

Please rate the following behavior change techniques in terms of:

- **Not necessary** to use in early childhood evidence-based home visiting to achieve promotion of healthy physical child development
- **Useful but not essential** to use in early childhood evidence-based home visiting to achieve promotion of healthy physical child development OR
- **Essential** to use in early childhood evidence-based home visiting to achieve promotion of healthy physical child development

	Not necessary	Useful but not essential	Essential
4.2 Information about antecedents	○	○	○
4.3 Re-attribution	○	○	○
4.4 Behavioral experiments	○	○	○
5.2 Salience of consequences	○	○	○
5.3 Information about social and environmental consequences	○	○	○

	Not necessary	Useful but not essential	Essential
5.4 Monitoring of emotional consequences	○	○	○
5.5 Anticipated regret	○	○	○
5.6 Information about emotional consequences	○	○	○
6.2 Social comparison	○	○	○
6.3 Information about others' approval	○	○	○
7.1 Prompts/cues	○	○	○
7.2 Cue signalling reward	○	○	○
7.3 Reduce prompts/cues	○	○	○
7.4 Remove access to the reward	○	○	○
7.5 Remove aversive stimulus	○	○	○

Promotion of healthy physical child development:

Please rate the following behavior change techniques in terms of:

- **Not necessary** to use in early childhood evidence-based home visiting to achieve promotion of healthy physical child development
- **Useful but not essential** to use in early childhood evidence-based home visiting to achieve promotion of healthy physical child development OR
- **Essential** to use in early childhood evidence-based home visiting to achieve promotion of healthy physical child development

	Not necessary	Useful but not essential	Essential
7.6 Satiation	○	○	○
7.7 Exposure	○	○	○
7.8 Associative learning	○	○	○

	Not necessary	Useful but not essential	Essential
8.2 Behavior substitution	○	○	○
8.3 Habit formation	○	○	○
8.4 Habit reversal	○	○	○
8.5 Overcorrection	○	○	○
8.6 Generalisation of a target behavior	○	○	○
8.7 Graded tasks	○	○	○
9.2 Pros and cons	○	○	○
9.3 Comparative imagining of future outcomes	○	○	○
10.1 Material incentive (behavior)	○	○	○
10.2 Material reward(behavior)	○	○	○
10.3 Non-specific reward	○	○	○
10.4 Social reward	○	○	○

Promotion of healthy physical child development:

Please rate the following behavior change techniques in terms of:

- **Not necessary** to use in early childhood evidence-based home visiting to achieve promotion of healthy physical child development
- **Useful but not essential** to use in early childhood evidence-based home visiting to achieve promotion of healthy physical child development OR
- **Essential** to use in early childhood evidence-based home visiting to achieve promotion of healthy physical child development

	Not necessary	Useful but not essential	Essential
10.5 Social incentive	○	○	○
10.6 Non-specific incentive	○	○	○
10.7 Self-incentive	○	○	○

	Not necessary	Useful but not essential	Essential
10.8 Incentive (outcome)	○	○	○
10.9 Self-reward	○	○	○
10.10 Reward (outcome)	○	○	○
10.11 Future punishment	○	○	○
11.1 Pharmacological support	○	○	○
11.2 Reduce negative emotions	○	○	○
11.3 Conserving mental resources	○	○	○
11.4 Paradoxical instructions	○	○	○
12.1 Restructuring the physical environment	○	○	○
12.2 Restructuring the social environment	○	○	○
12.3 Avoidance/reducing exposure to cues for the behavior	○	○	○
12.4 Distraction	○	○	○

Promotion of healthy physical child development:

Please rate the following behavior change techniques in terms of:

- **Not necessary** to use in early childhood evidence-based home visiting to achieve promotion of healthy physical child development
- **Useful but not essential** to use in early childhood evidence-based home visiting to achieve promotion of healthy physical child development OR
- **Essential** to use in early childhood evidence-based home visiting to achieve promotion of healthy physical child development

	Not necessary	Useful but not essential	Essential

	Not necessary	Useful but not essential	Essential
12.5 Adding objects to the environment	○	○	○
12.6 Body changes	○	○	○
13.1 Identification of self as role model	○	○	○
13.2 Framing/reframing	○	○	○
13.3 Incompatible beliefs	○	○	○
13.4 Valued self-identity	○	○	○
13.5 Identity associated with changed behavior	○	○	○
14.1 Behavior cost	○	○	○
14.2 Punishment	○	○	○
14.3 Remove reward	○	○	○
14.4 Reward approximation	○	○	○
14.5 Rewarding completion	○	○	○
14.6 Situation-specific reward	○	○	○
14.7 Reward incompatible behavior	○	○	○
14.8 Reward alternative behavior	○	○	○

Promotion of healthy physical child development:

Please rate the following behavior change techniques in terms of:

- **Not necessary** to use in early childhood evidence-based home visiting to achieve promotion of healthy physical child development
- **Useful but not essential** to use in early childhood evidence-based home visiting to achieve promotion of healthy physical child development OR

- **Essential** to use in early childhood evidence-based home visiting to achieve promotion of healthy physical child development

	Not necessary	Useful but not essential	Essential
14.9 Reduce reward frequency	○	○	○
14.10 Remove punishment	○	○	○
15.1 Verbal persuasion about capability	○	○	○
15.2 Mental rehearsal of successful performance	○	○	○
15.3 Focus on past success	○	○	○
15.4 Self-talk	○	○	○
16.1 Imaginary punishment	○	○	○
16.2 Imaginary reward	○	○	○
16.3 Vicarious consequences	○	○	○

Promotion of social-emotional learning

This section asks about promotion of child development and school readiness, specifically the **promotion of social-emotional learning.**

Please rate the following standard practice elements in terms of:

- **Not necessary** to use in early childhood evidence-based home visiting to achieve promotion of social-emotional learning
- **Useful but not essential** to use in early childhood evidence-based home visiting to achieve promotion of social-emotional learning OR
- **Essential** to use in early childhood evidence-based home visiting to achieve promotion of social-emotional learning

Promotion of social-emotional learning:

Please rate the following standard practice elements in terms of:

- **Not necessary** to use in early childhood evidence-based home visiting to achieve promotion of social-emotional learning
- **Useful but not essential** to use in early childhood evidence-based home visiting to achieve promotion of social-emotional learning OR
- **Essential** to use in early childhood evidence-based home visiting to achieve promotion of social-emotional learning

	Not necessary	Useful but not essential	Essential
Creating an action plan based on child screenings	◯	◯	◯
Reflect on strategies to support results from caregivers screenings	◯	◯	◯
Home visitor facilitates client connection to cultural and/or spiritual resources	◯	◯	◯
Motivational interviewing	◯	◯	◯
Teaching coping skills to parents	◯	◯	◯
Teaching relaxation/ self-regulation skills to parents	◯	◯	◯

Promotion of social-emotional learning:

Please rate the following standard practice elements in terms of:

- **Not necessary** to use in early childhood evidence-based home visiting to achieve promotion of social-emotional learning

- **Useful but not essential** to use in early childhood evidence-based home visiting to achieve promotion of social-emotional learning OR
- **Essential** to use in early childhood evidence-based home visiting to achieve promotion of social-emotional learning

	Not necessary	Useful but not essential	Essential
Home visitor shares resources in client's Native language	○	○	○
Providing clients with linkage to services	○	○	○
Child assessment and screening	○	○	○
Maternal risk assessment and screening	○	○	○
Reflective supervision	○	○	○
Professional development	○	○	○
Proper workloads of staff/ supervisors	○	○	○
Criteria for staff selection are appropriate for population served	○	○	○
Home visitor flexibility/ adaptability	○	○	○
Home visitor sense of humor	○	○	○
Reliable home visitor	○	○	○
Active listening	○	○	○
Relationship building	○	○	○
Responsiveness and sensitivity	○	○	○
Home visitor demonstrates cultural humility	○	○	○

Promotion of social-emotional learning:

Please rate the following standard practice elements in terms of:

- **Not necessary** to use in early childhood evidence-based home visiting to achieve promotion of social-emotional learning
- **Useful but not essential** to use in early childhood evidence-based home visiting to achieve promotion of social-emotional learning OR
- **Essential** to use in early childhood evidence-based home visiting to achieve promotion of social-emotional learning

	Not necessary	Useful but not essential	Essential
Empathetic communication	○	○	○
Home visitor discipline regarding boundaries and limits of their role	○	○	○
Program trains staff on the prevalence, causes, and consequences of trauma	○	○	○
Program strengthening of service coordination	○	○	○
Home visitor content mastery	○	○	○
Culture of quality for implementing program	○	○	○
Organization/ program collaboration and outreach across the community	○	○	○
Model is based on a parenting framework	○	○	○
Program is data driven	○	○	○

	Not necessary	Useful but not essential	Essential
Recruitment of/outreach to parents	O	O	O
Appropriate frequency of visits by the home visitor	O	O	O
Home visitor adaptability with respect to setting and participation	O	O	O
Culturally informed knowledge of the home visitor	O	O	O
Home visitor understands, affirms, and respects cultural identity of clients	O	O	O
Culturally attuned and responsive approach with all staff training, strategies, materials	O	O	O

Promotion of social-emotional learning:

The following items list a standard practice element *and* a behavior change technique that share similar definitions. Please rate the following pairs of elements in terms of:

- **Not necessary** to use in early childhood evidence-based home visiting to achieve promotion of social-emotional learning
- **Useful but not essential** to use in early childhood evidence-based home visiting to achieve promotion of social-emotional learning OR
- **Essential** to use in early childhood evidence-based home visiting to achieve promotion of social-emotional learning

	Not necessary	Useful but not essential	Essential
Teaching goal setting skills to parents / Goal setting (behavior)	O	O	O

	Not necessary	Useful but not essential	Essential
Teaching problem solving skills to parents / Problem solving	○	○	○
Teaching goal setting skills to parents / Goal setting (outcome)	○	○	○
Home visitor observation of parent-child interactions / Monitoring of behavior by others without feedback	○	○	○
Processing results from child screenings; Processing results from caregiver screenings / Feedback on behavior	○	○	○
Home visitor providing informal social support for families / Social support (unspecified)	○	○	○
Information sharing (by home visitor to client) / Information about health consequences	○	○	○
Home visitor modeling of desired behaviors / Demonstration of the behavior	○	○	○
Role play/coaching / Behavioral practice/rehearsal	○	○	○
Information sharing (by home visitor to client) / Credible source	○	○	○

Promotion of social-emotional learning:

Please rate the following behavior change techniques in terms of:

- **Not necessary** to use in early childhood evidence-based home visiting to achieve promotion of social-emotional learning
- **Useful but not essential** to use in early childhood evidence-based home visiting to achieve promotion of social-emotional learning OR
- **Essential** to use in early childhood evidence-based home visiting to achieve promotion of social-emotional learning

	Not necessary	Useful but not essential	Essential
1.4 Action planning	○	○	○
1.5 Review behavior goal(s)	○	○	○
1.6 Discrepancy between current behavior and goal	○	○	○
1.7 Review outcome goal(s)	○	○	○
1.8 Behavioral contract	○	○	○
1.9 Commitment	○	○	○
1.3 Goal setting (outcome)	○	○	○
2.3 Self-monitoring of behavior	○	○	○
2.4 Self-monitoring of outcome(s) of behavior	○	○	○
2.5 Monitoring outcome(s) of behavior by others without feedback	○	○	○
2.6 Biofeedback	○	○	○
2.7 Feedback on outcome(s) of behavior	○	○	○
3.2 Social support (practical)	○	○	○
3.3 Social support (emotional)	○	○	○

	Not necessary	Useful but not essential	Essential
4.1 Instruction on how to perform a behavior	○	○	○

Promotion of social-emotional learning:

Please rate the following behavior change techniques in terms of:

- **Not necessary** to use in early childhood evidence-based home visiting to achieve promotion of social-emotional learning
- **Useful but not essential** to use in early childhood evidence-based home visiting to achieve promotion of social-emotional learning OR
- **Essential** to use in early childhood evidence-based home visiting to achieve promotion of social-emotional learning

	Not necessary	Useful but not essential	Essential
4.2 Information about antecedents	○	○	○
4.3 Re-attribution	○	○	○
4.4 Behavioral experiments	○	○	○
5.2 Salience of consequences	○	○	○
5.3 Information about social and environmental consequences	○	○	○
5.4 Monitoring of emotional consequences	○	○	○
5.5 Anticipated regret	○	○	○
5.6 Information about emotional consequences	○	○	○
6.2 Social comparison	○	○	○
6.3 Information about others' approval	○	○	○

	Not necessary	Useful but not essential	Essential
7.1 Prompts/cues	○	○	○
7.2 Cue signalling reward	○	○	○
7.3 Reduce prompts/cues	○	○	○
7.4 Remove access to the reward	○	○	○
7.5 Remove aversive stimulus	○	○	○

Promotion of social-emotional learning:

Please rate the following behavior change techniques in terms of:

- **Not necessary** to use in early childhood evidence-based home visiting to achieve promotion of social-emotional learning
- **Useful but not essential** to use in early childhood evidence-based home visiting to achieve promotion of social-emotional learning OR
- **Essential** to use in early childhood evidence-based home visiting to achieve promotion of social-emotional learning

	Not necessary	Useful but not essential	Essential
7.6 Satiation	○	○	○
7.7 Exposure	○	○	○
7.8 Associative learning	○	○	○
8.2 Behavior substitution	○	○	○
8.3 Habit formation	○	○	○
8.4 Habit reversal	○	○	○
8.5 Overcorrection	○	○	○
8.6 Generalisation of a target behavior	○	○	○
8.7 Graded tasks	○	○	○
9.2 Pros and cons	○	○	○

	Not necessary	Useful but not essential	Essential
9.3 Comparative imagining of future outcomes	○	○	○
10.1 Material incentive (behavior)	○	○	○
10.2 Material reward(behavior)	○	○	○
10.3 Non-specific reward	○	○	○
10.4 Social reward	○	○	○

Promotion of social-emotional learning:

Please rate the following behavior change techniques in terms of:

- **Not necessary** to use in early childhood evidence-based home visiting to achieve promotion of social-emotional learning
- **Useful but not essential** to use in early childhood evidence-based home visiting to achieve promotion of social-emotional learning OR
- **Essential** to use in early childhood evidence-based home visiting to achieve promotion of social-emotional learning

	Not necessary	Useful but not essential	Essential
10.5 Social incentive	○	○	○
10.6 Non-specific incentive	○	○	○
10.7 Self-incentive	○	○	○
10.8 Incentive (outcome)	○	○	○
10.9 Self-reward	○	○	○
10.10 Reward (outcome)	○	○	○
10.11 Future punishment	○	○	○
11.1 Pharmacological support	○	○	○

	Not necessary	Useful but not essential	Essential
11.2 Reduce negative emotions	○	○	○
11.3 Conserving mental resources	○	○	○
11.4 Paradoxical instructions	○	○	○
12.1 Restructuring the physical environment	○	○	○
12.2 Restructuring the social environment	○	○	○
12.3 Avoidance/reducing exposure to cues for the behavior	○	○	○
12.4 Distraction	○	○	○

Promotion of social-emotional learning:

Please rate the following behavior change techniques in terms of:

- **Not necessary** to use in early childhood evidence-based home visiting to achieve promotion of social-emotional learning
- **Useful but not essential** to use in early childhood evidence-based home visiting to achieve promotion of social-emotional learning OR
- **Essential** to use in early childhood evidence-based home visiting to achieve promotion of social-emotional learning

	Not necessary	Useful but not essential	Essential
12.5 Adding objects to the environment	○	○	○
12.6 Body changes	○	○	○
13.1 Identification of self as role model	○	○	○
13.2 Framing/reframing	○	○	○

	Not necessary	Useful but not essential	Essential
13.3 Incompatible beliefs	○	○	○
13.4 Valued self-identity	○	○	○
13.5 Identity associated with changed behavior	○	○	○
14.1 Behavior cost	○	○	○
14.2 Punishment	○	○	○
14.3 Remove reward	○	○	○
14.4 Reward approximation	○	○	○
14.5 Rewarding completion	○	○	○
14.6 Situation-specific reward	○	○	○
14.7 Reward incompatible behavior	○	○	○
14.8 Reward alternative behavior	○	○	○

Promotion of social-emotional learning:

Please rate the following behavior change techniques in terms of:

- **Not necessary** to use in early childhood evidence-based home visiting to achieve promotion of social-emotional learning
- **Useful but not essential** to use in early childhood evidence-based home visiting to achieve promotion of social-emotional learning OR
- **Essential** to use in early childhood evidence-based home visiting to achieve promotion of social-emotional learning

	Not necessary	Useful but not essential	Essential
14.9 Reduce reward frequency	○	○	○
14.10 Remove punishment	○	○	○

	Not necessary	Useful but not essential	Essential
15.1 Verbal persuasion about capability	○	○	○
15.2 Mental rehearsal of successful performance	○	○	○
15.3 Focus on past success	○	○	○
15.4 Self-talk	○	○	○
16.1 Imaginary punishment	○	○	○
16.2 Imaginary reward	○	○	○
16.3 Vicarious consequences	○	○	○

Improving cognitive development (e.g. language development)

This section asks about promotion of child development and school readiness, specifically **Improving cognitive development (e.g. language development).**

Please rate the following standard practice elements in terms of:

- **Not necessary** to use in early childhood evidence-based home visiting to achieve improving cognitive development
- **Useful but not essential** to use in early childhood evidence-based home visiting to achieve improving cognitive development OR
- **Essential** to use in early childhood evidence-based home visiting to achieve improving cognitive development

Improving cognitive development (e.g. language development):

Please rate the following standard practice elements in terms of:

- **Not necessary** to use in early childhood evidence-based home visiting to achieve improving cognitive development

- **Useful but not essential** to use in early childhood evidence-based home visiting to achieve improving cognitive development OR
- **Essential** to use in early childhood evidence-based home visiting to achieve improving cognitive development

	Not necessary	Useful but not essential	Essential
Creating an action plan based on child screenings	○	○	○
Reflect on strategies to support results from caregivers screenings	○	○	○
Home visitor facilitates client connection to cultural and/or spiritual resources	○	○	○
Motivational interviewing	○	○	○
Teaching coping skills to parents	○	○	○
Teaching relaxation/ self-regulation skills to parents	○	○	○

Improving cognitive development (e.g. language development):

Please rate the following standard practice elements in terms of:

- **Not necessary** to use in early childhood evidence-based home visiting to achieve improving cognitive development
- **Useful but not essential** to use in early childhood evidence-based home visiting to achieve improving cognitive development OR
- **Essential** to use in early childhood evidence-based home visiting to achieve improving cognitive development

	Not necessary	Useful but not essential	Essential

	Not necessary	Useful but not essential	Essential
Home visitor shares resources in client's Native language	○	○	○
Providing clients with linkage to services	○	○	○
Child assessment and screening	○	○	○
Maternal risk assessment and screening	○	○	○
Reflective supervision	○	○	○
Professional development	○	○	○
Proper workloads of staff/ supervisors	○	○	○
Criteria for staff selection are appropriate for population served	○	○	○
Home visitor flexibility/ adaptability	○	○	○
Home visitor sense of humor	○	○	○
Reliable home visitor	○	○	○
Active listening	○	○	○
Relationship building	○	○	○
Responsiveness and sensitivity	○	○	○
Home visitor demonstrates cultural humility	○	○	○

Improving cognitive development (e.g. language development):

Please rate the following standard practice elements in terms of:

- **Not necessary** to use in early childhood evidence-based home visiting to achieve improving cognitive development
- **Useful but not essential** to use in early childhood evidence-based home visiting to achieve improving cognitive development OR
- **Essential** to use in early childhood evidence-based home visiting to achieve improving cognitive development

	Not necessary	Useful but not essential	Essential
Empathetic communication	○	○	○
Home visitor discipline regarding boundaries and limits of their role	○	○	○
Program trains staff on the prevalence, causes, and consequences of trauma	○	○	○
Program strengthening of service coordination	○	○	○
Home visitor content mastery	○	○	○
Culture of quality for implementing program	○	○	○
Organization/ program collaboration and outreach across the community	○	○	○
Model is based on a parenting framework	○	○	○
Program is data driven	○	○	○
Recruitment of/outreach to parents	○	○	○
Appropriate frequency of visits by the home visitor	○	○	○

	Not necessary	Useful but not essential	Essential
Home visitor adaptability with respect to setting and participation	O	O	O
Culturally informed knowledge of the home visitor	O	O	O
Home visitor understands, affirms, and respects cultural identity of clients	O	O	O
Culturally attuned and responsive approach with all staff training, strategies, materials	O	O	O

Improving cognitive development (e.g. language development):

The following items list a standard practice element *and* a behavior change technique that share similar definitions. Please rate the following pairs of elements in terms of:

- **Not necessary** to use in early childhood evidence-based home visiting to achieve improving cognitive development
- **Useful but not essential** to use in early childhood evidence-based home visiting to achieve improving cognitive development OR
- **Essential** to use in early childhood evidence-based home visiting to achieve improving cognitive development

	Not necessary	Useful but not essential	Essential
Teaching goal setting skills to parents / Goal setting (behavior)	O	O	O
Teaching problem solving skills to parents / Problem solving	O	O	O

	Not necessary	Useful but not essential	Essential
Teaching goal setting skills to parents / Goal setting (outcome)	O	O	O
Home visitor observation of parent-child interactions / Monitoring of behavior by others without feedback	O	O	O
Processing results from child screenings; Processing results from caregiver screenings / Feedback on behavior	O	O	O
Home visitor providing informal social support for families / Social support (unspecified)	O	O	O
Information sharing (by home visitor to client) / Information about health consequences	O	O	O
Home visitor modeling of desired behaviors / Demonstration of the behavior	O	O	O
Role play/coaching / Behavioral practice/rehearsal	O	O	O
Information sharing (by home visitor to client) / Credible source	O	O	O

Improving cognitive development (e.g. language development):

Please rate the following behavior change techniques in terms of:

- **Not necessary** to use in early childhood evidence-based home visiting to achieve improving cognitive development
- **Useful but not essential** to use in early childhood evidence-based home visiting to achieve improving cognitive development OR
- **Essential** to use in early childhood evidence-based home visiting to achieve improving cognitive development

	Not necessary	Useful but not essential	Essential
1.4 Action planning	◯	◯	◯
1.5 Review behavior goal(s)	◯	◯	◯
1.6 Discrepancy between current behavior and goal	◯	◯	◯
1.7 Review outcome goal(s)	◯	◯	◯
1.8 Behavioral contract	◯	◯	◯
1.9 Commitment	◯	◯	◯
1.3 Goal setting (outcome)	◯	◯	◯
2.3 Self-monitoring of behavior	◯	◯	◯
2.4 Self-monitoring of outcome(s) of behavior	◯	◯	◯
2.5 Monitoring outcome(s) of behavior by others without feedback	◯	◯	◯
2.6 Biofeedback	◯	◯	◯
2.7 Feedback on outcome(s) of behavior	◯	◯	◯
3.2 Social support (practical)	◯	◯	◯
3.3 Social support (emotional)	◯	◯	◯
4.1 Instruction on how to perform a behavior	◯	◯	◯

Improving cognitive development (e.g. language development):

Please rate the following behavior change techniques in terms of:

- **Not necessary** to use in early childhood evidence-based home visiting to achieve improving cognitive development
- **Useful but not essential** to use in early childhood evidence-based home visiting to achieve improving cognitive development OR
- **Essential** to use in early childhood evidence-based home visiting to achieve improving cognitive development

	Not necessary	Useful but not essential	Essential
4.2 Information about antecedents	○	○	○
4.3 Re-attribution	○	○	○
4.4 Behavioral experiments	○	○	○
5.2 Salience of consequences	○	○	○
5.3 Information about social and environmental consequences	○	○	○
5.4 Monitoring of emotional consequences	○	○	○
5.5 Anticipated regret	○	○	○
5.6 Information about emotional consequences	○	○	○
6.2 Social comparison	○	○	○
6.3 Information about others' approval	○	○	○
7.1 Prompts/cues	○	○	○
7.2 Cue signalling reward	○	○	○
7.3 Reduce prompts/cues	○	○	○

	Not necessary	Useful but not essential	Essential
7.4 Remove access to the reward	○	○	○
7.5 Remove aversive stimulus	○	○	○

Improving cognitive development (e.g. language development):

Please rate the following behavior change techniques in terms of:

- **Not necessary** to use in early childhood evidence-based home visiting to achieve improving cognitive development
- **Useful but not essential** to use in early childhood evidence-based home visiting to achieve improving cognitive development OR
- **Essential** to use in early childhood evidence-based home visiting to achieve improving cognitive development

	Not necessary	Useful but not essential	Essential
7.6 Satiation	○	○	○
7.7 Exposure	○	○	○
7.8 Associative learning	○	○	○
8.2 Behavior substitution	○	○	○
8.3 Habit formation	○	○	○
8.4 Habit reversal	○	○	○
8.5 Overcorrection	○	○	○
8.6 Generalisation of a target behavior	○	○	○
8.7 Graded tasks	○	○	○
9.2 Pros and cons	○	○	○
9.3 Comparative imagining of future outcomes	○	○	○
10.1 Material incentive (behavior)	○	○	○
10.2 Material reward(behavior)	○	○	○

	Not necessary	Useful but not essential	Essential
10.3 Non-specific reward	○	○	○
10.4 Social reward	○	○	○

Improving cognitive development (e.g. language development):

Please rate the following behavior change techniques in terms of:

- **Not necessary** to use in early childhood evidence-based home visiting to achieve improving cognitive development
- **Useful but not essential** to use in early childhood evidence-based home visiting to achieve improving cognitive development OR
- **Essential** to use in early childhood evidence-based home visiting to achieve improving cognitive development

	Not necessary	Useful but not essential	Essential
10.5 Social incentive	○	○	○
10.6 Non-specific incentive	○	○	○
10.7 Self-incentive	○	○	○
10.8 Incentive (outcome)	○	○	○
10.9 Self-reward	○	○	○
10.10 Reward (outcome)	○	○	○
10.11 Future punishment	○	○	○
11.1 Pharmacological support	○	○	○
11.2 Reduce negative emotions	○	○	○
11.3 Conserving mental resources	○	○	○
11.4 Paradoxical instructions	○	○	○

	Not necessary	Useful but not essential	Essential
12.1 Restructuring the physical environment	○	○	○
12.2 Restructuring the social environment	○	○	○
12.3 Avoidance/reducing exposure to cues for the behavior	○	○	○
12.4 Distraction	○	○	○

Improving cognitive development (e.g. language development):

Please rate the following behavior change techniques in terms of:

- **Not necessary** to use in early childhood evidence-based home visiting to achieve improving cognitive development
- **Useful but not essential** to use in early childhood evidence-based home visiting to achieve improving cognitive development OR
- **Essential** to use in early childhood evidence-based home visiting to achieve improving cognitive development

	Not necessary	Useful but not essential	Essential
12.5 Adding objects to the environment	○	○	○
12.6 Body changes	○	○	○
13.1 Identification of self as role model	○	○	○
13.2 Framing/reframing	○	○	○
13.3 Incompatible beliefs	○	○	○
13.4 Valued self-identity	○	○	○
13.5 Identity associated with changed behavior	○	○	○

	Not necessary	Useful but not essential	Essential
14.1 Behavior cost	○	○	○
14.2 Punishment	○	○	○
14.3 Remove reward	○	○	○
14.4 Reward approximation	○	○	○
14.5 Rewarding completion	○	○	○
14.6 Situation-specific reward	○	○	○
14.7 Reward incompatible behavior	○	○	○
14.8 Reward alternative behavior	○	○	○

Improving cognitive development (e.g. language development):

Please rate the following behavior change techniques in terms of:

- **Not necessary** to use in early childhood evidence-based home visiting to achieve improving cognitive development
- **Useful but not essential** to use in early childhood evidence-based home visiting to achieve improving cognitive development OR
- **Essential** to use in early childhood evidence-based home visiting to achieve improving cognitive development

	Not necessary	Useful but not essential	Essential
14.9 Reduce reward frequency	○	○	○
14.10 Remove punishment	○	○	○
15.1 Verbal persuasion about capability	○	○	○
15.2 Mental rehearsal of successful performance	○	○	○

	Not necessary	Useful but not essential	Essential
15.3 Focus on past success	O	O	O
15.4 Self-talk	O	O	O
16.1 Imaginary punishment	O	O	O
16.2 Imaginary reward	O	O	O
16.3 Vicarious consequences	O	O	O

Linkages and coordination of referrals for other community resources and supports

This section asks about **linkages and coordination of referrals for other community resources and supports**.
Please rate the following standard practice elements in terms of:

- **Not necessary** to use in early childhood evidence-based home visiting to achieve linkages and coordination of referrals for other community resources and supports
- **Useful but not essential** to use in early childhood evidence-based home visiting to achieve linkages and coordination of referrals for other community resources and supports OR
- **Essential** to use in early childhood evidence-based home visiting to achieve linkages and coordination of referrals for other community resources and supports

Linkages and coordination of referrals for other community resources and supports:

Please rate the following standard practice elements in terms of:

- **Not necessary** to use in early childhood evidence-based home visiting to achieve linkages and coordination of referrals for other community resources and supports

- **Useful but not essential** to use in early childhood evidence-based home visiting to achieve linkages and coordination of referrals for other community resources and supports OR
- **Essential** to use in early childhood evidence-based home visiting to achieve linkages and coordination of referrals for other community resources and supports

	Not necessary	Useful but not essential	Essential
Creating an action plan based on child screenings	○	○	○
Reflect on strategies to support results from caregivers screenings	○	○	○
Home visitor facilitates client connection to cultural and/or spiritual resources	○	○	○
Motivational interviewing	○	○	○
Teaching coping skills to parents	○	○	○
Teaching relaxation/ self-regulation skills to parents	○	○	○

Linkages and coordination of referrals for other community resources and supports:

Please rate the following standard practice elements in terms of:

- **Not necessary** to use in early childhood evidence-based home visiting to achieve linkages and coordination of referrals for other community resources and supports
- **Useful but not essential** to use in early childhood evidence-based home visiting to achieve linkages and coordination of referrals for other community resources and supports OR

- **Essential** to use in early childhood evidence-based home visiting to achieve linkages and coordination of referrals for other community resources and supports

	Not necessary	Useful but not essential	Essential
Home visitor shares resources in client's Native language	○	○	○
Providing clients with linkage to services	○	○	○
Child assessment and screening	○	○	○
Maternal risk assessment and screening	○	○	○
Reflective supervision	○	○	○
Professional development	○	○	○
Proper workloads of staff/ supervisors	○	○	○
Criteria for staff selection are appropriate for population served	○	○	○
Home visitor flexibility/ adaptability	○	○	○
Home visitor sense of humor	○	○	○
Reliable home visitor	○	○	○
Active listening	○	○	○
Relationship building	○	○	○
Responsiveness and sensitivity	○	○	○
Home visitor demonstrates cultural humility	○	○	○

Linkages and coordination of referrals for other community resources and supports:

Please rate the following standard practice elements in terms of:

- **Not necessary** to use in early childhood evidence-based home visiting to achieve linkages and coordination of referrals for other community resources and supports
- **Useful but not essential** to use in early childhood evidence-based home visiting to achieve linkages and coordination of referrals for other community resources and supports OR
- **Essential** to use in early childhood evidence-based home visiting to achieve linkages and coordination of referrals for other community resources and supports

	Not necessary	Useful but not essential	Essential
Empathetic communication	○	○	○
Home visitor discipline regarding boundaries and limits of their role	○	○	○
Program trains staff on the prevalence, causes, and consequences of trauma	○	○	○
Program strengthening of service coordination	○	○	○
Home visitor content mastery	○	○	○
Culture of quality for implementing program	○	○	○
Organization/ program collaboration and outreach across the community	○	○	○

	Not necessary	Useful but not essential	Essential
Model is based on a parenting framework	○	○	○
Program is data driven	○	○	○
Recruitment of/outreach to parents	○	○	○
Appropriate frequency of visits by the home visitor	○	○	○
Home visitor adaptability with respect to setting and participation	○	○	○
Culturally informed knowledge of the home visitor	○	○	○
Home visitor understands, affirms, and respects cultural identity of clients	○	○	○
Culturally attuned and responsive approach with all staff training, strategies, materials	○	○	○

Linkages and coordination of referrals for other community resources and supports:

The following items list a standard practice element *and* a behavior change technique that share similar definitions. Please rate the following pairs of elements in terms of:

- **Not necessary** to use in early childhood evidence-based home visiting to achieve linkages and coordination of referrals for other community resources and supports
- **Useful but not essential** to use in early childhood evidence-based home visiting to achieve linkages and coordination of referrals for other community resources and supports OR

- **Essential** to use in early childhood evidence-based home visiting to achieve linkages and coordination of referrals for other community resources and supports

	Not necessary	Useful but not essential	Essential
Teaching goal setting skills to parents / Goal setting (behavior)	○	○	○
Teaching problem solving skills to parents / Problem solving	○	○	○
Teaching goal setting skills to parents / Goal setting (outcome)	○	○	○
Home visitor observation of parent-child interactions / Monitoring of behavior by others without feedback	○	○	○
Processing results from child screenings; Processing results from caregiver screenings / Feedback on behavior	○	○	○
Home visitor providing informal social support for families / Social support (unspecified)	○	○	○
Information sharing (by home visitor to client) / Information about health consequences	○	○	○
Home visitor modeling of desired behaviors / Demonstration of the behavior	○	○	○

	Not necessary	Useful but not essential	Essential
Role play/coaching / Behavioral practice/rehearsal	O	O	O
Information sharing (by home visitor to client) / Credible source	O	O	O

Linkages and coordination of referrals for other community resources and supports:

Please rate the following behavior change techniques in terms of:

- **Not necessary** to use in early childhood evidence-based home visiting to achieve linkages and coordination of referrals for other community resources and supports
- **Useful but not essential** to use in early childhood evidence-based home visiting to achieve linkages and coordination of referrals for other community resources and supports OR
- **Essential** to use in early childhood evidence-based home visiting to achieve linkages and coordination of referrals for other community resources and supports

	Not necessary	Useful but not essential	Essential
1.4 Action planning	O	O	O
1.5 Review behavior goal(s)	O	O	O
1.6 Discrepancy between current behavior and goal	O	O	O
1.7 Review outcome goal(s)	O	O	O
1.8 Behavioral contract	O	O	O
1.9 Commitment	O	O	O
1.3 Goal setting (outcome)	O	O	O
2.3 Self-monitoring of behavior	O	O	O

	Not necessary	Useful but not essential	Essential
2.4 Self-monitoring of outcome(s) of behavior	○	○	○
2.5 Monitoring outcome(s) of behavior by others without feedback	○	○	○
2.6 Biofeedback	○	○	○
2.7 Feedback on outcome(s) of behavior	○	○	○
3.2 Social support (practical)	○	○	○
3.3 Social support (emotional)	○	○	○
4.1 Instruction on how to perform a behavior	○	○	○

Linkages and coordination of referrals for other community resources and supports:

Please rate the following behavior change techniques in terms of:

- **Not necessary** to use in early childhood evidence-based home visiting to achieve linkages and coordination of referrals for other community resources and supports
- **Useful but not essential** to use in early childhood evidence-based home visiting to achieve linkages and coordination of referrals for other community resources and supports OR
- **Essential** to use in early childhood evidence-based home visiting to achieve linkages and coordination of referrals for other community resources and supports

	Not necessary	Useful but not essential	Essential
4.2 Information about antecedents	○	○	○
4.3 Re-attribution	○	○	○

	Not necessary	Useful but not essential	Essential
4.4 Behavioral experiments	○	○	○
5.2 Salience of consequences	○	○	○
5.3 Information about social and environmental consequences	○	○	○
5.4 Monitoring of emotional consequences	○	○	○
5.5 Anticipated regret	○	○	○
5.6 Information about emotional consequences	○	○	○
6.2 Social comparison	○	○	○
6.3 Information about others' approval	○	○	○
7.1 Prompts/cues	○	○	○
7.2 Cue signalling reward	○	○	○
7.3 Reduce prompts/cues	○	○	○
7.4 Remove access to the reward	○	○	○
7.5 Remove aversive stimulus	○	○	○

Linkages and coordination of referrals for other community resources and supports:

Please rate the following behavior change techniques in terms of:

- **Not necessary** to use in early childhood evidence-based home visiting to achieve linkages and coordination of referrals for other community resources and supports
- **Useful but not essential** to use in early childhood evidence-based home visiting to achieve linkages and coordination of referrals for other community

resources and supports OR

- **Essential** to use in early childhood evidence-based home visiting to achieve linkages and coordination of referrals for other community resources and supports

	Not necessary	Useful but not essential	Essential
7.6 Satiation	O	O	O
7.7 Exposure	O	O	O
7.8 Associative learning	O	O	O
8.2 Behavior substitution	O	O	O
8.3 Habit formation	O	O	O
8.4 Habit reversal	O	O	O
8.5 Overcorrection	O	O	O
8.6 Generalisation of a target behavior	O	O	O
8.7 Graded tasks	O	O	O
9.2 Pros and cons	O	O	O
9.3 Comparative imagining of future outcomes	O	O	O
10.1 Material incentive (behavior)	O	O	O
10.2 Material reward(behavior)	O	O	O
10.3 Non-specific reward	O	O	O
10.4 Social reward	O	O	O

Linkages and coordination of referrals for other community resources and supports:

Please rate the following behavior change techniques in terms of:

- **Not necessary** to use in early childhood evidence-based home visiting to achieve linkages and coordination of referrals for other community resources and supports

- **Useful but not essential** to use in early childhood evidence-based home visiting to achieve linkages and coordination of referrals for other community resources and supports OR
- **Essential** to use in early childhood evidence-based home visiting to achieve linkages and coordination of referrals for other community resources and supports

	Not necessary	Useful but not essential	Essential
10.5 Social incentive	○	○	○
10.6 Non-specific incentive	○	○	○
10.7 Self-incentive	○	○	○
10.8 Incentive (outcome)	○	○	○
10.9 Self-reward	○	○	○
10.10 Reward (outcome)	○	○	○
10.11 Future punishment	○	○	○
11.1 Pharmacological support	○	○	○
11.2 Reduce negative emotions	○	○	○
11.3 Conserving mental resources	○	○	○
11.4 Paradoxical instructions	○	○	○
12.1 Restructuring the physical environment	○	○	○
12.2 Restructuring the social environment	○	○	○
12.3 Avoidance/reducing exposure to cues for the behavior	○	○	○
12.4 Distraction	○	○	○

Linkages and coordination of referrals for other community resources and supports:

Please rate the following behavior change techniques in terms of:

- **Not necessary** to use in early childhood evidence-based home visiting to achieve linkages and coordination of referrals for other community resources and supports
- **Useful but not essential** to use in early childhood evidence-based home visiting to achieve linkages and coordination of referrals for other community resources and supports OR
- **Essential** to use in early childhood evidence-based home visiting to achieve linkages and coordination of referrals for other community resources and supports

	Not necessary	Useful but not essential	Essential
12.5 Adding objects to the environment	○	○	○
12.6 Body changes	○	○	○
13.1 Identification of self as role model	○	○	○
13.2 Framing/reframing	○	○	○
13.3 Incompatible beliefs	○	○	○
13.4 Valued self-identity	○	○	○
13.5 Identity associated with changed behavior	○	○	○
14.1 Behavior cost	○	○	○
14.2 Punishment	○	○	○
14.3 Remove reward	○	○	○
14.4 Reward approximation	○	○	○
14.5 Rewarding completion	○	○	○

	Not necessary	Useful but not essential	Essential
14.6 Situation-specific reward	○	○	○
14.7 Reward incompatible behavior	○	○	○
14.8 Reward alternative behavior	○	○	○

Linkages and coordination of referrals for other community resources and supports:

Please rate the following behavior change techniques in terms of:

- **Not necessary** to use in early childhood evidence-based home visiting to achieve linkages and coordination of referrals for other community resources and supports
- **Useful but not essential** to use in early childhood evidence-based home visiting to achieve linkages and coordination of referrals for other community resources and supports OR
- **Essential** to use in early childhood evidence-based home visiting to achieve linkages and coordination of referrals for other community resources and supports

	Not necessary	Useful but not essential	Essential
14.9 Reduce reward frequency	○	○	○
14.10 Remove punishment	○	○	○
15.1 Verbal persuasion about capability	○	○	○
15.2 Mental rehearsal of successful performance	○	○	○
15.3 Focus on past success	○	○	○
15.4 Self-talk	○	○	○

	Not necessary	Useful but not essential	Essential
16.1 Imaginary punishment	○	○	○
16.2 Imaginary reward	○	○	○
16.3 Vicarious consequences	○	○	○

Reductions in maternal distress (e.g. depression, anxiety, stress)

This section asks about improving maternal health and well-being, specifically **reductions in maternal distress (e.g. depression, anxiety, stress).**

Please rate the following standard practice elements in terms of:

- **Not necessary** to use in early childhood evidence-based home visiting to achieve reductions in maternal distress
- **Useful but not essential** to use in early childhood evidence-based home visiting to achieve reductions in maternal distress OR
- **Essential** to use in early childhood evidence-based home visiting to achieve reductions in maternal distress

Reductions in maternal distress (e.g. depression, anxiety, stress):

Please rate the following standard practice elements in terms of:

- **Not necessary** to use in early childhood evidence-based home visiting to achieve reductions in maternal distress
- **Useful but not essential** to use in early childhood evidence-based home visiting to achieve reductions in maternal distress OR
- **Essential** to use in early childhood evidence-based home visiting to achieve reductions in maternal distress

	Not necessary	Useful but not essential	Essential

	Not necessary	Useful but not essential	Essential
Creating an action plan based on child screenings	○	○	○
Reflect on strategies to support results from caregivers screenings	○	○	○
Home visitor facilitates client connection to cultural and/or spiritual resources	○	○	○
Motivational interviewing	○	○	○
Teaching coping skills to parents	○	○	○
Teaching relaxation/ self-regulation skills to parents	○	○	○

Reductions in maternal distress (e.g. depression, anxiety, stress):

Please rate the following standard practice elements in terms of:

- **Not necessary** to use in early childhood evidence-based home visiting to achieve reductions in maternal distress
- **Useful but not essential** to use in early childhood evidence-based home visiting to achieve reductions in maternal distress OR
- **Essential** to use in early childhood evidence-based home visiting to achieve reductions in maternal distress

	Not necessary	Useful but not essential	Essential
Home visitor shares resources in client's Native language	○	○	○
Providing clients with linkage to services	○	○	○

	Not necessary	Useful but not essential	Essential
Child assessment and screening	○	○	○
Maternal risk assessment and screening	○	○	○
Reflective supervision	○	○	○
Professional development	○	○	○
Proper workloads of staff/ supervisors	○	○	○
Criteria for staff selection are appropriate for population served	○	○	○
Home visitor flexibility/ adaptability	○	○	○
Home visitor sense of humor	○	○	○
Reliable home visitor	○	○	○
Active listening	○	○	○
Relationship building	○	○	○
Responsiveness and sensitivity	○	○	○
Home visitor demonstrates cultural humility	○	○	○

Reductions in maternal distress (e.g. depression, anxiety, stress):

Please rate the following standard practice elements in terms of:

- **Not necessary** to use in early childhood evidence-based home visiting to achieve reductions in maternal distress
- **Useful but not essential** to use in early childhood evidence-based home visiting to achieve reductions in maternal distress OR
- **Essential** to use in early childhood evidence-based home visiting to achieve reductions in maternal distress

	Not necessary	Useful but not essential	Essential
Empathetic communication	O	O	O
Home visitor discipline regarding boundaries and limits of their role	O	O	O
Program trains staff on the prevalence, causes, and consequences of trauma	O	O	O
Program strengthening of service coordination	O	O	O
Home visitor content mastery	O	O	O
Culture of quality for implementing program	O	O	O
Organization/ program collaboration and outreach across the community	O	O	O
Model is based on a parenting framework	O	O	O
Program is data driven	O	O	O
Recruitment of/outreach to parents	O	O	O
Appropriate frequency of visits by the home visitor	O	O	O
Home visitor adaptability with respect to setting and participation	O	O	O
Culturally informed knowledge of the home visitor	O	O	O

	Not necessary	Useful but not essential	Essential
Home visitor understands, affirms, and respects cultural identity of clients	O	O	O
Culturally attuned and responsive approach with all staff training, strategies, materials	O	O	O

Reductions in maternal distress (e.g. depression, anxiety, stress):

The following items list a standard practice element *and* a behavior change technique that share similar definitions. Please rate the following pairs of elements in terms of:

- **Not necessary** to use in early childhood evidence-based home visiting to achieve reductions in maternal distress
- **Useful but not essential** to use in early childhood evidence-based home visiting to achieve reductions in maternal distress OR
- **Essential** to use in early childhood evidence-based home visiting to achieve reductions in maternal distress

	Not necessary	Useful but not essential	Essential
Teaching goal setting skills to parents / Goal setting (behavior)	O	O	O
Teaching problem solving skills to parents / Problem solving	O	O	O
Teaching goal setting skills to parents / Goal setting (outcome)	O	O	O

	Not necessary	Useful but not essential	Essential
Home visitor observation of parent-child interactions / Monitoring of behavior by others without feedback	○	○	○
Processing results from child screenings; Processing results from caregiver screenings / Feedback on behavior	○	○	○
Home visitor providing informal social support for families / Social support (unspecified)	○	○	○
Information sharing (by home visitor to client) / Information about health consequences	○	○	○
Home visitor modeling of desired behaviors / Demonstration of the behavior	○	○	○
Role play/coaching / Behavioral practice/rehearsal	○	○	○
Information sharing (by home visitor to client) / Credible source	○	○	○

Reductions in maternal distress (e.g. depression, anxiety, stress):

Please rate the following behavior change techniques in terms of:

- **Not necessary** to use in early childhood evidence-based home visiting to achieve reductions in maternal distress

- **Useful but not essential** to use in early childhood evidence-based home visiting to achieve reductions in maternal distress OR
- **Essential** to use in early childhood evidence-based home visiting to achieve reductions in maternal distress

	Not necessary	Useful but not essential	Essential
1.4 Action planning	○	○	○
1.5 Review behavior goal(s)	○	○	○
1.6 Discrepancy between current behavior and goal	○	○	○
1.7 Review outcome goal(s)	○	○	○
1.8 Behavioral contract	○	○	○
1.9 Commitment	○	○	○
1.3 Goal setting (outcome)	○	○	○
2.3 Self-monitoring of behavior	○	○	○
2.4 Self-monitoring of outcome(s) of behavior	○	○	○
2.5 Monitoring outcome(s) of behavior by others without feedback	○	○	○
2.6 Biofeedback	○	○	○
2.7 Feedback on outcome(s) of behavior	○	○	○
3.2 Social support (practical)	○	○	○
3.3 Social support (emotional)	○	○	○
4.1 Instruction on how to perform a behavior	○	○	○

Reductions in maternal distress (e.g. depression, anxiety, stress):

Please rate the following behavior change techniques in terms of:

- **Not necessary** to use in early childhood evidence-based home visiting to achieve reductions in maternal distress
- **Useful but not essential** to use in early childhood evidence-based home visiting to achieve reductions in maternal distress OR
- **Essential** to use in early childhood evidence-based home visiting to achieve reductions in maternal distress

	Not necessary	Useful but not essential	Essential
4.2 Information about antecedents	○	○	○
4.3 Re-attribution	○	○	○
4.4 Behavioral experiments	○	○	○
5.2 Salience of consequences	○	○	○
5.3 Information about social and environmental consequences	○	○	○
5.4 Monitoring of emotional consequences	○	○	○
5.5 Anticipated regret	○	○	○
5.6 Information about emotional consequences	○	○	○
6.2 Social comparison	○	○	○
6.3 Information about others' approval	○	○	○
7.1 Prompts/cues	○	○	○
7.2 Cue signalling reward	○	○	○
7.3 Reduce prompts/cues	○	○	○
7.4 Remove access to the reward	○	○	○

	Not necessary	Useful but not essential	Essential
7.5 Remove aversive stimulus	○	○	○

Reductions in maternal distress (e.g. depression, anxiety, stress):

Please rate the following behavior change techniques in terms of:

- **Not necessary** to use in early childhood evidence-based home visiting to achieve reductions in maternal distress
- **Useful but not essential** to use in early childhood evidence-based home visiting to achieve reductions in maternal distress OR
- **Essential** to use in early childhood evidence-based home visiting to achieve reductions in maternal distress

	Not necessary	Useful but not essential	Essential
7.6 Satiation	○	○	○
7.7 Exposure	○	○	○
7.8 Associative learning	○	○	○
8.2 Behavior substitution	○	○	○
8.3 Habit formation	○	○	○
8.4 Habit reversal	○	○	○
8.5 Overcorrection	○	○	○
8.6 Generalisation of a target behavior	○	○	○
8.7 Graded tasks	○	○	○
9.2 Pros and cons	○	○	○
9.3 Comparative imagining of future outcomes	○	○	○
10.1 Material incentive (behavior)	○	○	○
10.2 Material reward(behavior)	○	○	○
10.3 Non-specific reward	○	○	○
10.4 Social reward	○	○	○

Reductions in maternal distress (e.g. depression, anxiety, stress):

Please rate the following behavior change techniques in terms of:

- **Not necessary** to use in early childhood evidence-based home visiting to achieve reductions in maternal distress
- **Useful but not essential** to use in early childhood evidence-based home visiting to achieve reductions in maternal distress OR
- **Essential** to use in early childhood evidence-based home visiting to achieve reductions in maternal distress

	Not necessary	Useful but not essential	Essential
10.5 Social incentive	○	○	○
10.6 Non-specific incentive	○	○	○
10.7 Self-incentive	○	○	○
10.8 Incentive (outcome)	○	○	○
10.9 Self-reward	○	○	○
10.10 Reward (outcome)	○	○	○
10.11 Future punishment	○	○	○
11.1 Pharmacological support	○	○	○
11.2 Reduce negative emotions	○	○	○
11.3 Conserving mental resources	○	○	○
11.4 Paradoxical instructions	○	○	○
12.1 Restructuring the physical environment	○	○	○
12.2 Restructuring the social environment	○	○	○

	Not necessary	Useful but not essential	Essential
12.3 Avoidance/reducing exposure to cues for the behavior	○	○	○
12.4 Distraction	○	○	○

Reductions in maternal distress (e.g. depression, anxiety, stress):

Please rate the following behavior change techniques in terms of:

- **Not necessary** to use in early childhood evidence-based home visiting to achieve reductions in maternal distress
- **Useful but not essential** to use in early childhood evidence-based home visiting to achieve reductions in maternal distress OR
- **Essential** to use in early childhood evidence-based home visiting to achieve reductions in maternal distress

	Not necessary	Useful but not essential	Essential
12.5 Adding objects to the environment	○	○	○
12.6 Body changes	○	○	○
13.1 Identification of self as role model	○	○	○
13.2 Framing/reframing	○	○	○
13.3 Incompatible beliefs	○	○	○
13.4 Valued self-identity	○	○	○
13.5 Identity associated with changed behavior	○	○	○
14.1 Behavior cost	○	○	○
14.2 Punishment	○	○	○
14.3 Remove reward	○	○	○
14.4 Reward approximation	○	○	○

	Not necessary	Useful but not essential	Essential
14.5 Rewarding completion	○	○	○
14.6 Situation-specific reward	○	○	○
14.7 Reward incompatible behavior	○	○	○
14.8 Reward alternative behavior	○	○	○

Reductions in maternal distress (e.g. depression, anxiety, stress):

Please rate the following behavior change techniques in terms of:

- **Not necessary** to use in early childhood evidence-based home visiting to achieve reductions in maternal distress
- **Useful but not essential** to use in early childhood evidence-based home visiting to achieve reductions in maternal distress OR
- **Essential** to use in early childhood evidence-based home visiting to achieve reductions in maternal distress

	Not necessary	Useful but not essential	Essential
14.9 Reduce reward frequency	○	○	○
14.10 Remove punishment	○	○	○
15.1 Verbal persuasion about capability	○	○	○
15.2 Mental rehearsal of successful performance	○	○	○
15.3 Focus on past success	○	○	○
15.4 Self-talk	○	○	○
16.1 Imaginary punishment	○	○	○
16.2 Imaginary reward	○	○	○

	Not necessary	Useful but not essential	Essential
16.3 Vicarious consequences	○	○	○

Reductions in substance use

This section asks about improving maternal health and well-being, specifically **reductions in substance use.**

Please rate the following standard practice elements in terms of:

- **Not necessary** to use in early childhood evidence-based home visiting to achieve reductions in substance use
- **Useful but not essential** to use in early childhood evidence-based home visiting to achieve reductions in substance use OR
- **Essential** to use in early childhood evidence-based home visiting to achieve reductions in substance use

Reductions in substance use:

Please rate the following standard practice elements in terms of:

- **Not necessary** to use in early childhood evidence-based home visiting to achieve reductions in substance use
- **Useful but not essential** to use in early childhood evidence-based home visiting to achieve reductions in substance use OR
- **Essential** to use in early childhood evidence-based home visiting to achieve reductions in substance use

	Not necessary	Useful but not essential	Essential
Creating an action plan based on child screenings	○	○	○

	Not necessary	Useful but not essential	Essential
Reflect on strategies to support results from caregivers screenings	○	○	○
Home visitor facilitates client connection to cultural and/or spiritual resources	○	○	○
Motivational interviewing	○	○	○
Teaching coping skills to parents	○	○	○
Teaching relaxation/ self-regulation skills to parents	○	○	○

Reductions in substance use:

Please rate the following standard practice elements in terms of:

- **Not necessary** to use in early childhood evidence-based home visiting to achieve reductions in substance use
- **Useful but not essential** to use in early childhood evidence-based home visiting to achieve reductions in substance use OR
- **Essential** to use in early childhood evidence-based home visiting to achieve reductions in substance use

	Not necessary	Useful but not essential	Essential
Home visitor shares resources in client's Native language	○	○	○
Providing clients with linkage to services	○	○	○
Child assessment and screening	○	○	○

	Not necessary	Useful but not essential	Essential
Maternal risk assessment and screening	O	O	O
Reflective supervision	O	O	O
Professional development	O	O	O
Proper workloads of staff/ supervisors	O	O	O
Criteria for staff selection are appropriate for population served	O	O	O
Home visitor flexibility/ adaptability	O	O	O
Home visitor sense of humor	O	O	O
Reliable home visitor	O	O	O
Active listening	O	O	O
Relationship building	O	O	O
Responsiveness and sensitivity	O	O	O
Home visitor demonstrates cultural humility	O	O	O

Reductions in substance use:

Please rate the following standard practice elements in terms of:

- **Not necessary** to use in early childhood evidence-based home visiting to achieve reductions in substance use
- **Useful but not essential** to use in early childhood evidence-based home visiting to achieve reductions in substance use OR
- **Essential** to use in early childhood evidence-based home visiting to achieve reductions in substance use

	Not necessary	Useful but not essential	Essential
Empathetic communication	○	○	○
Home visitor discipline regarding boundaries and limits of their role	○	○	○
Program trains staff on the prevalence, causes, and consequences of trauma	○	○	○
Program strengthening of service coordination	○	○	○
Home visitor content mastery	○	○	○
Culture of quality for implementing program	○	○	○
Organization/ program collaboration and outreach across the community	○	○	○
Model is based on a parenting framework	○	○	○
Program is data driven	○	○	○
Recruitment of/outreach to parents	○	○	○
Appropriate frequency of visits by the home visitor	○	○	○
Home visitor adaptability with respect to setting and participation	○	○	○
Culturally informed knowledge of the home visitor	○	○	○

	Not necessary	Useful but not essential	Essential
Home visitor understands, affirms, and respects cultural identity of clients	O	O	O
Culturally attuned and responsive approach with all staff training, strategies, materials	O	O	O

Reductions in substance use:

The following items list a standard practice element *and* a behavior change technique that share similar definitions. Please rate the following pairs of elements in terms of:

- **Not necessary** to use in early childhood evidence-based home visiting to achieve reductions in substance use
- **Useful but not essential** to use in early childhood evidence-based home visiting to achieve reductions in substance use OR
- **Essential** to use in early childhood evidence-based home visiting to achieve reductions in substance use

	Not necessary	Useful but not essential	Essential
Teaching goal setting skills to parents / Goal setting (behavior)	O	O	O
Teaching problem solving skills to parents / Problem solving	O	O	O
Teaching goal setting skills to parents / Goal setting (outcome)	O	O	O

	Not necessary	Useful but not essential	Essential
Home visitor observation of parent-child interactions / Monitoring of behavior by others without feedback	○	○	○
Processing results from child screenings; Processing results from caregiver screenings / Feedback on behavior	○	○	○
Home visitor providing informal social support for families / Social support (unspecified)	○	○	○
Information sharing (by home visitor to client) / Information about health consequences	○	○	○
Home visitor modeling of desired behaviors / Demonstration of the behavior	○	○	○
Role play/coaching / Behavioral practice/rehearsal	○	○	○
Information sharing (by home visitor to client) / Credible source	○	○	○

Reductions in substance use:

Please rate the following behavior change techniques in terms of:

- **Not necessary** to use in early childhood evidence-based home visiting to achieve reductions in substance use

- **Useful but not essential** to use in early childhood evidence-based home visiting to achieve reductions in substance use OR
- **Essential** to use in early childhood evidence-based home visiting to achieve reductions in substance use

	Not necessary	Useful but not essential	Essential
1.4 Action planning	○	○	○
1.5 Review behavior goal(s)	○	○	○
1.6 Discrepancy between current behavior and goal	○	○	○
1.7 Review outcome goal(s)	○	○	○
1.8 Behavioral contract	○	○	○
1.9 Commitment	○	○	○
1.3 Goal setting (outcome)	○	○	○
2.3 Self-monitoring of behavior	○	○	○
2.4 Self-monitoring of outcome(s) of behavior	○	○	○
2.5 Monitoring outcome(s) of behavior by others without feedback	○	○	○
2.6 Biofeedback	○	○	○
2.7 Feedback on outcome(s) of behavior	○	○	○
3.2 Social support (practical)	○	○	○
3.3 Social support (emotional)	○	○	○
4.1 Instruction on how to perform a behavior	○	○	○

Reductions in substance use:

Please rate the following behavior change techniques in terms of:

- **Not necessary** to use in early childhood evidence-based home visiting to achieve reductions in substance use
- **Useful but not essential** to use in early childhood evidence-based home visiting to achieve reductions in substance use OR
- **Essential** to use in early childhood evidence-based home visiting to achieve reductions in substance use

	Not necessary	Useful but not essential	Essential
4.2 Information about antecedents	○	○	○
4.3 Re-attribution	○	○	○
4.4 Behavioral experiments	○	○	○
5.2 Salience of consequences	○	○	○
5.3 Information about social and environmental consequences	○	○	○
5.4 Monitoring of emotional consequences	○	○	○
5.5 Anticipated regret	○	○	○
5.6 Information about emotional consequences	○	○	○
6.2 Social comparison	○	○	○
6.3 Information about others' approval	○	○	○
7.1 Prompts/cues	○	○	○
7.2 Cue signalling reward	○	○	○
7.3 Reduce prompts/cues	○	○	○
7.4 Remove access to the reward	○	○	○

	Not necessary	Useful but not essential	Essential
7.5 Remove aversive stimulus	○	○	○

Reductions in substance use:

Please rate the following behavior change techniques in terms of:

- **Not necessary** to use in early childhood evidence-based home visiting to achieve reductions in substance use
- **Useful but not essential** to use in early childhood evidence-based home visiting to achieve reductions in substance use OR
- **Essential** to use in early childhood evidence-based home visiting to achieve reductions in substance use

	Not necessary	Useful but not essential	Essential
7.6 Satiation	○	○	○
7.7 Exposure	○	○	○
7.8 Associative learning	○	○	○
8.2 Behavior substitution	○	○	○
8.3 Habit formation	○	○	○
8.4 Habit reversal	○	○	○
8.5 Overcorrection	○	○	○
8.6 Generalisation of a target behavior	○	○	○
8.7 Graded tasks	○	○	○
9.2 Pros and cons	○	○	○
9.3 Comparative imagining of future outcomes	○	○	○
10.1 Material incentive (behavior)	○	○	○
10.2 Material reward(behavior)	○	○	○
10.3 Non-specific reward	○	○	○
10.4 Social reward	○	○	○

Reductions in substance use:

Please rate the following behavior change techniques in terms of:

- **Not necessary** to use in early childhood evidence-based home visiting to achieve reductions in substance use
- **Useful but not essential** to use in early childhood evidence-based home visiting to achieve reductions in substance use OR
- **Essential** to use in early childhood evidence-based home visiting to achieve reductions in substance use

	Not necessary	Useful but not essential	Essential
10.5 Social incentive	○	○	○
10.6 Non-specific incentive	○	○	○
10.7 Self-incentive	○	○	○
10.8 Incentive (outcome)	○	○	○
10.9 Self-reward	○	○	○
10.10 Reward (outcome)	○	○	○
10.11 Future punishment	○	○	○
11.1 Pharmacological support	○	○	○
11.2 Reduce negative emotions	○	○	○
11.3 Conserving mental resources	○	○	○
11.4 Paradoxical instructions	○	○	○
12.1 Restructuring the physical environment	○	○	○
12.2 Restructuring the social environment	○	○	○

	Not necessary	Useful but not essential	Essential
12.3 Avoidance/reducing exposure to cues for the behavior	○	○	○
12.4 Distraction	○	○	○

Reductions in substance use:

Please rate the following behavior change techniques in terms of:

- **Not necessary** to use in early childhood evidence-based home visiting to achieve reductions in substance use
- **Useful but not essential** to use in early childhood evidence-based home visiting to achieve reductions in substance use OR
- **Essential** to use in early childhood evidence-based home visiting to achieve reductions in substance use

	Not necessary	Useful but not essential	Essential
12.5 Adding objects to the environment	○	○	○
12.6 Body changes	○	○	○
13.1 Identification of self as role model	○	○	○
13.2 Framing/reframing	○	○	○
13.3 Incompatible beliefs	○	○	○
13.4 Valued self-identity	○	○	○
13.5 Identity associated with changed behavior	○	○	○
14.1 Behavior cost	○	○	○
14.2 Punishment	○	○	○
14.3 Remove reward	○	○	○
14.4 Reward approximation	○	○	○

	Not necessary	Useful but not essential	Essential
14.5 Rewarding completion	◯	◯	◯
14.6 Situation-specific reward	◯	◯	◯
14.7 Reward incompatible behavior	◯	◯	◯
14.8 Reward alternative behavior	◯	◯	◯

Reductions in substance use:

Please rate the following behavior change techniques in terms of:

- **Not necessary** to use in early childhood evidence-based home visiting to achieve reductions in substance use
- **Useful but not essential** to use in early childhood evidence-based home visiting to achieve reductions in substance use OR
- **Essential** to use in early childhood evidence-based home visiting to achieve reductions in substance use

	Not necessary	Useful but not essential	Essential
14.9 Reduce reward frequency	◯	◯	◯
14.10 Remove punishment	◯	◯	◯
15.1 Verbal persuasion about capability	◯	◯	◯
15.2 Mental rehearsal of successful performance	◯	◯	◯
15.3 Focus on past success	◯	◯	◯
15.4 Self-talk	◯	◯	◯
16.1 Imaginary punishment	◯	◯	◯
16.2 Imaginary reward	◯	◯	◯

	Not necessary	Useful but not essential	Essential
16.3 Vicarious consequences	○	○	○

Promotion of positive parenting practices

This section asks about **promotion of positive parenting practices.**

Please rate the following standard practice elements in terms of:

- **Not necessary** to use in early childhood evidence-based home visiting to achieve promotion of positive parenting practices
- **Useful but not essential** to use in early childhood evidence-based home visiting to achieve promotion of positive parenting practices
- **Essential** to use in early childhood evidence-based home visiting to achieve promotion of positive parenting practices

Promotion of positive parenting practices:

Please rate the following standard practice elements in terms of:

- **Not necessary** to use in early childhood evidence-based home visiting to achieve promotion of positive parenting practices
- **Useful but not essential** to use in early childhood evidence-based home visiting to achieve promotion of positive parenting practices
- **Essential** to use in early childhood evidence-based home visiting to achieve promotion of positive parenting practices

	Not necessary	Useful but not essential	Essential
Creating an action plan based on child screenings	○	○	○

	Not necessary	Useful but not essential	Essential
Reflect on strategies to support results from caregivers screenings	○	○	○
Home visitor facilitates client connection to cultural and/or spiritual resources	○	○	○
Motivational interviewing	○	○	○
Teaching coping skills to parents	○	○	○
Teaching relaxation/ self-regulation skills to parents	○	○	○

Promotion of positive parenting practices:

Please rate the following standard practice elements in terms of:

- **Not necessary** to use in early childhood evidence-based home visiting to achieve promotion of positive parenting practices
- **Useful but not essential** to use in early childhood evidence-based home visiting to achieve promotion of positive parenting practices OR
- **Essential** to use in early childhood evidence-based home visiting to achieve promotion of positive parenting practices

	Not necessary	Useful but not essential	Essential
Home visitor shares resources in client's Native language	○	○	○
Providing clients with linkage to services	○	○	○
Child assessment and screening	○	○	○

	Not necessary	Useful but not essential	Essential
Maternal risk assessment and screening	○	○	○
Reflective supervision	○	○	○
Professional development	○	○	○
Proper workloads of staff/ supervisors	○	○	○
Criteria for staff selection are appropriate for population served	○	○	○
Home visitor flexibility/ adaptability	○	○	○
Home visitor sense of humor	○	○	○
Reliable home visitor	○	○	○
Active listening	○	○	○
Relationship building	○	○	○
Responsiveness and sensitivity	○	○	○
Home visitor demonstrates cultural humility	○	○	○

Promotion of positive parenting practices:

Please rate the following standard practice elements in terms of:

- **Not necessary** to use in early childhood evidence-based home visiting to achieve promotion of positive parenting practices
- **Useful but not essential** to use in early childhood evidence-based home visiting to achieve promotion of positive parenting practices
- **Essential** to use in early childhood evidence-based home visiting to achieve promotion of positive parenting practices

	Not necessary	Useful but not essential	Essential
Empathetic communication			
Home visitor discipline regarding boundaries and limits of their role			
Program trains staff on the prevalence, causes, and consequences of trauma			
Program strengthening of service coordination			
Home visitor content mastery			
Culture of quality for implementing program			
Organization/ program collaboration and outreach across the community			
Model is based on a parenting framework			
Program is data driven			
Recruitment of/outreach to parents			
Appropriate frequency of visits by the home visitor			
Home visitor adaptability with respect to setting and participation			
Culturally informed knowledge of the home visitor			

	Not necessary	Useful but not essential	Essential
Home visitor understands, affirms, and respects cultural identity of clients	○	○	○
Culturally attuned and responsive approach with all staff training, strategies, materials	○	○	○

Promotion of positive parenting practices:

The following items list a standard practice element *and* a behavior change technique that share similar definitions. Please rate the following pairs of elements in terms of:

- **Not necessary** to use in early childhood evidence-based home visiting to achieve promotion of positive parenting practices
- **Useful but not essential** to use in early childhood evidence-based home visiting to achieve promotion of positive parenting practices OR
- **Essential** to use in early childhood evidence-based home visiting to achieve promotion of positive parenting practices

	Not necessary	Useful but not essential	Essential
Teaching goal setting skills to parents / Goal setting (behavior)	○	○	○
Teaching problem solving skills to parents / Problem solving	○	○	○
Teaching goal setting skills to parents / Goal setting (outcome)	○	○	○

	Not necessary	Useful but not essential	Essential
Home visitor observation of parent-child interactions / Monitoring of behavior by others without feedback	○	○	○
Processing results from child screenings; Processing results from caregiver screenings / Feedback on behavior	○	○	○
Home visitor providing informal social support for families / Social support (unspecified)	○	○	○
Information sharing (by home visitor to client) / Information about health consequences	○	○	○
Home visitor modeling of desired behaviors / Demonstration of the behavior	○	○	○
Role play/coaching / Behavioral practice/rehearsal	○	○	○
Information sharing (by home visitor to client) / Credible source	○	○	○

Promotion of positive parenting practices:

Please rate the following behavior change techniques in terms of:

- **Not necessary** to use in early childhood evidence-based home visiting to achieve promotion of positive parenting practices

- **Useful but not essential** to use in early childhood evidence-based home visiting to achieve promotion of positive parenting practices OR
- **Essential** to use in early childhood evidence-based home visiting to achieve promotion of positive parenting practices

	Not necessary	Useful but not essential	Essential
1.4 Action planning	○	○	○
1.5 Review behavior goal(s)	○	○	○
1.6 Discrepancy between current behavior and goal	○	○	○
1.7 Review outcome goal(s)	○	○	○
1.8 Behavioral contract	○	○	○
1.9 Commitment	○	○	○
1.3 Goal setting (outcome)	○	○	○
2.3 Self-monitoring of behavior	○	○	○
2.4 Self-monitoring of outcome(s) of behavior	○	○	○
2.5 Monitoring outcome(s) of behavior by others without feedback	○	○	○
2.6 Biofeedback	○	○	○
2.7 Feedback on outcome(s) of behavior	○	○	○
3.2 Social support (practical)	○	○	○
3.3 Social support (emotional)	○	○	○
4.1 Instruction on how to perform a behavior	○	○	○

Promotion of positive parenting practices:

Please rate the following behavior change techniques in terms of:

- **Not necessary** to use in early childhood evidence-based home visiting to achieve promotion of positive parenting practices
- **Useful but not essential** to use in early childhood evidence-based home visiting to achieve promotion of positive parenting practices OR
- **Essential** to use in early childhood evidence-based home visiting to achieve promotion of positive parenting practices

	Not necessary	Useful but not essential	Essential
4.2 Information about antecedents	○	○	○
4.3 Re-attribution	○	○	○
4.4 Behavioral experiments	○	○	○
5.2 Salience of consequences	○	○	○
5.3 Information about social and environmental consequences	○	○	○
5.4 Monitoring of emotional consequences	○	○	○
5.5 Anticipated regret	○	○	○
5.6 Information about emotional consequences	○	○	○
6.2 Social comparison	○	○	○
6.3 Information about others' approval	○	○	○
7.1 Prompts/cues	○	○	○
7.2 Cue signalling reward	○	○	○
7.3 Reduce prompts/cues	○	○	○
7.4 Remove access to the reward	○	○	○

	Not necessary	Useful but not essential	Essential
7.5 Remove aversive stimulus	O	O	O

Promotion of positive parenting practices:

Please rate the following behavior change techniques in terms of:

- **Not necessary** to use in early childhood evidence-based home visiting to achieve promotion of positive parenting practices
- **Useful but not essential** to use in early childhood evidence-based home visiting to achieve promotion of positive parenting practices OR
- **Essential** to use in early childhood evidence-based home visiting to achieve promotion of positive parenting practices

	Not necessary	Useful but not essential	Essential
7.6 Satiation	O	O	O
7.7 Exposure	O	O	O
7.8 Associative learning	O	O	O
8.2 Behavior substitution	O	O	O
8.3 Habit formation	O	O	O
8.4 Habit reversal	O	O	O
8.5 Overcorrection	O	O	O
8.6 Generalisation of a target behavior	O	O	O
8.7 Graded tasks	O	O	O
9.2 Pros and cons	O	O	O
9.3 Comparative imagining of future outcomes	O	O	O
10.1 Material incentive (behavior)	O	O	O
10.2 Material reward(behavior)	O	O	O
10.3 Non-specific reward	O	O	O
10.4 Social reward	O	O	O

Promotion of positive parenting practices:

Please rate the following behavior change techniques in terms of:

- **Not necessary** to use in early childhood evidence-based home visiting to achieve promotion of positive parenting practices
- **Useful but not essential** to use in early childhood evidence-based home visiting to achieve promotion of positive parenting practices OR
- **Essential** to use in early childhood evidence-based home visiting to achieve promotion of positive parenting practices

	Not necessary	Useful but not essential	Essential
10.5 Social incentive	○	○	○
10.6 Non-specific incentive	○	○	○
10.7 Self-incentive	○	○	○
10.8 Incentive (outcome)	○	○	○
10.9 Self-reward	○	○	○
10.10 Reward (outcome)	○	○	○
10.11 Future punishment	○	○	○
11.1 Pharmacological support	○	○	○
11.2 Reduce negative emotions	○	○	○
11.3 Conserving mental resources	○	○	○
11.4 Paradoxical instructions	○	○	○
12.1 Restructuring the physical environment	○	○	○
12.2 Restructuring the social environment	○	○	○

	Not necessary	Useful but not essential	Essential
12.3 Avoidance/reducing exposure to cues for the behavior	○	○	○
12.4 Distraction	○	○	○

Promotion of positive parenting practices:

Please rate the following behavior change techniques in terms of:

- **Not necessary** to use in early childhood evidence-based home visiting to achieve promotion of positive parenting practices
- **Useful but not essential** to use in early childhood evidence-based home visiting to achieve promotion of positive parenting practices OR
- **Essential** to use in early childhood evidence-based home visiting to achieve promotion of positive parenting practices

	Not necessary	Useful but not essential	Essential
12.5 Adding objects to the environment	○	○	○
12.6 Body changes	○	○	○
13.1 Identification of self as role model	○	○	○
13.2 Framing/reframing	○	○	○
13.3 Incompatible beliefs	○	○	○
13.4 Valued self-identity	○	○	○
13.5 Identity associated with changed behavior	○	○	○
14.1 Behavior cost	○	○	○
14.2 Punishment	○	○	○
14.3 Remove reward	○	○	○
14.4 Reward approximation	○	○	○

	Not necessary	Useful but not essential	Essential
14.5 Rewarding completion	○	○	○
14.6 Situation-specific reward	○	○	○
14.7 Reward incompatible behavior	○	○	○
14.8 Reward alternative behavior	○	○	○

Promotion of positive parenting practices:

Please rate the following behavior change techniques in terms of:

- **Not necessary** to use in early childhood evidence-based home visiting to achieve promotion of positive parenting practices
- **Useful but not essential** to use in early childhood evidence-based home visiting to achieve promotion of positive parenting practices OR
- **Essential** to use in early childhood evidence-based home visiting to achieve promotion of positive parenting practices

	Not necessary	Useful but not essential	Essential
14.9 Reduce reward frequency	○	○	○
14.10 Remove punishment	○	○	○
15.1 Verbal persuasion about capability	○	○	○
15.2 Mental rehearsal of successful performance	○	○	○
15.3 Focus on past success	○	○	○
15.4 Self-talk	○	○	○
16.1 Imaginary punishment	○	○	○
16.2 Imaginary reward	○	○	○

	Not necessary	Useful but not essential	Essential
16.3 Vicarious consequences	O	O	O

Reductions in child maltreatment

This section asks about **reductions in child maltreatment.**

Please rate the following standard practice elements in terms of:

- **Not necessary** to use in early childhood evidence-based home visiting to achieve reductions in child maltreatment
- **Useful but not essential** to use in early childhood evidence-based home visiting to achieve reductions in child maltreatment OR
- **Essential** to use in early childhood evidence-based home visiting to achieve reductions in child maltreatment

Reductions in child maltreatment:

Please rate the following standard practice elements in terms of:

- **Not necessary** to use in early childhood evidence-based home visiting to achieve reductions in child maltreatment
- **Useful but not essential** to use in early childhood evidence-based home visiting to achieve reductions in child maltreatment OR
- **Essential** to use in early childhood evidence-based home visiting to achieve reductions in child maltreatment

	Not necessary	Useful but not essential	Essential
Creating an action plan based on child screenings	O	O	O

	Not necessary	Useful but not essential	Essential
Reflect on strategies to support results from caregivers screenings	◯	◯	◯
Home visitor facilitates client connection to cultural and/or spiritual resources	◯	◯	◯
Motivational interviewing	◯	◯	◯
Teaching coping skills to parents	◯	◯	◯
Teaching relaxation/ self-regulation skills to parents	◯	◯	◯

Reductions in child maltreatment:

Please rate the following standard practice elements in terms of:

- **Not necessary** to use in early childhood evidence-based home visiting to achieve reductions in child maltreatment
- **Useful but not essential** to use in early childhood evidence-based home visiting to achieve reductions in child maltreatment OR
- **Essential** to use in early childhood evidence-based home visiting to achieve reductions in child maltreatment

	Not necessary	Useful but not essential	Essential
Home visitor shares resources in client's Native language	◯	◯	◯
Providing clients with linkage to services	◯	◯	◯
Child assessment and screening	◯	◯	◯

	Not necessary	Useful but not essential	Essential
Maternal risk assessment and screening	○	○	○
Reflective supervision	○	○	○
Professional development	○	○	○
Proper workloads of staff/ supervisors	○	○	○
Criteria for staff selection are appropriate for population served	○	○	○
Home visitor flexibility/ adaptability	○	○	○
Home visitor sense of humor	○	○	○
Reliable home visitor	○	○	○
Active listening	○	○	○
Relationship building	○	○	○
Responsiveness and sensitivity	○	○	○
Home visitor demonstrates cultural humility	○	○	○

Reductions in child maltreatment:

Please rate the following standard practice elements in terms of:

- **Not necessary** to use in early childhood evidence-based home visiting to achieve reductions in child maltreatment
- **Useful but not essential** to use in early childhood evidence-based home visiting to achieve reductions in child maltreatment OR
- **Essential** to use in early childhood evidence-based home visiting to achieve reductions in child maltreatment

	Not necessary	Useful but not essential	Essential
Empathetic communication	O	O	O
Home visitor discipline regarding boundaries and limits of their role	O	O	O
Program trains staff on the prevalence, causes, and consequences of trauma	O	O	O
Program strengthening of service coordination	O	O	O
Home visitor content mastery	O	O	O
Culture of quality for implementing program	O	O	O
Organization/ program collaboration and outreach across the community	O	O	O
Model is based on a parenting framework	O	O	O
Program is data driven	O	O	O
Recruitment of/outreach to parents	O	O	O
Appropriate frequency of visits by the home visitor	O	O	O
Home visitor adaptability with respect to setting and participation	O	O	O
Culturally informed knowledge of the home visitor	O	O	O

	Not necessary	Useful but not essential	Essential
Home visitor understands, affirms, and respects cultural identity of clients	O	O	O
Culturally attuned and responsive approach with all staff training, strategies, materials	O	O	O

Reductions in child maltreatment:

The following items list a standard practice element *and* a behavior change technique that share similar definitions. Please rate the following pairs of elements in terms of:

- **Not necessary** to use in early childhood evidence-based home visiting to achieve reductions in child maltreatment
- **Useful but not essential** to use in early childhood evidence-based home visiting to achieve reductions in child maltreatment OR
- **Essential** to use in early childhood evidence-based home visiting to achieve reductions in child maltreatment

	Not necessary	Useful but not essential	Essential
Teaching goal setting skills to parents / Goal setting (behavior)	O	O	O
Teaching problem solving skills to parents / Problem solving	O	O	O
Teaching goal setting skills to parents / Goal setting (outcome)	O	O	O

	Not necessary	Useful but not essential	Essential
Home visitor observation of parent-child interactions / Monitoring of behavior by others without feedback	O	O	O
Processing results from child screenings; Processing results from caregiver screenings / Feedback on behavior	O	O	O
Home visitor providing informal social support for families / Social support (unspecified)	O	O	O
Information sharing (by home visitor to client) / Information about health consequences	O	O	O
Home visitor modeling of desired behaviors / Demonstration of the behavior	O	O	O
Role play/coaching / Behavioral practice/rehearsal	O	O	O
Information sharing (by home visitor to client) / Credible source	O	O	O

Reductions in child maltreatment:

Please rate the following behavior change techniques in terms of:

- **Not necessary** to use in early childhood evidence-based home visiting to achieve reductions in child maltreatment

- **Useful but not essential** to use in early childhood evidence-based home visiting to achieve reductions in child maltreatment OR
- **Essential** to use in early childhood evidence-based home visiting to achieve reductions in child maltreatment

	Not necessary	Useful but not essential	Essential
1.4 Action planning	○	○	○
1.5 Review behavior goal(s)	○	○	○
1.6 Discrepancy between current behavior and goal	○	○	○
1.7 Review outcome goal(s)	○	○	○
1.8 Behavioral contract	○	○	○
1.9 Commitment	○	○	○
1.3 Goal setting (outcome)	○	○	○
2.3 Self-monitoring of behavior	○	○	○
2.4 Self-monitoring of outcome(s) of behavior	○	○	○
2.5 Monitoring outcome(s) of behavior by others without feedback	○	○	○
2.6 Biofeedback	○	○	○
2.7 Feedback on outcome(s) of behavior	○	○	○
3.2 Social support (practical)	○	○	○
3.3 Social support (emotional)	○	○	○
4.1 Instruction on how to perform a behavior	○	○	○

Reductions in child maltreatment:

Please rate the following behavior change techniques in terms of:

- **Not necessary** to use in early childhood evidence-based home visiting to achieve reductions in child maltreatment
- **Useful but not essential** to use in early childhood evidence-based home visiting to achieve reductions in child maltreatment OR
- **Essential** to use in early childhood evidence-based home visiting to achieve reductions in child maltreatment

	Not necessary	Useful but not essential	Essential
4.2 Information about antecedents	◯	◯	◯
4.3 Re-attribution	◯	◯	◯
4.4 Behavioral experiments	◯	◯	◯
5.2 Salience of consequences	◯	◯	◯
5.3 Information about social and environmental consequences	◯	◯	◯
5.4 Monitoring of emotional consequences	◯	◯	◯
5.5 Anticipated regret	◯	◯	◯
5.6 Information about emotional consequences	◯	◯	◯
6.2 Social comparison	◯	◯	◯
6.3 Information about others' approval	◯	◯	◯
7.1 Prompts/cues	◯	◯	◯
7.2 Cue signalling reward	◯	◯	◯
7.3 Reduce prompts/cues	◯	◯	◯
7.4 Remove access to the reward	◯	◯	◯

	Not necessary	Useful but not essential	Essential
7.5 Remove aversive stimulus	○	○	○

Reductions in child maltreatment:

Please rate the following behavior change techniques in terms of:

- **Not necessary** to use in early childhood evidence-based home visiting to achieve reductions in child maltreatment
- **Useful but not essential** to use in early childhood evidence-based home visiting to achieve reductions in child maltreatment OR
- **Essential** to use in early childhood evidence-based home visiting to achieve reductions in child maltreatment

	Not necessary	Useful but not essential	Essential
7.6 Satiation	○	○	○
7.7 Exposure	○	○	○
7.8 Associative learning	○	○	○
8.2 Behavior substitution	○	○	○
8.3 Habit formation	○	○	○
8.4 Habit reversal	○	○	○
8.5 Overcorrection	○	○	○
8.6 Generalisation of a target behavior	○	○	○
8.7 Graded tasks	○	○	○
9.2 Pros and cons	○	○	○
9.3 Comparative imagining of future outcomes	○	○	○
10.1 Material incentive (behavior)	○	○	○
10.2 Material reward(behavior)	○	○	○
10.3 Non-specific reward	○	○	○
10.4 Social reward	○	○	○

Reductions in child maltreatment:

Please rate the following behavior change techniques in terms of:

- **Not necessary** to use in early childhood evidence-based home visiting to achieve reductions in child maltreatment
- **Useful but not essential** to use in early childhood evidence-based home visiting to achieve reductions in child maltreatment OR
- **Essential** to use in early childhood evidence-based home visiting to achieve reductions in child maltreatment

	Not necessary	Useful but not essential	Essential
10.5 Social incentive	○	○	○
10.6 Non-specific incentive	○	○	○
10.7 Self-incentive	○	○	○
10.8 Incentive (outcome)	○	○	○
10.9 Self-reward	○	○	○
10.10 Reward (outcome)	○	○	○
10.11 Future punishment	○	○	○
11.1 Pharmacological support	○	○	○
11.2 Reduce negative emotions	○	○	○
11.3 Conserving mental resources	○	○	○
11.4 Paradoxical instructions	○	○	○
12.1 Restructuring the physical environment	○	○	○
12.2 Restructuring the social environment	○	○	○

	Not necessary	Useful but not essential	Essential
12.3 Avoidance/reducing exposure to cues for the behavior	◯	◯	◯
12.4 Distraction	◯	◯	◯

Reductions in child maltreatment:

Please rate the following behavior change techniques in terms of:

- **Not necessary** to use in early childhood evidence-based home visiting to achieve reductions in child maltreatment
- **Useful but not essential** to use in early childhood evidence-based home visiting to achieve reductions in child maltreatment OR
- **Essential** to use in early childhood evidence-based home visiting to achieve reductions in child maltreatment

	Not necessary	Useful but not essential	Essential
12.5 Adding objects to the environment	◯	◯	◯
12.6 Body changes	◯	◯	◯
13.1 Identification of self as role model	◯	◯	◯
13.2 Framing/reframing	◯	◯	◯
13.3 Incompatible beliefs	◯	◯	◯
13.4 Valued self-identity	◯	◯	◯
13.5 Identity associated with changed behavior	◯	◯	◯
14.1 Behavior cost	◯	◯	◯
14.2 Punishment	◯	◯	◯
14.3 Remove reward	◯	◯	◯
14.4 Reward approximation	◯	◯	◯

	Not necessary	Useful but not essential	Essential
14.5 Rewarding completion	○	○	○
14.6 Situation-specific reward	○	○	○
14.7 Reward incompatible behavior	○	○	○
14.8 Reward alternative behavior	○	○	○

Reductions in child maltreatment:

Please rate the following behavior change techniques in terms of:

- **Not necessary** to use in early childhood evidence-based home visiting to achieve reductions in child maltreatment
- **Useful but not essential** to use in early childhood evidence-based home visiting to achieve reductions in child maltreatment OR
- **Essential** to use in early childhood evidence-based home visiting to achieve reductions in child maltreatment

	Not necessary	Useful but not essential	Essential
14.9 Reduce reward frequency	○	○	○
14.10 Remove punishment	○	○	○
15.1 Verbal persuasion about capability	○	○	○
15.2 Mental rehearsal of successful performance	○	○	○
15.3 Focus on past success	○	○	○
15.4 Self-talk	○	○	○
16.1 Imaginary punishment	○	○	○
16.2 Imaginary reward	○	○	○

	Not necessary	Useful but not essential	Essential
16.3 Vicarious consequences	○	○	○

Reductions in tribally related health disparities

The following section asks about an outcome domain specific to tribal home visiting: **reductions in tribally related health disparities.** While this is not an outcome that is prioritized at the national level, the project team felt that it was important to include in this survey.

Please rate the following standard practice elements in terms of:

- **Not necessary** to use in early childhood evidence-based home visiting to achieve reductions in tribally related health disparities
- **Useful but not essential** to use in early childhood evidence-based home visiting to achieve reductions in tribally related health disparities OR
- **Essential** to use in early childhood evidence-based home visiting to achieve reductions in tribally related health disparities

Reductions in tribally related health disparities:

Please rate the following standard practice elements in terms of:

- **Not necessary** to use in early childhood evidence-based home visiting to achieve reductions in tribally related health disparities
- **Useful but not essential** to use in early childhood evidence-based home visiting to achieve reductions in tribally related health disparities OR
- **Essential** to use in early childhood evidence-based home visiting to achieve reductions in tribally related health disparities

	Not necessary	Useful but not essential	Essential
Creating an action plan based on child screenings	○	○	○

	Not necessary	Useful but not essential	Essential
Reflect on strategies to support results from caregivers screenings	○	○	○
Home visitor facilitates client connection to cultural and/or spiritual resources	○	○	○
Motivational interviewing	○	○	○
Teaching coping skills to parents	○	○	○
Teaching relaxation/ self-regulation skills to parents	○	○	○

Reductions in tribally related health disparities:

Please rate the following standard practice elements in terms of:

- **Not necessary** to use in early childhood evidence-based home visiting to achieve reductions in tribally related health disparities
- **Useful but not essential** to use in early childhood evidence-based home visiting to achieve reductions in tribally related health disparities OR
- **Essential** to use in early childhood evidence-based home visiting to achieve reductions in tribally related health disparities

	Not necessary	Useful but not essential	Essential
Home visitor shares resources in client's Native language	○	○	○
Providing clients with linkage to services	○	○	○
Child assessment and screening	○	○	○

	Not necessary	Useful but not essential	Essential
Maternal risk assessment and screening	○	○	○
Reflective supervision	○	○	○
Professional development	○	○	○
Proper workloads of staff/ supervisors	○	○	○
Criteria for staff selection are appropriate for population served	○	○	○
Home visitor flexibility/ adaptability	○	○	○
Home visitor sense of humor	○	○	○
Reliable home visitor	○	○	○
Active listening	○	○	○
Relationship building	○	○	○
Responsiveness and sensitivity	○	○	○
Home visitor demonstrates cultural humility	○	○	○

Reductions in tribally related health disparities:

Please rate the following standard practice elements in terms of:

- **Not necessary** to use in early childhood evidence-based home visiting to achieve reductions in tribally related health disparities
- **Useful but not essential** to use in early childhood evidence-based home visiting to achieve reductions in tribally related health disparities OR
- **Essential** to use in early childhood evidence-based home visiting to achieve reductions in tribally related health disparities

	Not necessary	Useful but not essential	Essential
Empathetic communication	○	○	○
Home visitor discipline regarding boundaries and limits of their role	○	○	○
Program trains staff on the prevalence, causes, and consequences of trauma	○	○	○
Program strengthening of service coordination	○	○	○
Home visitor content mastery	○	○	○
Culture of quality for implementing program	○	○	○
Organization/ program collaboration and outreach across the community	○	○	○
Model is based on a parenting framework	○	○	○
Program is data driven	○	○	○
Recruitment of/outreach to parents	○	○	○
Appropriate frequency of visits by the home visitor	○	○	○
Home visitor adaptability with respect to setting and participation	○	○	○
Culturally informed knowledge of the home visitor	○	○	○

	Not necessary	Useful but not essential	Essential
Home visitor understands, affirms, and respects cultural identity of clients	○	○	○
Culturally attuned and responsive approach with all staff training, strategies, materials	○	○	○

Reductions in tribally related health disparities:

The following items list a standard practice element *and* a behavior change technique that share similar definitions. Please rate the following pairs of elements in terms of:

- **Not necessary** to use in early childhood evidence-based home visiting to achieve reductions in tribally related health disparities
- **Useful but not essential** to use in early childhood evidence-based home visiting to achieve reductions in tribally related health disparities OR
- **Essential** to use in early childhood evidence-based home visiting to achieve reductions in tribally related health disparities

	Not necessary	Useful but not essential	Essential
Teaching goal setting skills to parents / Goal setting (behavior)	○	○	○
Teaching problem solving skills to parents / Problem solving	○	○	○
Teaching goal setting skills to parents / Goal setting (outcome)	○	○	○

	Not necessary	Useful but not essential	Essential
Home visitor observation of parent-child interactions / Monitoring of behavior by others without feedback	O	O	O
Processing results from child screenings; Processing results from caregiver screenings / Feedback on behavior	O	O	O
Home visitor providing informal social support for families / Social support (unspecified)	O	O	O
Information sharing (by home visitor to client) / Information about health consequences	O	O	O
Home visitor modeling of desired behaviors / Demonstration of the behavior	O	O	O
Role play/coaching / Behavioral practice/rehearsal	O	O	O
Information sharing (by home visitor to client) / Credible source	O	O	O

Reductions in tribally related health disparities:

Please rate the following behavior change techniques in terms of:

- **Not necessary** to use in early childhood evidence-based home visiting to achieve reductions in tribally related health disparities

- **Useful but not essential** to use in early childhood evidence-based home visiting to achieve reductions in tribally related health disparities OR
- **Essential** to use in early childhood evidence-based home visiting to achieve reductions in tribally related health disparities

	Not necessary	Useful but not essential	Essential
1.4 Action planning	○	○	○
1.5 Review behavior goal(s)	○	○	○
1.6 Discrepancy between current behavior and goal	○	○	○
1.7 Review outcome goal(s)	○	○	○
1.8 Behavioral contract	○	○	○
1.9 Commitment	○	○	○
1.3 Goal setting (outcome)	○	○	○
2.3 Self-monitoring of behavior	○	○	○
2.4 Self-monitoring of outcome(s) of behavior	○	○	○
2.5 Monitoring outcome(s) of behavior by others without feedback	○	○	○
2.6 Biofeedback	○	○	○
2.7 Feedback on outcome(s) of behavior	○	○	○
3.2 Social support (practical)	○	○	○
3.3 Social support (emotional)	○	○	○
4.1 Instruction on how to perform a behavior	○	○	○

Reductions in tribally related health disparities:

Please rate the following behavior change techniques in terms of:

- **Not necessary** to use in early childhood evidence-based home visiting to achieve reductions in tribally related health disparities
- **Useful but not essential** to use in early childhood evidence-based home visiting to achieve reductions in tribally related health disparities OR
- **Essential** to use in early childhood evidence-based home visiting to achieve reductions in tribally related health disparities

	Not necessary	Useful but not essential	Essential
4.2 Information about antecedents	◯	◯	◯
4.3 Re-attribution	◯	◯	◯
4.4 Behavioral experiments	◯	◯	◯
5.2 Salience of consequences	◯	◯	◯
5.3 Information about social and environmental consequences	◯	◯	◯
5.4 Monitoring of emotional consequences	◯	◯	◯
5.5 Anticipated regret	◯	◯	◯
5.6 Information about emotional consequences	◯	◯	◯
6.2 Social comparison	◯	◯	◯
6.3 Information about others' approval	◯	◯	◯
7.1 Prompts/cues	◯	◯	◯
7.2 Cue signalling reward	◯	◯	◯
7.3 Reduce prompts/cues	◯	◯	◯
7.4 Remove access to the reward	◯	◯	◯

	Not necessary	Useful but not essential	Essential
7.5 Remove aversive stimulus	○	○	○

Reductions in tribally related health disparities:

Please rate the following behavior change techniques in terms of:

- **Not necessary** to use in early childhood evidence-based home visiting to achieve reductions in tribally related health disparities
- **Useful but not essential** to use in early childhood evidence-based home visiting to achieve reductions in tribally related health disparities OR
- **Essential** to use in early childhood evidence-based home visiting to achieve reductions in tribally related health disparities

	Not necessary	Useful but not essential	Essential
7.6 Satiation	○	○	○
7.7 Exposure	○	○	○
7.8 Associative learning	○	○	○
8.2 Behavior substitution	○	○	○
8.3 Habit formation	○	○	○
8.4 Habit reversal	○	○	○
8.5 Overcorrection	○	○	○
8.6 Generalisation of a target behavior	○	○	○
8.7 Graded tasks	○	○	○
9.2 Pros and cons	○	○	○
9.3 Comparative imagining of future outcomes	○	○	○
10.1 Material incentive (behavior)	○	○	○
10.2 Material reward(behavior)	○	○	○
10.3 Non-specific reward	○	○	○
10.4 Social reward	○	○	○

Reductions in tribally related health disparities:

Please rate the following behavior change techniques in terms of:

- **Not necessary** to use in early childhood evidence-based home visiting to achieve reductions in tribally related health disparities
- **Useful but not essential** to use in early childhood evidence-based home visiting to achieve reductions in tribally related health disparities OR
- **Essential** to use in early childhood evidence-based home visiting to achieve reductions in tribally related health disparities

	Not necessary	Useful but not essential	Essential
10.5 Social incentive	○	○	○
10.6 Non-specific incentive	○	○	○
10.7 Self-incentive	○	○	○
10.8 Incentive (outcome)	○	○	○
10.9 Self-reward	○	○	○
10.10 Reward (outcome)	○	○	○
10.11 Future punishment	○	○	○
11.1 Pharmacological support	○	○	○
11.2 Reduce negative emotions	○	○	○
11.3 Conserving mental resources	○	○	○
11.4 Paradoxical instructions	○	○	○
12.1 Restructuring the physical environment	○	○	○
12.2 Restructuring the social environment	○	○	○

	Not necessary	Useful but not essential	Essential
12.3 Avoidance/reducing exposure to cues for the behavior	◯	◯	◯
12.4 Distraction	◯	◯	◯

Reductions in tribally related health disparities:

Please rate the following behavior change techniques in terms of:

- **Not necessary** to use in early childhood evidence-based home visiting to achieve reductions in tribally related health disparities
- **Useful but not essential** to use in early childhood evidence-based home visiting to achieve reductions in tribally related health disparities OR
- **Essential** to use in early childhood evidence-based home visiting to achieve reductions in tribally related health disparities

	Not necessary	Useful but not essential	Essential
12.5 Adding objects to the environment	◯	◯	◯
12.6 Body changes	◯	◯	◯
13.1 Identification of self as role model	◯	◯	◯
13.2 Framing/reframing	◯	◯	◯
13.3 Incompatible beliefs	◯	◯	◯
13.4 Valued self-identity	◯	◯	◯
13.5 Identity associated with changed behavior	◯	◯	◯
14.1 Behavior cost	◯	◯	◯
14.2 Punishment	◯	◯	◯
14.3 Remove reward	◯	◯	◯
14.4 Reward approximation	◯	◯	◯

	Not necessary	Useful but not essential	Essential
14.5 Rewarding completion	○	○	○
14.6 Situation-specific reward	○	○	○
14.7 Reward incompatible behavior	○	○	○
14.8 Reward alternative behavior	○	○	○

Reductions in tribally related health disparities:

Please rate the following behavior change techniques in terms of:

- **Not necessary** to use in early childhood evidence-based home visiting to achieve reductions in tribally related health disparities
- **Useful but not essential** to use in early childhood evidence-based home visiting to achieve reductions in tribally related health disparities OR
- **Essential** to use in early childhood evidence-based home visiting to achieve reductions in tribally related health disparities

	Not necessary	Useful but not essential	Essential
14.9 Reduce reward frequency	○	○	○
14.10 Remove punishment	○	○	○
15.1 Verbal persuasion about capability	○	○	○
15.2 Mental rehearsal of successful performance	○	○	○
15.3 Focus on past success	○	○	○
15.4 Self-talk	○	○	○
16.1 Imaginary punishment	○	○	○
16.2 Imaginary reward	○	○	○

	Not necessary	Useful but not essential	Essential
16.3 Vicarious consequences	○	○	○

Promotion of connection to culture

The following section asks about an outcome domain specific to tribal home visiting: **promotion of connection to culture.** While this is not an outcome that is prioritized at the national level, the project team felt that it was important to include in this survey.

Please rate the following standard practice elements in terms of:

- **Not necessary** to use in early childhood evidence-based home visiting to achieve promotion of connection to culture
- **Useful but not essential** to use in early childhood evidence-based home visiting to achieve promotion of connection to culture OR
- **Essential** to use in early childhood evidence-based home visiting to achieve promotion of connection to culture

Promotion of connection to culture:

Please rate the following standard practice elements in terms of:

- **Not necessary** to use in early childhood evidence-based home visiting to achieve promotion of connection to culture
- **Useful but not essential** to use in early childhood evidence-based home visiting to achieve promotion of connection to culture OR
- **Essential** to use in early childhood evidence-based home visiting to achieve promotion of connection to culture

	Not necessary	Useful but not essential	Essential

	Not necessary	Useful but not essential	Essential
Creating an action plan based on child screenings	○	○	○
Reflect on strategies to support results from caregivers screenings	○	○	○
Home visitor facilitates client connection to cultural and/or spiritual resources	○	○	○
Motivational interviewing	○	○	○
Teaching coping skills to parents	○	○	○
Teaching relaxation/ self-regulation skills to parents	○	○	○

Promotion of connection to culture:

Please rate the following standard practice elements in terms of:

- **Not necessary** to use in early childhood evidence-based home visiting to achieve promotion of connection to culture
- **Useful but not essential** to use in early childhood evidence-based home visiting to achieve promotion of connection to culture OR
- **Essential** to use in early childhood evidence-based home visiting to achieve promotion of connection to culture

	Not necessary	Useful but not essential	Essential
Home visitor shares resources in client's Native language	○	○	○
Providing clients with linkage to services	○	○	○

	Not necessary	Useful but not essential	Essential
Child assessment and screening	○	○	○
Maternal risk assessment and screening	○	○	○
Reflective supervision	○	○	○
Professional development	○	○	○
Proper workloads of staff/ supervisors	○	○	○
Criteria for staff selection are appropriate for population served	○	○	○
Home visitor flexibility/ adaptability	○	○	○
Home visitor sense of humor	○	○	○
Reliable home visitor	○	○	○
Active listening	○	○	○
Relationship building	○	○	○
Responsiveness and sensitivity	○	○	○
Home visitor demonstrates cultural humility	○	○	○

Promotion of connection to culture:

Please rate the following standard practice elements in terms of:

- **Not necessary** to use in early childhood evidence-based home visiting to achieve promotion of connection to culture
- **Useful but not essential** to use in early childhood evidence-based home visiting to achieve promotion of connection to culture OR
- **Essential** to use in early childhood evidence-based home visiting to achieve promotion of connection to culture

	Not necessary	Useful but not essential	Essential
Empathetic communication	○	○	○
Home visitor discipline regarding boundaries and limits of their role	○	○	○
Program trains staff on the prevalence, causes, and consequences of trauma	○	○	○
Program strengthening of service coordination	○	○	○
Home visitor content mastery	○	○	○
Culture of quality for implementing program	○	○	○
Organization/ program collaboration and outreach across the community	○	○	○
Model is based on a parenting framework	○	○	○
Program is data driven	○	○	○
Recruitment of/outreach to parents	○	○	○
Appropriate frequency of visits by the home visitor	○	○	○
Home visitor adaptability with respect to setting and participation	○	○	○
Culturally informed knowledge of the home visitor	○	○	○

	Not necessary	Useful but not essential	Essential
Home visitor understands, affirms, and respects cultural identity of clients	O	O	O
Culturally attuned and responsive approach with all staff training, strategies, materials	O	O	O

Promotion of connection to culture:

The following items list a standard practice element *and* a behavior change technique that share similar definitions. Please rate the following pairs of elements in terms of:

- **Not necessary** to use in early childhood evidence-based home visiting to achieve promotion of connection to culture
- **Useful but not essential** to use in early childhood evidence-based home visiting to achieve promotion of connection to culture OR
- **Essential** to use in early childhood evidence-based home visiting to achieve promotion of connection to culture

	Not necessary	Useful but not essential	Essential
Teaching goal setting skills to parents / Goal setting (behavior)	O	O	O
Teaching problem solving skills to parents / Problem solving	O	O	O
Teaching goal setting skills to parents / Goal setting (outcome)	O	O	O

	Not necessary	Useful but not essential	Essential
Home visitor observation of parent-child interactions / Monitoring of behavior by others without feedback	O	O	O
Processing results from child screenings; Processing results from caregiver screenings / Feedback on behavior	O	O	O
Home visitor providing informal social support for families / Social support (unspecified)	O	O	O
Information sharing (by home visitor to client) / Information about health consequences	O	O	O
Home visitor modeling of desired behaviors / Demonstration of the behavior	O	O	O
Role play/coaching / Behavioral practice/rehearsal	O	O	O
Information sharing (by home visitor to client) / Credible source	O	O	O

Promotion of connection to culture:

Please rate the following behavior change techniques in terms of:

- **Not necessary** to use in early childhood evidence-based home visiting to achieve promotion of connection to culture

- **Useful but not essential** to use in early childhood evidence-based home visiting to achieve promotion of connection to culture OR
- **Essential** to use in early childhood evidence-based home visiting to achieve promotion of connection to culture

	Not necessary	Useful but not essential	Essential
1.4 Action planning	○	○	○
1.5 Review behavior goal(s)	○	○	○
1.6 Discrepancy between current behavior and goal	○	○	○
1.7 Review outcome goal(s)	○	○	○
1.8 Behavioral contract	○	○	○
1.9 Commitment	○	○	○
1.3 Goal setting (outcome)	○	○	○
2.3 Self-monitoring of behavior	○	○	○
2.4 Self-monitoring of outcome(s) of behavior	○	○	○
2.5 Monitoring outcome(s) of behavior by others without feedback	○	○	○
2.6 Biofeedback	○	○	○
2.7 Feedback on outcome(s) of behavior	○	○	○
3.2 Social support (practical)	○	○	○
3.3 Social support (emotional)	○	○	○
4.1 Instruction on how to perform a behavior	○	○	○

Promotion of connection to culture:

Please rate the following behavior change techniques in terms of:

- **Not necessary** to use in early childhood evidence-based home visiting to achieve promotion of connection to culture
- **Useful but not essential** to use in early childhood evidence-based home visiting to achieve promotion of connection to culture OR
- **Essential** to use in early childhood evidence-based home visiting to achieve promotion of connection to culture

	Not necessary	Useful but not essential	Essential
4.2 Information about antecedents	○	○	○
4.3 Re-attribution	○	○	○
4.4 Behavioral experiments	○	○	○
5.2 Salience of consequences	○	○	○
5.3 Information about social and environmental consequences	○	○	○
5.4 Monitoring of emotional consequences	○	○	○
5.5 Anticipated regret	○	○	○
5.6 Information about emotional consequences	○	○	○
6.2 Social comparison	○	○	○
6.3 Information about others' approval	○	○	○
7.1 Prompts/cues	○	○	○
7.2 Cue signalling reward	○	○	○
7.3 Reduce prompts/cues	○	○	○
7.4 Remove access to the reward	○	○	○

	Not necessary	Useful but not essential	Essential
7.5 Remove aversive stimulus	○	○	○

Promotion of connection to culture:

Please rate the following behavior change techniques in terms of:

- **Not necessary** to use in early childhood evidence-based home visiting to achieve promotion of connection to culture
- **Useful but not essential** to use in early childhood evidence-based home visiting to achieve promotion of connection to culture OR
- **Essential** to use in early childhood evidence-based home visiting to achieve promotion of connection to culture

	Not necessary	Useful but not essential	Essential
7.6 Satiation	○	○	○
7.7 Exposure	○	○	○
7.8 Associative learning	○	○	○
8.2 Behavior substitution	○	○	○
8.3 Habit formation	○	○	○
8.4 Habit reversal	○	○	○
8.5 Overcorrection	○	○	○
8.6 Generalisation of a target behavior	○	○	○
8.7 Graded tasks	○	○	○
9.2 Pros and cons	○	○	○
9.3 Comparative imagining of future outcomes	○	○	○
10.1 Material incentive (behavior)	○	○	○
10.2 Material reward(behavior)	○	○	○
10.3 Non-specific reward	○	○	○
10.4 Social reward	○	○	○

Promotion of connection to culture:

Please rate the following behavior change techniques in terms of:

- **Not necessary** to use in early childhood evidence-based home visiting to achieve promotion of connection to culture
- **Useful but not essential** to use in early childhood evidence-based home visiting to achieve promotion of connection to culture OR
- **Essential** to use in early childhood evidence-based home visiting to achieve promotion of connection to culture

	Not necessary	Useful but not essential	Essential
10.5 Social incentive	○	○	○
10.6 Non-specific incentive	○	○	○
10.7 Self-incentive	○	○	○
10.8 Incentive (outcome)	○	○	○
10.9 Self-reward	○	○	○
10.10 Reward (outcome)	○	○	○
10.11 Future punishment	○	○	○
11.1 Pharmacological support	○	○	○
11.2 Reduce negative emotions	○	○	○
11.3 Conserving mental resources	○	○	○
11.4 Paradoxical instructions	○	○	○
12.1 Restructuring the physical environment	○	○	○
12.2 Restructuring the social environment	○	○	○

	Not necessary	Useful but not essential	Essential
12.3 Avoidance/reducing exposure to cues for the behavior	○	○	○
12.4 Distraction	○	○	○

Promotion of connection to culture:

Please rate the following behavior change techniques in terms of:

- **Not necessary** to use in early childhood evidence-based home visiting to achieve promotion of connection to culture
- **Useful but not essential** to use in early childhood evidence-based home visiting to achieve promotion of connection to culture OR
- **Essential** to use in early childhood evidence-based home visiting to achieve promotion of connection to culture

	Not necessary	Useful but not essential	Essential
12.5 Adding objects to the environment	○	○	○
12.6 Body changes	○	○	○
13.1 Identification of self as role model	○	○	○
13.2 Framing/reframing	○	○	○
13.3 Incompatible beliefs	○	○	○
13.4 Valued self-identity	○	○	○
13.5 Identity associated with changed behavior	○	○	○
14.1 Behavior cost	○	○	○
14.2 Punishment	○	○	○
14.3 Remove reward	○	○	○
14.4 Reward approximation	○	○	○

	Not necessary	Useful but not essential	Essential
14.5 Rewarding completion	○	○	○
14.6 Situation-specific reward	○	○	○
14.7 Reward incompatible behavior	○	○	○
14.8 Reward alternative behavior	○	○	○

Promotion of connection to culture:

Please rate the following behavior change techniques in terms of:

- **Not necessary** to use in early childhood evidence-based home visiting to achieve promotion of connection to culture
- **Useful but not essential** to use in early childhood evidence-based home visiting to achieve promotion of connection to culture OR
- **Essential** to use in early childhood evidence-based home visiting to achieve promotion of connection to culture

	Not necessary	Useful but not essential	Essential
14.9 Reduce reward frequency	○	○	○
14.10 Remove punishment	○	○	○
15.1 Verbal persuasion about capability	○	○	○
15.2 Mental rehearsal of successful performance	○	○	○
15.3 Focus on past success	○	○	○
15.4 Self-talk	○	○	○
16.1 Imaginary punishment	○	○	○
16.2 Imaginary reward	○	○	○

	Not necessary	Useful but not essential	Essential
16.3 Vicarious consequences	○	○	○

End Survey

Name of person who filled out this form

Powered by Qualtrics
