# Supplementary figures and images for: Expert-generated standard practice elements for evidence-based home visiting programs using a Delphi process
Source: PLoS One. 2022 Oct 17;17(10):e0275981. doi: 10.1371/journal.pone.0275981 (PMC9576067; doi:10.1371/journal.pone.0275981)

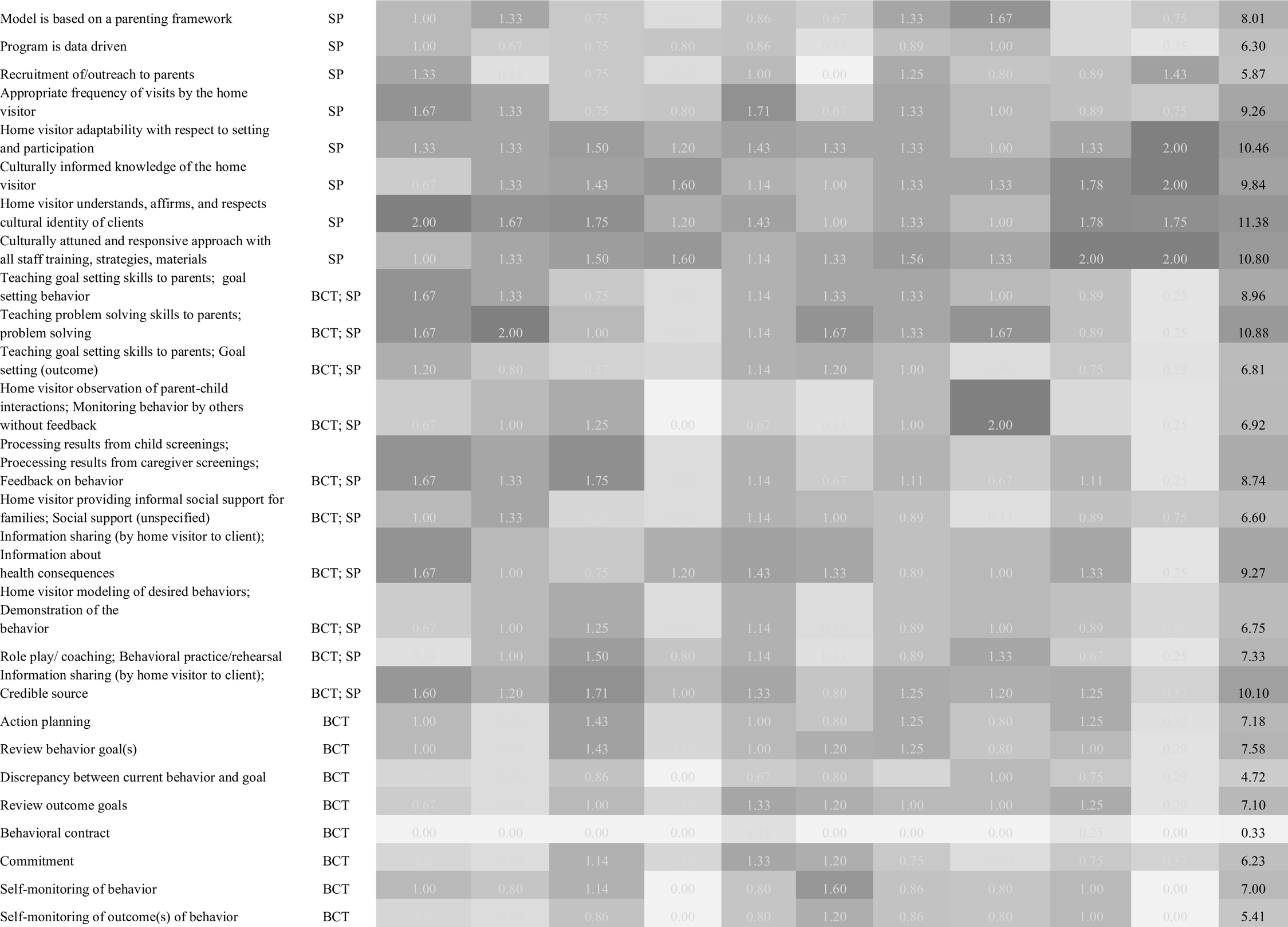

Supplement: S1 Fig — (TIF) [file pone.0275981.s009.tif]

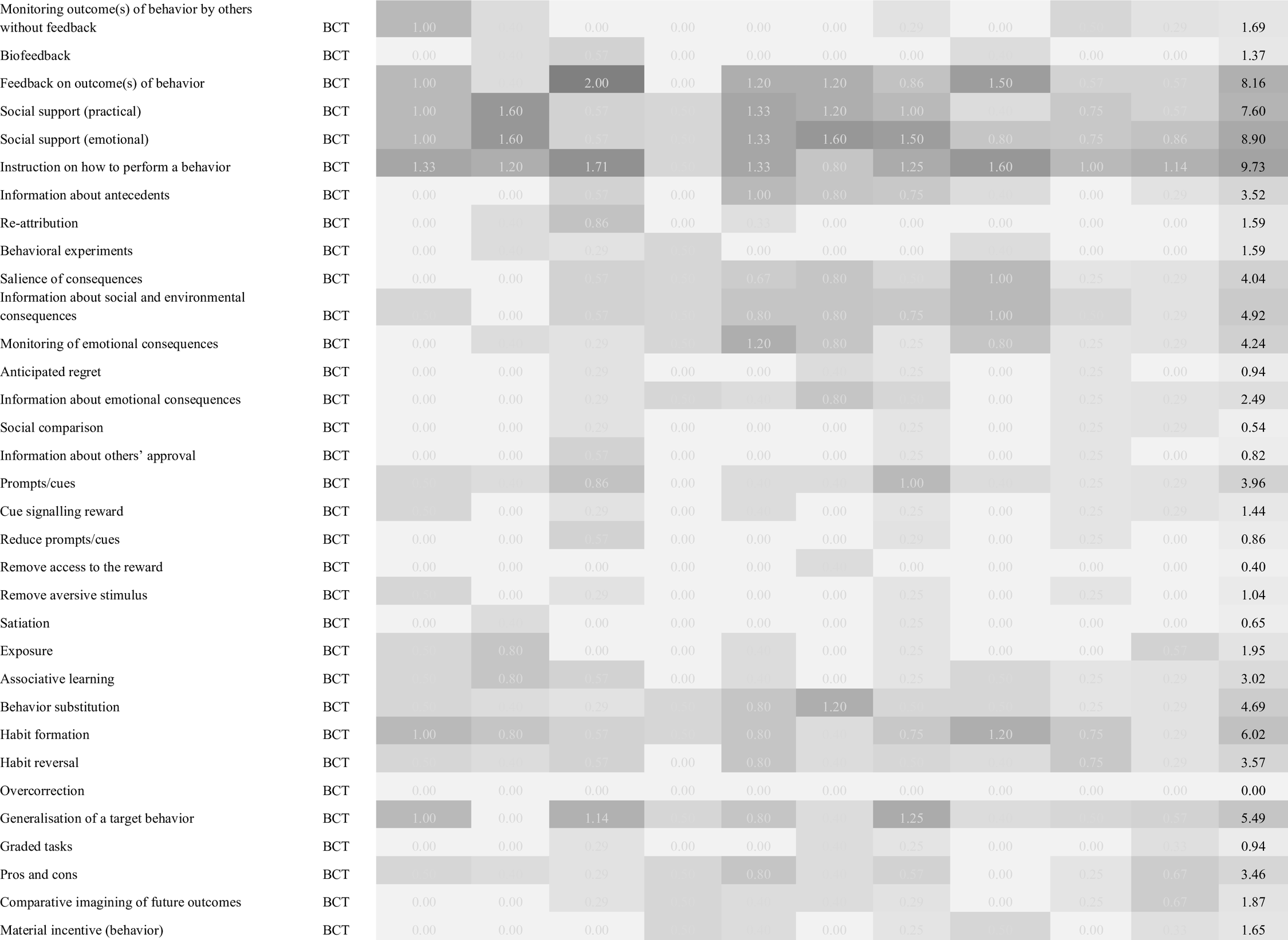

Supplement: S2 Fig — (TIF) [file pone.0275981.s010.tif]

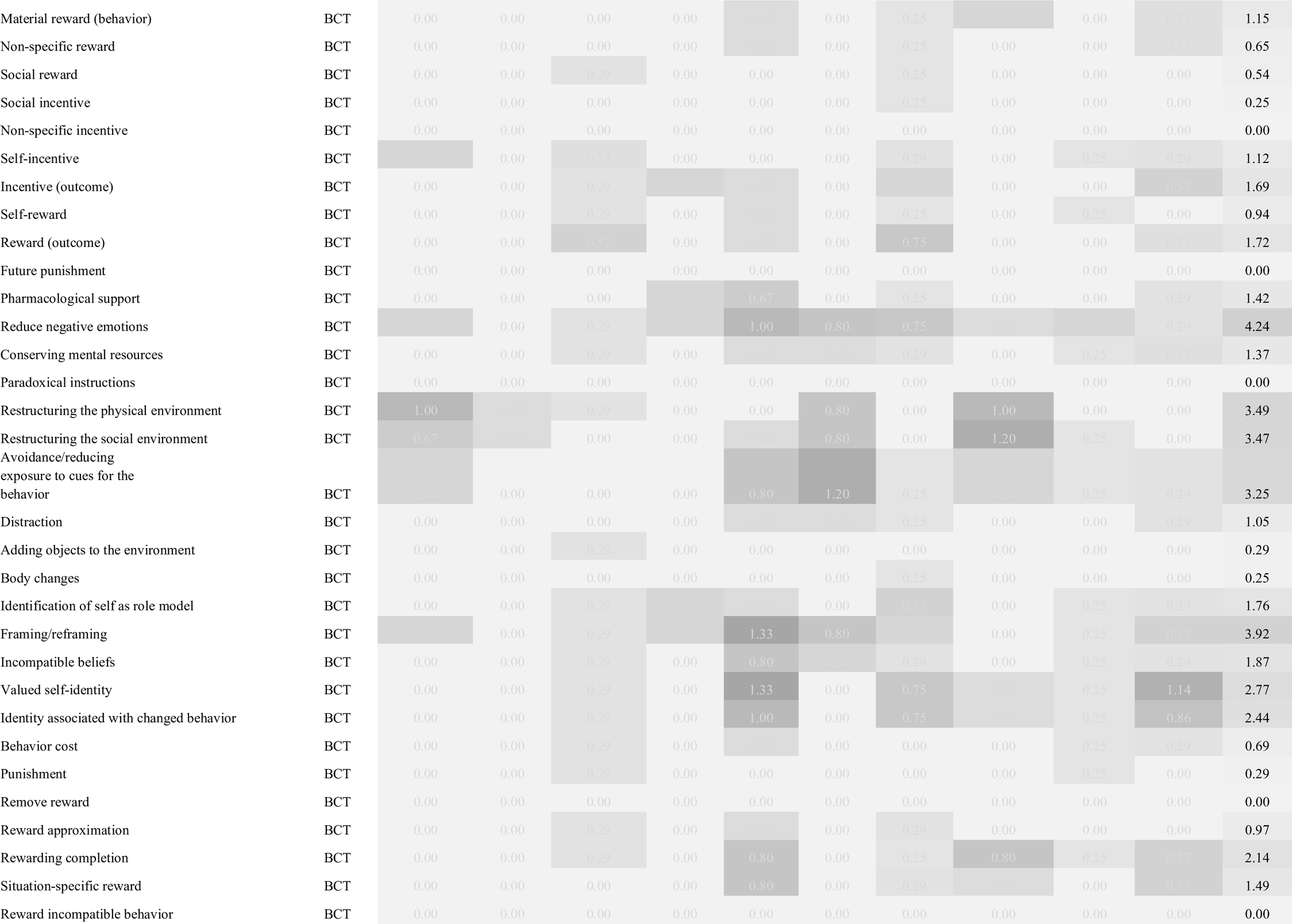

Supplement: S3 Fig — (TIF) [file pone.0275981.s011.tif]

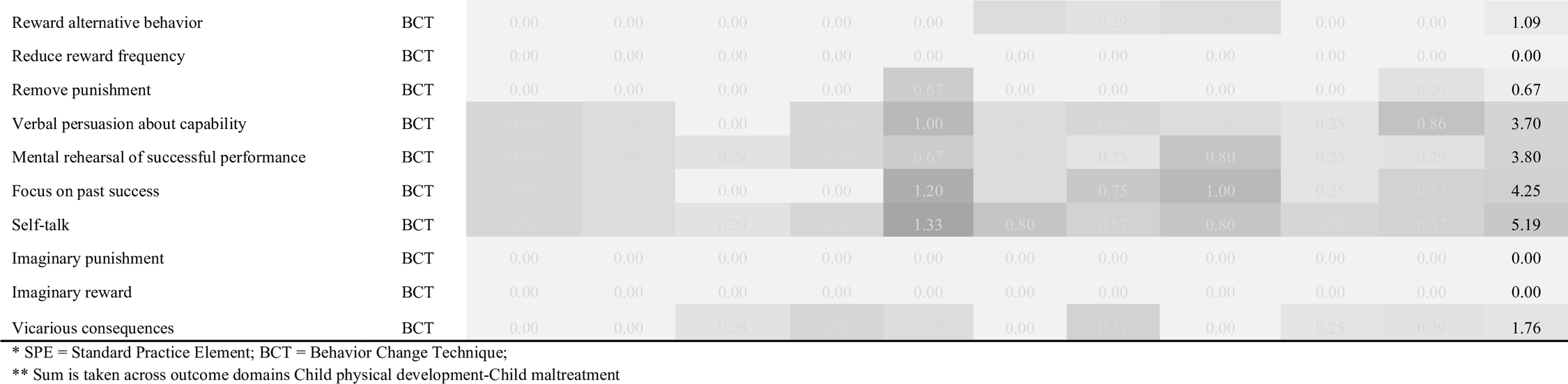

Supplement: S4 Fig — (TIF) [file pone.0275981.s012.tif]
