# Supplementary material for: Expert-generated standard practice elements for evidence-based home visiting programs using a Delphi process
Source: PLoS One. 2022 Oct 17;17(10):e0275981. doi: 10.1371/journal.pone.0275981 (PMC9576067; doi:10.1371/journal.pone.0275981)
Supplement: Attachment — Submitted filename: Response to Reviewers.docx [file pone.0275981.s013.docx]

Mailing Address:

415 N. Washington St.

Baltimore, MD 21203

[eharoz1@jhu.edu](mailto:eharoz1@jhu.edu)

August 29, 2022

Dear Dr. Donlan,

Thank you for the chance to revise our manuscript titled **‘Expert-generated standard practice elements for evidence-based home visiting programs using a modified Delphi process.’** Below you will find our response to the reviewers as well as attached tracked-changed (as a separate file labeled 'Revised Manuscript with Track Changes') and clean versions of the manuscript (labeled ‘Manuscript’).

Sincerely,

Emily E. Haroz, PhD, MA, MHS

Associate Scientist

Department of International Health

Johns Hopkins Bloomberg School of Public Health

**Reply to the journal’s requirements:**

| **Description of the additional requirements** | **Reply by the author(s)** | **Page Number** |
| --- | --- | --- |
| Please ensure that your manuscript meets PLOS ONE's style requirements, including those for file naming. The PLOS ONE style templates can be found at  [https://journals.plos.org/plosone/s/file?id=wjVg/PLOSOne_formatting_sample_main_body.pdf](https://nam02.safelinks.protection.outlook.com/?url=https%3A%2F%2Fjournals.plos.org%2Fplosone%2Fs%2Ffile%3Fid%3DwjVg%2FPLOSOne_formatting_sample_main_body.pdf&data=05%7C01%7Caingalls%40jhu.edu%7Ce40e9cca949b48132bb508da489d29ac%7C9fa4f438b1e6473b803f86f8aedf0dec%7C0%7C0%7C637902137084562857%7CUnknown%7CTWFpbGZsb3d8eyJWIjoiMC4wLjAwMDAiLCJQIjoiV2luMzIiLCJBTiI6Ik1haWwiLCJXVCI6Mn0%3D%7C3000%7C%7C%7C&sdata=KzRHJA7payekQ4TrbHcsrqOuylgLk8TNQx1WfpJzW54%3D&reserved=0) and  [https://journals.plos.org/plosone/s/file?id=ba62/PLOSOne_formatting_sample_title_authors_affiliations.pdf](https://nam02.safelinks.protection.outlook.com/?url=https%3A%2F%2Fjournals.plos.org%2Fplosone%2Fs%2Ffile%3Fid%3Dba62%2FPLOSOne_formatting_sample_title_authors_affiliations.pdf&data=05%7C01%7Caingalls%40jhu.edu%7Ce40e9cca949b48132bb508da489d29ac%7C9fa4f438b1e6473b803f86f8aedf0dec%7C0%7C0%7C637902137084562857%7CUnknown%7CTWFpbGZsb3d8eyJWIjoiMC4wLjAwMDAiLCJQIjoiV2luMzIiLCJBTiI6Ik1haWwiLCJXVCI6Mn0%3D%7C3000%7C%7C%7C&sdata=sh0eP0wDqhE3wmpVgyZVLaJJLKRW%2BeNOaBAJwbGo110%3D&reserved=0). | We have revied the formatting guidelines and ensured the manuscript meets the style requirements. | N/A |
| Please provide additional details regarding participant consent. In the ethics statement in the Methods and online submission information, please ensure that you have specified (1) whether consent was informed and (2) what type you obtained (for instance, written or verbal, and if verbal, how it was documented and witnessed). If your study included minors, state whether you obtained consent from parents or guardians. If the need for consent was waived by the ethics committee, please include this information.  If you are reporting a retrospective study of medical records or archived samples, please ensure that you have discussed whether all data were fully anonymized before you accessed them and/or whether the IRB or ethics committee waived the requirement for informed consent. If patients provided informed written consent to have data from their medical records used in research, please include this information. | We have double checked our documentation of consent and ensured it has been specified in the ethnics statement in the Methods section. | 8 |
| We note that the grant information you provided in the ‘Funding Information’ and ‘Financial Disclosure’ sections do not match.  When you resubmit, please ensure that you provide the correct grant numbers for the awards you received for your study in the ‘Funding Information’ section. | The correct grant number is included in the resubmission. | N/A |
| Please review your reference list to ensure that it is complete and correct. If you have cited papers that have been retracted, please include the rationale for doing so in the manuscript text, or remove these references and replace them with relevant current references. Any changes to the reference list should be mentioned in the rebuttal letter that accompanies your revised manuscript. If you need to cite a retracted article, indicate the article’s retracted status in the References list and also include a citation and full reference for the retraction notice. | We have reviewed the reference list and checked to ensure it is complete. | N/A |

**Reply to the reviewer’s comments:**

| **Description of specific comments/questions of the reviewer** | **Reply by the author(s)** | **Page number** |
| --- | --- | --- |
| This is a well-written and informative article describing the use of a modified Delphi process to generate expert opinions on standard practice elements (SPEs) and behavior change techniques necessary to achieve home visiting outcomes. Overall, I believe the article has much to offer the field of home visiting as explicit discussion of behavior change techniques tend to be limited. My biggest recommendation is to use the wealth of data collected to better chart a course for future work. The paragraph before Limitations gives several potential implications and future directions based on the study findings—the first suggesting that study results can “inform practice” by helping stakeholders personalize their programming. This seems to be one of the biggest future directions—i.e., how can home visiting models create behavior change frameworks that are personalized (keeping with the precision paradigm) and incorporate SPEs that align with behavior change frameworks. I suggest adding more details on exactly how this could be done by home visiting practitioners. Home visiting models that are considered evidenced-based may be reluctant to modify their approaches and the process of personalizing home visiting delivery seems like it would be a very involved process. I think this manuscript would have even greater impact if it expanded on this important point of informing practice and provided more details on what this process should look like, including stakeholders that need to be involved, how evidence-based models could be implemented using a personalized framework, and how home visitors would need to be trained to use a personalized approach. Without these additional details, I worry this article—while very informative and useful for the home visiting field—may not have the desired impact because it does not leave home visiting practitioners with enough food for thought on how their models/programs need to be modified. | We are grateful for the careful and informed review of our manuscript and appreciated the reviewers feedback which as improved our manuscript. As requested, we added more information specifically about how precision approaches could inform practice. This is now included on pp. 23-24 and reads as follows:  *… The SPEs may provide a menu of choices from which to pick to personalize a home visiting program for each family and their unique needs. For example, if a mother scores highly on mental health screening, one of the major goals of their home visiting program might be teach coping skills within the program and to refer and connect them for services. Similarly, if the home visitor is concerned about the child’s development, administering screening, and developing an action plan may be a priority. These practice elements could also be combined if there are multiple, competing priorities. While more research is needed to empirically prove the effectiveness of different SPEs for different outcomes, for now, stakeholders, including program administrators and home visitors, could review home visiting curriculums to identify priority outcomes and ensure the SPEs for those outcomes are included in their current practice.* | pp.23-24 |
| The Introduction is well-written and gives the reader a good sense of the landscape of evidence-based home visiting (EBHV) programs. I do think the comment on lines 80-84 about the challenges in identifying and selecting programs could benefit from slight rewording. My recollection is that the HV evidence review (HOMEVEE) provides a snapshot of each EBHV model, with details on populations served, evidence related to various maternal and child health outcomes, etc. The authors should note this and then perhaps give a bit more explanation on why that level of detail is still insufficient/confusing to stakeholders looking to select a model. | We appreciate the reviewers suggestions and have added more detail briefly describing what Homevee website provides and why it might be complicated from a service provider perspective. | 4 |
| Lines 92-94: Can you indicate who is encouraging program developers to “unravel their approach and identify with greater specificity their key design elements…” Is this something that is being encouraged by MIECHV, researchers, policy-makers, or some combination? | We have updated the text to include who is encouraging the “unraveling”. This can now be found on p. 5. | 5 |
| The top of Figure 1 is blurry and hard to read. | We have saved Figure 1 as a tiff file to ensure high resolution quality. | Figure 1 |
| For readers unfamiliar with Delphi and group consensus techniques, it might be helpful to add another sentence or two when Delphi is first introduced to give a sense of its key characteristics and how it is typically used. A reference would also be helpful. | We added in a sentence briefly describing Delphi methods, including a citation. | 6 |
| Related point—in the Procedures section there is reference to modifying the Delphi process, but I don’t think the reader has enough information on what a “standard” Delphi looks like to be able to understand the modifications made. | We have removed the reference to “modified” as we agree with the reviewer’s points. | 6 and throughout |
| The last part of the Introduction after the paper’s goals are introduced is a little hard to follow. The second goal as written is quite long and there appears to be a typo in the last sentence (“because of the overlapping but funding…”). | We agree with the reviewer and have simplified goal #2. We have also fixed the typo in the last sentence. | 6-7 |
| It is reported that 16 individuals comprised the expert panel, but when describing them it appears 10 were researchers, 7 model reps, and 7 tribal stakeholders. I’m assuming that the three categories overlapped—i.e., someone could be part of two groups—but that should be clarified so that these n’s are aligned. | We have now included a preface to the sentence indicating experts self-identified in multiple rolls. | 7 |
| As I understand the way the Delphi process took place, respondents were asked to generate lists of behavior change techniques necessary for achieving various maternal and child health outcomes relevant to home visiting. What seems to be missing in this approach, however, is understanding how well/to what extent existing evidence-based models incorporate these behavior change technique since it doesn’t appear respondents were asked to use that frame in their responses. I think this is an important limitation and future direction that needs to be addressed. | We thank the reviewer for this suggestion. We fully agree and think this might be a good next step. We have now added this into the limitation section including the suggestion for possible future research. | 24-25 |

**Additional Edits:**

Correction of minor typos and spelling mistakes
